# Identification of genetic modifiers of behavioral phenotypes in serotonin transporter knockout rats

**DOI:** 10.1186/1471-2156-11-37

**Published:** 2010-05-07

**Authors:** Judith Homberg, Isaäc J Nijman, Sylvia Kuijpers, Edwin Cuppen

**Affiliations:** 1 Hubrecht Institute & University Medical Center Utrecht, Uppsalalaan 8, 3584 CT, Utrecht, The Netherlands; 2 Donders Institute for Brain, Cognition, and Behavior, Centre for Neuroscience, Dept. of Cognitive Neuroscience, Radboud University Nijmegen Medical Centre, Geert Grooteplein 21, 6525 EZ Nijmegen, The Netherlands

## Abstract

**Background:**

Genetic variation in the regulatory region of the human serotonin transporter gene (*SLC6A4*) has been shown to affect brain functionality and personality. However, large heterogeneity in its biological effects is observed, which is at least partially due to genetic modifiers. To gain insight into serotonin transporter (SERT)-specific genetic modifiers, we studied an intercross between the Wistar SERT^-/- ^rat and the behaviorally and genetically divergent Brown Norway rat, and performed a QTL analysis.

**Results:**

In a cohort of >150 intercross SERT^-/- ^and control (SERT^+/+^) rats we characterized 12 traits that were previously associated with SERT deficiency, including activity, exploratory pattern, cocaine-induced locomotor activity, and abdominal and subcutaneous fat. Using 325 genetic markers, 10 SERT^-/-^-specific quantitative trait loci (QTLs) for parameters related to activity and exploratory pattern (Chr.1,9,11,14), and cocaine-induced anxiety and locomotor activity (Chr.5,8) were identified. No significant QTLs were found for fat parameters. Using *in silico *approaches we explored potential causal genes within modifier QTL regions and found interesting candidates, amongst others, the 5-HT1D receptor (Chr. 5), dopamine D2 receptor (Chr. 8), cannabinoid receptor 2 (Chr. 5), and genes involved in fetal development and plasticity (across chromosomes).

**Conclusions:**

We anticipate that the SERT^-/-^-specific QTLs may lead to the identification of new modulators of serotonergic signaling, which may be targets for pharmacogenetic and therapeutic approaches.

## Background

The most ancient and highly conserved neuromodulator serotonin (5-HT) plays a critical role in central nervous system processes including, emotion, mood, learning, memory, feeding, sensory processing, and sleep. Not surprisingly, a regulatory variation in the gene encoding the serotonin transporter (5-HTTLPR), the master controller in the fine-tuning of 5-HT signaling, has been associated with a variety of disorders, such as depression, autism, schizophrenia, and eating disorders [1]. However, findings are rather inconsistent across studies. While gene-environment interactions may contribute significantly to the observed phenotypic variation, gene-gene interactions are likely to add considerably as well. Based on the postulation that Quantitative Trait Loci (QTLs), loci on chromosomes that contain trait-linked genetic modifiers, are different in personality traits and disorders [2,3], we argue that identification of serotonin transporter (SERT)-dependent QTLs will lead to both molecular targets for (individualized) pharmacological disease interventions, and molecular targets mediating disease resilience.

It has been established that people with the short (s) allelic variant of the 5-HTTLPR, which is associated with serotonin transporter (5-HTT; *SLC6A4*; human, SERT; rodent) down-regulation compared to the long (l) allelic variant, score higher on neuroticism (emotional instability) and lower on agreeableness (cooperation) [4,5]. These personality traits are related to changes in the functional integrity of brain regions such as the amygdala and anterior cingulate cortex [6]. There are several indications that these personality traits and brain endophenotypes are modified by other major polymorphisms. For example, the MET allelic variant of the *BDNF *(brain-derived neurotrophic factor) VAL66 MET polymorphism was found to protect against 5-HTTLPR-s allele-induced effects on the amygdala and anterior cingulate cortex [7]. Further, we have shown that the MET allelic variant of the *COMT *Met58Val polymorphism positively interacts with the 5-HTTLPR-s allele and exacerbates emotional decision making [8]. Others reported that the *COMT*-MET and 5-HTTLPR-s alleles have an additive effect on amygdala, hippocampus and limbic cortex responsivity to unpleasant stimuli [9], and that the *COMT*-VAL allele in interaction with the 5-HTTLPR-*s *allele raises persistence scores [10]. Also, a significant interaction between the 5-HTTLPR-l allele and the dopamine *D4 receptor*-7 repeat polymorphism in relation to novelty seeking has been reported [11], and a significant interaction between the 5-HTTLPR-s allele and the *DRD4-*7 repeat for harm avoidance [12].

Besides these hypothesis-driven studies, genome-wide association or linkage studies are rising in psychiatry to identify gene × gene interactions in a non-hypothesis driven manner. Yet, environmental factors, which by themselves account for phenotypic variance [13] and interact with the 5-HTTLPR [14], pose difficulties in extracting gene × gene interactions. Experimental laboratory animal studies provide the possibility to control these factors. SERT knockout (SERT^-/-^) rodents are very valuable in modeling the 5-HTTLPR, as they show brain and behavioral endophenotypes that resemble heterogeneity observed for the 5-HTTLPR polymorphism in humans [15-18]. As an example, SERT^-/- ^rodents show a variety of anxiety-related symptoms [15] that are strongly influenced by genetic background [19]. More specifically, it was shown that SERT^+/+ ^and SERT^-/- ^mice on a 129/S6 congenic background did not differ in two behavioral tests for anxiety, while SERT^-/- ^mice on a B6 congenic background showed increased anxiety-like behavioral responses and reduced exploratory locomotion in the very same tests. Based on the human 5-HTTLPR gene interaction reports, we argue that genetic modifier(s) in the 129/S6 background suppress high anxiety behavior in 129/S6 SERT^-/- ^mice. Yet, the identity of such genetic modifiers is unknown thus far.

Here we used the SERT^-/- ^rat to identify genetic modifiers of the SERT gene. The rat was chosen because this species has proven to be an important model organism for human psychiatric disorders, for instance based on the wealth of behavioral tests that have been developed and validated in rats [20]. In addition, the advantage of the rat is the availability of gene knockout models on an outbred background. Although the genetic heterogeneity of these strains is limited compared to human genetic variation, it is more representative for natural genetic variation compared to the inbred mouse strains. Moreover, the phenotypes we have found so far in the SERT^-/- ^rat match those found in SERT^-/- ^mice, and correspond to phenotypes found in humans carrying the s allelic variant of the 5-HTTLPR [17,18,21]. In this study we focus on activity and anxiety in an automated homecage setup, cocaine-induced locomotor activity, and abdominal and subcutaneous white adipose tissue (WAT), parameters that are different between SERT^-/- ^and SERT^+/+ ^rats [18,22,23], cover a broad spectrum of phenotypes and are measurable in a high-throughput manner.

In rat, several QTLs have already been mapped using simple sequence length polymorphism (SSLP) markers [24]. To further optimize QTL mapping we recently generated, and validated, a genome-wide Single Nucleotide Polymorphism (SNP) panel for 632 SNPs that are homozygously polymorphic between the outbred Wistar (WI) and inbred Brown Norway (BN) [25]. These two strains are (phylo)genetically divergent and therefore very useful in a genetic modifier screen based on natural genetic variation. Combined with the robust and high-throughput measurable phenotypes of SERT^-/- ^rats, we aimed to provide a proof of principle for a genetic modifier screen in the rat and to identify SERT^-/- ^selective modifier QTLs that could be explored to prioritize candidate genes modifying SERT gene function. Our results show that the rat is a versatile genetic model to identify QTLs and that there are significant SERT^-/- ^selective QTLs in the genetic backgrounds that were used. Finally, our work revealed interesting novel candidate genes that interact with the serotonergic system function in the rat.

## Results

### Crossings

To create SERT^+/+ ^and SERT^-/- ^rats with mixed WI/BN background we mated two male BN rats with five female SERT^-/- ^rats. Among the resulting SERT^+/- ^F1 generation we conducted brother-sister matings to produce an F2 population of 166 animals (Additional file 1 Table S1). Because WI rats have a white fur and BN rats have a brown-black fur, the F2 population had mixed fur colors. The majority of the animals was either white or black, and about 1/10 of the animals was white with black spots. Because the Phenotyper had difficulties with tracking the white/black animals were not included.

### Analyses of phenotypes

**Figure 1:**
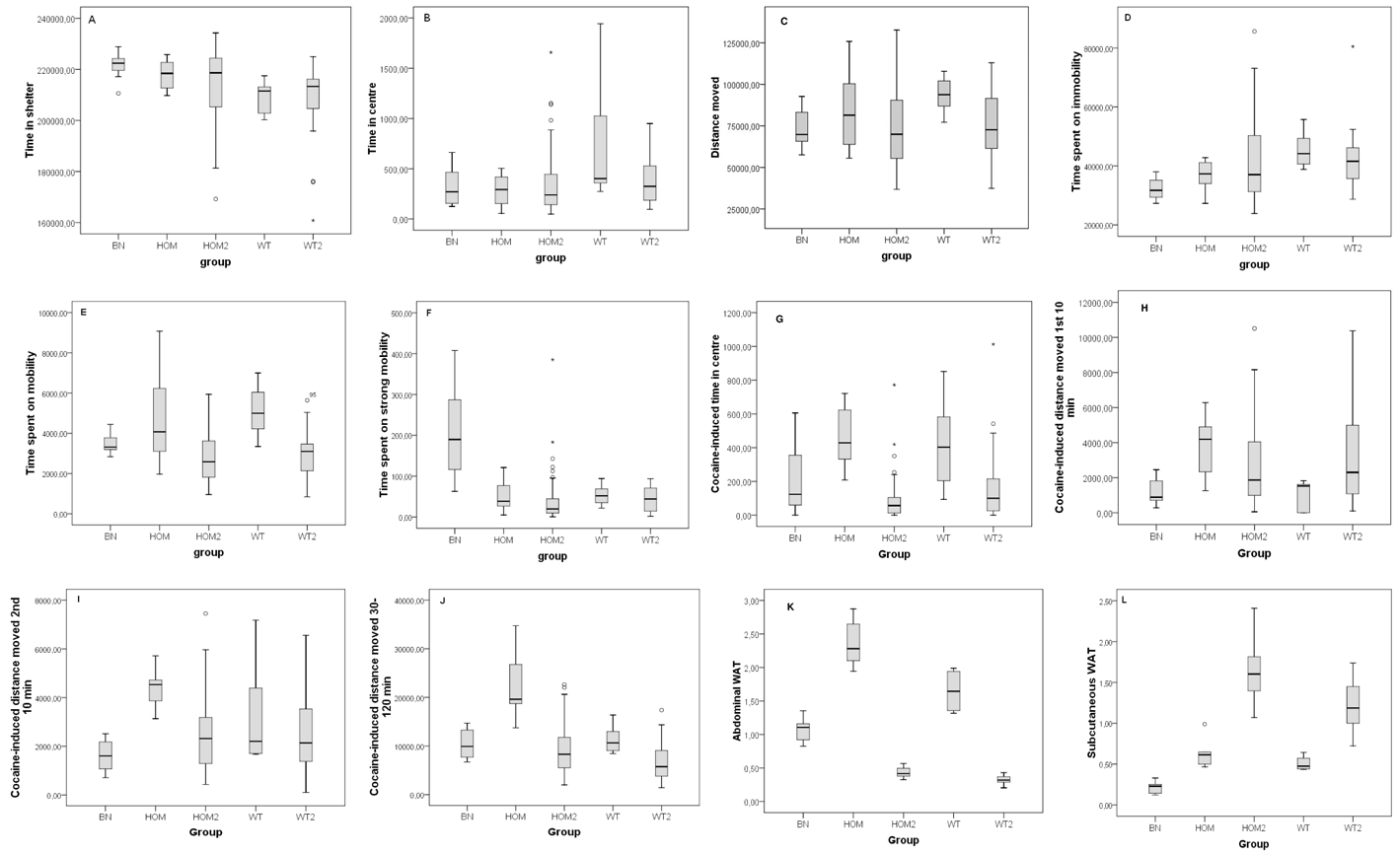
**Phenotype ranges for traits recorded in the 3-day Phenotyper test, the cocaine-induced locomotor test, and for WAT traits**. Solid boxes represent the 50% of trait values closest to the median (bold line). The dashed lines represent the low and high 25% of trait values. Genotypes: BN = parental BN; HOM = parental SERT^-/-^; HOM2 = F2 SERT^-/-^; WT = parental SERT^+/+^; WT2 = F2 SERT^+/+^

We recorded a total of 12 phenotypic traits in the F0 and F2 animals, including measurements for anxiety, locomotor activity, exploratory pattern, cocaine-induced locomotor activity (males only) and abdominal and subcutaneous WAT (females only) (Figure 1, Additional file 1 Tables S2-7). When testing the parental SERT^+/+^, SERT^-/- ^and BN animals we found that SERT^-/- ^rats compared to SERT^+/+ ^rats showed significant differences for anxiety/locomotor activity (time time in shelter), exploratory pattern (immobility), cocaine-induced locomotor activity [22], and abdominal WAT deposition [23]. We also found significant Wistar-BN strain differences for anxiety/locomotor activity (time in shelter), exploratory pattern (immobility and strong mobility), cocaine sensitivity and abdominal/subcutaneous WAT. In addition, significant genotype/strain effects were found for anxiety (time in centre) and locomotor activity (distance moved), but these parameters did not survive a bonferroni correction for multiple testing. Overall, these data indicate that SERT^-/- ^rats, compared to SERT^+/+ ^rats, are more anxious [18] and explore by ceasing movement and scanning of their environment. Moreover, the data show that BN rats generally move less, but move faster or more sudden whenever they move. Also WAT is clearly reduced in BN rats. By contrast, genotype differences for anxiety, activity and exploratory patterns were absent in F2 SERT^+/+ ^and SERT^-/- ^animals, while cocaine-induced anxiety and abdominal WAT were significantly increased in F2 SERT^-/- ^rats compared to SERT^+/+ ^rats.

#### Correlations between phenotypes

**Figure 2:**
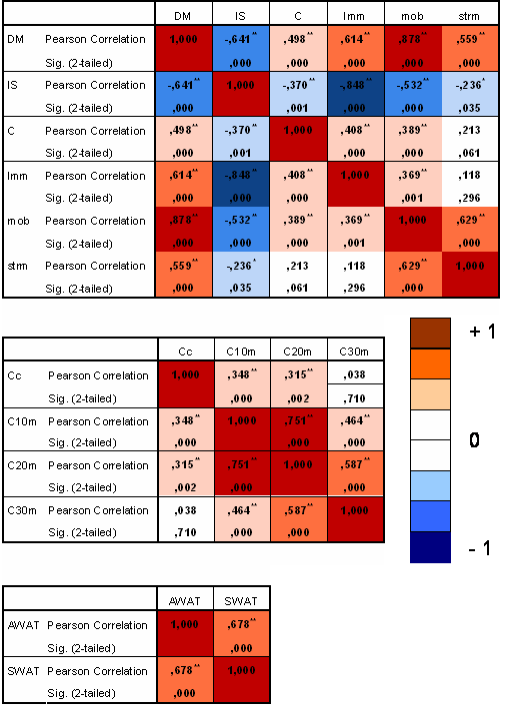
**Pearson's correlation coefficients between phenotypes across F2 rats**. **p < 0.004 (Bonferroni corrected). Phenotyper: DM = distance moved; IS = time in shelter; C = time in centre; Imm = time spent on immobility; mob = time spent on mobility; strm = time spent on strong mobility. Cocaine: Cc = Time in centre, C10 = distance moved during 1^st ^10 min; C20 = distance moved during 2^nd ^10 min; C30 = distance moved during 30-120 min White adidpose tissue (WAT): AWAT = abdominal WAT; SWAT = subcutaneous WAT

There are several phenotypic differences between SERT^+/+ ^and SERT^-/- ^rats. If these differences are caused by the same genetic loci, they should be correlated in the F2 animals. As illustrated in figure 2, we found significant correlations between different measures within the three separate tests. Time in shelter and time in centre of the Phenotyper correlated significantly with distance moved and exploration-related parameters (immobility and mobility), indicating that the level of anxiety is strongly influenced by activity in the SERT^-/- ^rats. Interestingly, cocaine-induced anxiety (time in centre) was not correlated with the duration of the cocaine-induced locomotor response (30-120 min after cocaine challenge), suggesting that the anxiogenic effects of cocaine are particularly associated with the immediate cocaine effects.

#### QTL analysis

The overall QTL analysis by CIM, including all genotypes and sexes revealed two very strong linkage intervals on chromosome 1 and 10 (data not shown). The linkage interval at chromosome 1 (Chr.1) is located between 63 and 92 cM (LOD score = 41.8) and contains the *Tyr *gene, which encodes the enzyme tyrosinase that catalyzes the conversion of tyrosine to DOPAquinone in melanin biosynthesis and is responsible for the white coat color of albino rats. This QTL is consistent with the mixture of black and white coat colors among the F2 animals. Further, at Chr.10, between 67.13 - 67.155 cM, there is a peak with a LOD score of 27.5 that contains the *Slc6a4 *(SERT) gene (ENSRNOG00000003476), consistent with the SERT^-/- ^and SERT^+/+ ^genotypes in our test sample. Because no y-chromosome markers were included in the screen, no QTL was found for sex.

When we conducted the CIM for genotypes separately we identified 10 QTLs in the SERT^-/- ^background (table 1). We obtained large effect sizes for cocaine-induced time in centre and the duration of cocaine-induced locomotor activity (38%), while effect sizes were lower for time in shelter (2.5%), distance moved (6%) and mobility (19%) in the Phenotyper. No significant QTLs for time in centre (anxiety), immobility, strong mobility, and WAT were found. Also SERT^+/+^-linked QTLs were found (data not shown). They did not overlap with SERT^-/- ^associated QTLs, indicating that the SERT^-/- ^associated QTLs are SERT gene specific.

**Table 1:** SERT^-/- ^specific QTLs

trait	Chr	LOD score	GWSL	Permut	region	bp	Ensembl	Nr of genes
Time in shelter (2.5%)	1	**4,55**	3,58	4000	44878370-53396511	8.518.141	1:44878370:53396511:1	36
	1	**4,2**			118843159-131711026	12.867.867	1:118843159:131711026:1	32
	9	**7,06**			81816446-99976905	18.160.459	9:81816446:99976905:1	97
	11	**4,78**			64497121-77560490	13.063.369	11:64497121:77560490:1	65
	14	**6,77**			50613160-62880129	12.266.969	14:50613160:62880129:1	18
Distance moved (6%)	9	**4,47**	4,16	5000	81816446-99976905	18.160.459	9:81816446:99976905:1	97
Mobility (19%)	9	**4,49**	4,05	10000	81816446-99976905	18.160.459	9:81816446:99976905:1	97
Coc-time in centre (38%)	8	**4,03**	3,96	4000	44043629-57318916	13.275.287	8:44043629:57318916:1	80
Coc-30-120 min (38%)	5	**4,16**	4,08	4000	150949241-161571992	10.622.751	5:150949241:161571992:1	120
	8	3,45			57318916-65095744	7.776.828	8:57318916:65095744:1	61

Chr = chromosome; GWSL = Genome Wide significance Level; permut = number of permutations; bp = basepairs

Bold LOD scores are significant'; percentages indicate effect sizes.

For each of the significant LOD scores, we further explored candidate genes using the Ensembl BioMart tool. We took the estimated location of the QTL on the chromosome and explored genes around the associated markers within the region determined by the adjacent non-significant markers. Although this is a rather rough approach, and QTLs on gene rich chromosomes may lead to aspecific candidate genes, it provides indices for altered pathways that could be pinpointed in future research. We took the intervals rather broadly because the marker locations had been recalculated from physical coordinates to genetic coordinates, which could be associated with deviations. If we would have taken a narrow range we may have missed potential candidate genes. As shown in additional file 1 table S8 (see table 1 for total number of genes per QTL), the most apparent ones involve the 5-HT1D and cannabinoid receptor 2 on Chr. 5 for the QTL that is linked to cocaine-induced locomotor activity in SERT^-/- ^rats. Further, among the candidate genes associated with the QTL for cocaine-induced anxiety on Chr. 8 was the dopamine D2 receptor, locomotor-associated parameters in the Phenotyper were associated with acetylcholine receptor subunit precursors (Chr. 9), and time in shelter was associated with the cholecystokinin receptor type A gene (Chr. 14). We also found genes involved in development, for instance ectoderm-neural cortex protein 2 (Chr. 1), insulin-like growth factor 1 receptor precursor (Chr. 14), fibroblast growth factor 12 (Chr. 11), cell adhesion molecule 1 (Chr.8), beta-secretase 1 precursor (Chr. 8), caspase 9, apoptosis-related cysteine peptidase (Chr. 5), and ephrin receptor EphA2 (Chr. 5). Finally, the phosphatidylinositol 3-kinase-Akt signalling pathway (tyrosine-protein phosphatase non-receptor type 9; Serine/Threorine kinases and phoshatases; 1-phosphatidylinositol-4,5-bisphosphate phosphatases) may modify SERT^-/- ^traits.

As a final part of the genetic modifier screen we explored whether the SERT^-/- ^QTLs overlap with known QTLs for rat. We used the RGD, and found one QTL that overlaps with a SERT^-/- ^specific QTL: anxiety QTL 13 (84023578-129023578 bp [26]) is located in the region of the SERT^-/- ^QTL on Chr.1 that is linked to time in shelter (Additional file 1 table S8).

## Discussion

Here we provide proof of principle for a rat QTL analysis and identified significant SERT^-/- ^specific QTLs, suggesting that there are genes throughout the genome interacting with the SERT gene in rat. In addition, we have created an overview of candidate genes within QTL regions that may interact with SERT, including genes encoding 1] the 5-HT1D receptor, 2] components of other neurotransmitter systems, or 3] developmental and plasticity proteins.

We used BN rats to introduce genetic variance because this strain is genetically most divergent from WI rats [27], as confirmed by a phylogenetic analysis based on SNP genotyping of a panel of inbred rat strains [25]. In this study we show that the two strains also differ for behavioral traits and WAT. Overall, BN rats are less active, but when they are active, their behavioral acts seem to be more sudden and 'energetic'. Also, their anxiety level, as measured by their avoidance of the centre of the test cage seems to parallel that of SERT^-/- ^rats [18], although significant effects were lost after Bonferroni correction. Further, it is apparent that BN rats are lean and exhibit less WAT, which may relate to their rather close relation to the wild rat [25]. BN rats are thus far mainly used as a reference strain for research on hypertension, inflammation and glucose metabolism http://www.rgd.mcw.edu.. Our findings indicate that the application of BN rats can be extended to basic behavioral characteristics as well cocaine sensitivity and WAT. BN rats are used to generate rat Recombinant Inbred (RI) lines, together with the spontaneously hypertensive rat (SHR), and for congenic lines using Dahl Salt Sensitive (SS) or Fawn Hooded Hypertensive (FHH) rat strains, and it may therefore be worthwhile to characterize these lines for anxiety, aberrant exploratory behavior, cocaine sensitivity, and WAT deposition. We further show that BN alleles change the differential phenotypes of SERT^+/+ ^and SERT^-/- ^rats. For instance, while parental SERT^-/- ^rats spent less time in the shelter of the Phenotyper (effect size 2.5%) SERT^-/- ^and SERT^+/+ ^with mixed BN/WI alleles did not differ for this parameter. Further, mobility was strongly reduced in the F2 population compared to the parental animals, which is reflected in the relatively high effect size for this parameter (19%). The parental genotype differences for the locomotor response during the first 20 min and last 30-120 min of the cocaine challenge were not seen in F2 animals. Vice versa, among F2 animals cocaine-induced anxiety (time spent in centre) was increased in SERT^-/- ^rats, which was not seen in the parental animals. Apparently, differential genetic modifiers may be involved in the onset of cocaine's psychomotor effects, the duration of the response, and the anxiogenic effects of cocaine. The effect sizes of 38% for these parameters indicate that genetic modifiers explain a considerable amount of the variance in the locomotor response to cocaine in SERT^-/- ^rats. Abdominal WAT was increased in both parental and F2 SERT^-/- ^rats compared to SERT^+/+ ^rats, while F2, but not parental, SERT^-/- ^rats also exhibited increased subcutaneous WAT. Again, there are clear interactions between BN and WI alleles.

To identify QTLs responsible for these behavioral and WAT changes we performed a CIM, which fits parameters for a target QTL in one interval while simultaneously fitting partial regression coefficients for "background markers" to account for variance caused by non-target QTL. This leads to the estimation of both the positions and relative effects of putative QTLs on the chromosome. CIM is strongly dependent on the choice of suitable marker loci to serve as background markers. Since our markers are evenly distributed over the genome with 2-10 cM intervals [25], risk for bias towards certain QTLs is expected to be limited. The power of the QTL analysis may be affected by the number of SNP markers we used, but it has been shown that the power of QTL detection and the standard errors of genetic effect estimates are little affected by an increase of marker density beyond 10 cM [27]. Indeed, despite the limited number of SNP markers, we identified in the overall QTL analysis the *Tyr *gene for coat color and the *Slc6a4 *(SERT) gene, indicating that the SNP panel and CIM approach are valid. Further, several significant SERT^-/- ^dependent QTLs were identified (table 1) for the Phenotyper-based data and cocaine-induced locomotor activity, indicating that these parameters are susceptible to SERT-related genetic modifiers. However, no significant LOD scores were found for WAT parameters. Because of the strong genotype differences in both the P0 and F2 generations, we argue that WAT is strongly influenced by the SERT itself, and not (or to a limited extend) by genetic modifiers. We identified 10 significant QTLs for SERT^-/- ^rats. The behavioral traits link to chromosome 1, 5, 8, 9, 11, and 14. The QTL on chromosome 9 is linked to mobility, distance moved and time in shelter, suggesting that these traits are associated. Indeed, the correlation analysis revealed significant correlations between these parameters (figure 2). Potentially, this QTL links to locomotor activity, and as such, time spent in the shelter could be related to activity or perhaps an altered circadian rhythm. Since time in shelter was linked to multiple QTLs, this parameter may also be influenced by other processes. Further, cocaine-induced anxiety was linked to a QTL on Chr. 8 and the duration of cocaine-induced locomotor activity was linked to a QTL on Chr. 5, which is consistent with the lack of a correlation between the two (figure 2).

Among the candidate genetic modifiers underlying the QTL for cocaine-induced locomotor activity is the 5-HT1D receptor. The 5-HT1D receptor is a G protein-coupled autoreceptor [28] that is particularly involved in the regulation of cranial vasoconstriction. Thereby, 5-HT1D agonists have efficacy in the treatment of headache and migraine [29]. It is tempting to speculate that genotype-dependent differences in vasoconstriction influence the transport of cocaine in the brain. Also the cannabinoid receptor 2 (Chr. 5) was linked to cocaine-induced locomotor activity, which may be related to its modulatory role in regulation of SERT activity [30]. A candidate gene associated with cocaine-induced anxiety (time in centre) is the dopamine D2 receptor (Chr. 8). The dopamine D2 receptor mediates both the rewarding and anxiogenic effects of cocaine [31], and there are interactions between the dopamine D2 receptor and SERT in mediating cocaine reward [32], both of which may modify SERT^-/- ^rat behavioral responses to cocaine. Across genes, we also found candidate genes involved in development and plasticity. Thus, altered neuronal differentiation (ectoderm-neural cortex protein 2 (Chr. 1)), together with altered cell division (fibroblast growth factor 12 (Chr. 11)), cell differentiation (Insulin-like growth factor I receptor precursor (Chr. 14)), apoptosis (apoptosis-related cysteine peptidase (Chr. 5), caspase 9 (Chr. 9)), vascular development (ephrin receptor EphA2 (Chr. 5), cell adhesion molecule 1 (Chr.8)), and the formation of myelin sheets (beta-secretase 1 precursor (Chr. 8)), may shape the organization of brain networks and thereby their functions. In this context it is important to note that 5-HT exerts several neurotrophic actions during early brain development [33]. Defective clearance of 5-HT as observed in the SERT^-/- ^mice and rats causes a failure in the maturation of the thalamocortical neurons [34], an abnormal structural organization in the somatosensory cortex [35] and altered morphology of pyramidal neurons in the prefrontal cortex and amygdala [16]. In humans the low function variant of the 5-HTTLPR is associated with macroscopic structural and functional changes in corticolimbic regions such as the prefrontal cortex, hippocampus and amygdala [6,36,37]. The genes involved in development and plasticity may be the target of well-established signaling pathways such as the phosphatidylinositol 3-kinase-Akt signaling pathway that mediates cell growth, differentiation and survival [38]. Of interest, a recent study found an epistatic interaction of *Slc6a4 *and *Pten *for macrocephaly in mice [39]. Given that Pten is a key modulator of the phosphatidylinositol 3-kinase-Akt signalling pathway, our findings may support the idea that 5-HT modulates brain development via this pathway.

Some QTLs have been submitted at the RGD, but only one overlaps with a SERT^-/- ^specific QTL identified in this study. Critical is that the WI rat has previously not been used in QTL analyses [40-42], which hampers the direct comparison between QTLs. Nonetheless, anxiety QTL 13 on Chr.1 of Wistar-Kyoto rats [26] matches the time in shelter parameter in SERT^-/- ^rats. This finding suggests that the WI-derived rat strain that was used in the previous study [26] had a sensitized serotonergic background.

Our study has some limitations that have to be mentioned. First, we used a relatively low number of animals as well as SNPs. Although we found several significant QTLs, the finding that some QTLs appear when SERT^-/- ^and SERT^+/+ ^are combined (data not shown), suggests that the inclusion of additional animals will reveal more QTLs. Secondly, we standardized our screen as good as possible and noted all environmental factors (e.g. day of week, temperature, humidity, etc.). Yet, these environmental factors were not included in the analysis, leaving the possibility that QTLs are partly determined by environmental influences. Given that the overall QTL analysis revealed clear QTLs for coat color and genotype, but no other QTLs, suggest that the role of environmental factors is rather limited in this study. Finally, we measured very basic behaviors that may not be directly related to human behaviors or personality features. We consider this experiment as a primary screen providing targets for more in-depth dissections of QTLs and their behavioral correlates.

## Conclusion

We have revealed novel QTLs that modify the behavioral phenotypes of SERT deficiency in rats. Although the specific genetic modifiers remain to be identified, exploration of the QTL regions has lead to candidate genes that plausibly interact with SERT. Therefore, not only a fine-tuning of the QTL regions by the inclusion of additional markers and/or marker-assisted breeding and differential crossing-overs among F2 animals will bring us closer to the genetic modifiers, also a reverse genetics approach [43-45] in which the candidate genes are studied in more detail in SERT^-/- ^rodents might be highly valuable. We anticipate that the understanding of gene × gene interactions in SERT^-/- ^rodents could contribute to the identification of 5-HTTLPR genetic modifiers in humans, and thereby reveal molecular pathways that in interaction with the 5-HTTLPR either increase disease vulnerability, or provide disease resilience. This may further assist individualized therapies, given the broad spectrum of psychiatric conditions that are affected by the 5-HTTLPR.

## Methods

All experiments were conducted with the approval of the animal ethics committee of the Royal Academy of Sciences, The Netherlands. Experiments were designed to minimize the number of required animals and their suffering.

### Animals

To determine the parental phenotypes seven male and five female WI SERT^-/-^, eight male and four female WI SERT^+/+^, seven male and 10 female BN rats were tested in the experimental setups. The BN rats were purchased from Harlan at the age of 21 days. The SERT^-/- ^and SERT^+/+ ^rats, which are generated by ENU-induced mutagenesis [45] and were outcrossed for eight generations, were generated by SERT^+/- ^and SERT^+/- ^crossings in our laboratory. The animals were ear clipped at the age of two weeks under isoflurane anaesthesia, and genotyped as described previously (forward primer: TCACAAAGCACTGAGACCAG; reverse primer: AACCTGCCAAGAGAGAGTTG [45,46]). At the age of three weeks the animals were weaned and housed two or three animals per cage in standard Macrolon^® ^type III cages with a shelter and a piece of wood, in temperature controlled rooms (21°C ± 2) and relative humidity of 60 ± 15% with standard 12 hour light/dark cycle (lights off at 6 p.m.) and food and water *ad libitum*. At the age of 62-80 days the male rats were tested in the Phenotyper (Noldus Information Technology, Wageningen, The Netherlands) for three and 10 days, as well as cocaine-induced locomotor activity as described below. Data of the 10 day Phenotyper measurement are not presented. In addition, female rats 100-120 days of age were used to determine strain/genotype levels of abdominal and subcutanenous WAT (see below).

To generate the animals that were used in the genetic modifier screen, five female outbred SERT^-/- ^WI rats were crossed with two male wild type inbred BN rats. The resulting progeny (F1) was heterozygous for the SERT knockout allele (SERT^+/-^) and heterozygous for BN/WI polymorphisms. Brother-sister matings were conducted among F1 animals to get an F2 generation consisting of SERT^-/-^, SERT^+/- ^and SERT^+/+ ^rats that are homozygous or heterozygous for BN and WI alleles (Additional file 1 Figure S1). The F2 generation was ear clipped at the age of two weeks under isoflurane anesthesia, and 74 male SERT^-/-^, 27 male SERT^+/+^, 38 female SERT^-/- ^and 27 female SERT^+/+ ^animals (Additional file 1 table S1) were selected by SERT genotyping. The animals were weaned at the age of three weeks, and housed two or three per cage in standard Macrolon^® ^type III cages with a shelter and a piece of wood, in temperature controlled rooms (21°C ± 2) and relative humidity of 60 ± 15% with standard 12 hour light/dark cycle (lights off at 6 p.m.) and food and water *ad libitum*. The animals were weighted and handled according to the scheme indicated in Additional file 1 table S9. 1 Week after behavioral testing, the animals were sacrificed using CO_2_/O_2_, and ear cuts were taken for SNP genotyping and reconfirmation of genotypes. Also after collection of the WAT ear cuts were taken.

### DNA isolation and SERT genotyping

Ear cuts from the animals were collected in 96 deep-well plates. To each well 400 μL lysis buffer, containing 1 M Tris HCL with pH 8, 5 M NaCl, 10%SDS, 0,5 mM EDTA, and 100 mg/ml of freshly Proteinase K (from -20°C) was added, the deep-well plate was sealed with heat seal and incubated overnight at 55°C. After this the samples were incubated at 80°C for 15 minutes, cooled down to room temperature and to each well 300 μL isopropanol was added. After sealing, the mix was inverted 10 times and centrifuged at 3000 rcf for 40 minutes at 4°C. Then the block was gently inverted to remove supernatant and the pellets were washed by adding 400 μL 70% ethanol and centrifuged at 3000 rcf for 15 minutes at 4°C. Again supernatant was removed by inverting and the pellets were air dried after which they were dissolved in 500 μL H_2_O and stored at -20°C.

### SNP genotyping

The generation of the rat SNP panel consisting of 324 SNPs that are homozygously polymorphic between WI and BN and are evenly distributed over the genome is described elsewhere [25]. This panel has been well validated; heterozygotes could be easily discerned from WI and BN homozygous genotypes. Assay plates for genotyping by KASPar technology (Kbiosciences) containing freeze-dried primers sets of the SNPs (two allele-specific oligonucleotides of about 40 nt in length and 1 common oligonucleotide of about 20 nt in length) stored at -20°C were used. Each SNP was typed in a total volume of 4 μl in the following reaction mixture: 6 ng DNA, 22 mM MgCl_2_, KTaq, 1 μl 4× reaction mix, 2 μl pre-plated assay mix according to the manufacturer's guidelines (Kbiosciences). Amplification was performed in Applied Biosystems GeneAmp 9700 thermocyclers running the following program: 94°C - 15' then 20 cycles of 94°C-10", 57°C-5" and 72°C-10", followed by 18 cycles of 94°C-10", 57°C-20" and 72°C-40". Fluorescence scanning of the reactions was done in a BMG labtech Pherastar scanner and the results were interpreted by the KlusterCaller 1.1 software (KBiosciences). All SNPs for a single individual were amplified in a single 384 well plate and afterwards all raw data for each locus was regrouped for all samples by a custom Perl script before interpretation by KlusterCaller.

### Behavioral screen

The behavioral screen was performed in an automated homecage behavior observation system (Rat Phenotyper^®^, developed by Noldus Information Technology, Wageningen, The Netherlands). The cages (45 × 45 × 45 cm), made of transparent Perspex walls and a black floor, were equipped with an empty feeding station and two holes for drinking bottles. Each cage had a top unit containing a built-in digital infrared-sensitive video camera, infrared lighting sources, and hardware needed for video tracking (Ethovision 3.0). Ethovision collects data at a rate of 5 samples per second. Four Phenotypers were connected to a single PC.

With 16 Phenotypers up to 16 male rats were screened simultaneously. Based on previous analyses of Wistar SERT^-/- ^and SERT^+/+ ^behavior in the Phenotyper, a standardized test covering 13 days plus a comeback day was designed (Additional file 1 table S9). All animals were tested at the age of 62-80 days. 4 hours before the start of the Phenotyper experiments the animals were transported to the Phenotyper test room. At 5 p.m., one hour before the lights were turned off, the animals were placed in the Phenotyper cages and housed in the cages during 3 days under a 12 hr day/light cycle, and thereafter during 10 days in continuous darkness. Following the 13 days of Phenotyper testing the animals were transported back to the experimental room in which they were previously housed, and handled once a week thereafter, on fixed days of age. Four weeks after the end of the Phenotyper screen, the rats were brought back to the Phenotyper test room, allowed to habituate to the room for two hrs and tested in the Phenotyper cages at 11.00 a.m. without sawdust, food and water. Subsequently they were tested for their locomotor response to cocaine according to the following scheme: 0-30 min: habituation; 30-60 min: i.p. saline injection (1 ml/kg); 60-80 min: i.p. saline injection; 80-170 min: i.p. cocaine injection at 20 mg/kg. Overall, environmental factors such as room temperature, humidity, day of experiment, cage enrichment, cage cleaning, and sibship were as much as possible controlled and noted in a diary.

### WAT collection

During the mid-light phase, 100-120-days old female rats were weighted and decapitated under isoflurane anesthesia. Abdominal WAT (mesenteric, perinephric and ovarian) and subcutaneous WAT were dissected, cleaned and weighted.

### Drugs

Cocaine was purchased from O.P.G. (Utrecht, The Netherlands), freshly dissolved in saline (20 mg/ml) 1 hour before the experiment and administered in a volume of 1 ml/kg.

### Data analysis

Behavioral performance in the Phenotyper was analysed using Ethovision 3.1 software (Noldus Information Technology, Wageningen, The Netherlands). The parameters are: 1] Phenotyper (time in shelter, time in centre, total distance moved, time spent on immobility, time spent on mobility (15-60% change in body position between subsequent data samples), time spent on strong mobility (>60% change in body position between subsequent data samples); 2] Cocaine-induced locomotor activity (time spent in centre, distance moved 0-10 min, distance moved 10-20 min, distance moved 30-120 min); 3] WAT (abdominal and subcutaneous WAT). Parameters are also indicated in Additional file 1 table S10. Abdominal and subcutaneous WAT weight was normalized by body weight, and expressed as grams of WAT/100 grams of body weight [23]. Because Kolmogorov-Smirnov and Shapiro-Wilk normality tests revealed a normal distribution of data, behavioral and WAT parameters of the parental strains were analysed using ANOVA. Subsequent postoc tests were carried out with Bonferroni correction. A log10 transformation was used to obtain a normal distribution of the F2 behavioral and WAT data, which were subsequently analysed using Student's T-test. Correlations between phenotypes were calculated using Pearson's product-moment correlation. Due to external noise that influences the tracking signal during long trackings ~5% data points of the 10-day continuous dark phase were missing. Therefore, we did not include the 10-day continuous dark phase in the analysis. Although the same animals were used for the 3-day Phenotyper and cocaine measurements, some animals were not included in the 3-day Phenotyper assessments due to loss of data points resulting from a hard-disk crash. All analyses were performed using SPSS 16.0 software package. Statistical significant effects were set at P < 0.05 (before Bonferroni correction), and were adjusted using the Bonferroni method (0.05/number of comparisons).

### Composite interval mapping (CIM)

In order to detect mQTL regions we used a composite interval mapping approach [47] using the Windows QTL Cartographer package v2.5 [48]. The populations were split based on their SERT genotype to reveal SERT^-/- ^specific QTLs. Several thousand permutation runs (see table 1) were performed to determine the genome wide significance levels (GWSL) for each trait and the resulting thresholds were used to determine significant peaks.

### Candidate gene search

To explore candidate genes within QTL regions we used the BioMart tool of the Ensembl Genome Browser (see http://www.ensembl.org). We used the Ensembl 54 database for Rattus Norvegicus genes RGSC3.4, and used genomic regions around the associated markers and used the adjacent non-significant makers as limits. Using the Rat Genome Database http://www.rgd.mcw.edu we also explored whether SERT^-/-^-linked QTLs that were identified in this study match known QTLs.

## List of abbreviations

**5-HTTLPR**: Serotonin Transporter-Linked Polymorphic Region; **5-HTT**: Serotonin Transporter (human); **BDNF: **Brain-derived Neurotrophic Factor; **BN**: Brown Norway; **Chr**: Chromosome; **CIM **Composite Interval mapping; **cM**: Centimorgan; **COMT: **Catehcol-O-methyltransferase; **FHH**: Fawn Hooded Hypertensive; **GWSL: **Genome wide significance level; **LOD**: Logarithm Of Odds; **bp: **basepairs; **SERT**^-/-^: Homozygous serotonin transporter knockout rat; **SERT**^+/-^: Heterozygous serotonin transporter knockout rat; **SERT**^+/+^: Wild-type rat; **Rcf: **Relative centrifugal force; **RGD **Rat Genome Database; **RI**: Recombinant Inbred; **QTL: **Quantitative Trait Loci; **SHR**: spontaneously hypertensive; **SNP**: Single nucleotide polymorphism; **SS**: Dahl Salt Sensitive; **WAT**: White adipose tissue; **WI: **Wistar.

## Authors' contributions

JH participated in the design of the study, bred the animals, acquired the behavioral and body fat data, carried out the statistical analyses, conducted the *in silico *mapping of genetic modifiers, and wrote the manuscript; IJN performed the QTL analysis; SK assisted in animal breeding, behavioral screening and conducted the SNP genotyping; EC participated in the design and coordination of the study and helped to draft the manuscript. All authors read and approved the final manuscript.

## Supplementary Material

Additional file 1 **figures and tables**. Table S1: F2 animals tested per test. Tables S2-4: Traits of F0 SERT^+/+^, SERT^-/- ^and BN rats. Tables S5-7: Traits of F2 SERT^+/+ ^and SERT^-/- ^rats. Table S8: Candidate genes within QTL regions. Table S9: Phenotyping scheme. Table S10: Traits measured in the F2 animals. Figure S1: Breeding scheme Click here for file
