# Supplementary material for: Identification of genetic modifiers of behavioral phenotypes in serotonin transporter knockout rats
Source: BMC Genet. 2010 May 7;11:37. doi: 10.1186/1471-2156-11-37 (PMC2874760; doi:10.1186/1471-2156-11-37)
Supplement: Additional file 1 — figures and tables. Table S1: F2 animals tested per test. Tables S2-4: Traits of F0 SERT+/+, SERT-/- and BN rats. Tables S5-7: Traits of F2 SERT+/+ and SERT-/- rats. Table S8: Candidate genes within QTL regions. Table S9: Phenotyping scheme. Table S10: Traits measured in the F2 animals. Figure S1: Breeding scheme [file 1471-2156-11-37-S1.DOC]

**Supplementary figures and tables**

**Identification of genetic modifiers of behavioral phenotypes in serotonin transporter knockout rats**

Judith Homberg, Ies Nijman, Sylvia Kuijpers, Edwin Cuppen

Contents:

Table S1: F2 animals tested per test

Tables S2-4: Traits of F0 SERT^+/+^, SERT^-/-^ and BN rats

Tables S5-7: Traits of F2 SERT^+/+^ and SERT^-/-^ rats

Table S8: Candidate genes within QTL regions

Table S9: Phenotyping scheme

Table S10: Traits measured in the F2 animals

Figure S1: Breeding scheme

**Table S**1: F2 animals tested per test

| **Genotype** | **Gender** | **DL** | **Cocaine** | **WAT** |
| --- | --- | --- | --- | --- |
| SERT^-/-^ | female | - | - | 38 |
| SERT^+/+^ | female | - | - | 27 |
| SERT^-/-^ | male | 61 | 74 | - |
| SERT^+/+^ | male | 21 | 27 | - |

**Table S2: P0 behavioural phenotypes in the Phenotyper**

| **Three days – D/L** | **SERT^+/+^** | **SERT^-/-^** | **BN** | **ANOVA** |
| --- | --- | --- | --- | --- |
| **T in Shelter (s)** | 209095 ± 2878 | 217898 ± 2766 | 221383 ± 2254^a^ | F(1,20) = 8.14, P=0.003****** |
| **T in centre (s)** | 770 ± 216 | 307 ± 57 | 328 ± 88 | F(1,20) = 3.58, P=0.048 |
| **Distance moved (cm)** | 93820 ± 3732 | 84093 ± 8704 | 73980 ± 4883 | F(1,20) = 4.44, P= 0.021 |
| **T immobility (s)** | 37323 ± 1943 | 45351 ± 2075* | 32294 ± 1534^b^ | F(1,20) = 12.51, P=0.001****** |
| **T mobility (s)** | 5104 ± 453 | 4467 ± 528 | 3506 ± 216 | F(1,20) = 1.96 |
| **T strong mobility (s)** | 53 ± 8 | 47 ± 8 | 209 ± 48^c^ | F(1,20) = 11.07, P=0.001****** |
| Male rats only  P-values are uncorrected, **significant effect after Bonferroni correction (significance level P < 0.0083)  Posthoc: * P< 0.0083; SERT^-/-^ compared to SERT^+/+^; ^a^P < 0.0083 BN compared to SERT^+/+^; ^b^ P < 0.0083 BN compared to SERT^-/-^; ^c^ P < 0.0083 BN compared to SERT^-/-^ and SERT^+/+^ | | | | |

**Table S3:** P0 cocaine-induced locomotor activity

| **Cocaine -20 mg/kg** | **SERT^+/+^** | **SERT^-/-^** | **BN** | **ANOVA** |
| --- | --- | --- | --- | --- |
| **T in Centre (s)** | 423 ± 116 | 467 ± 73 | 241 ± 81 | F(1,18) = 2.67 |
| **DM 0-10 min (cm)** | 1081 ± 345 | 3742 ± 685* | 1278 ± 307^b^ | F(1,18) = 10.23, p=0.001****** |
| **DM 10-20 min (cm)** | 3225 ± 890 | 4365 ± 314 | 1661 ± 264^b^ | F(1,18) = 8.32, p=0.003****** |
| **DM 30-120 min (cm)** | 11568 ±1203 | 22730 ± 2725* | 10761 ± 1166^b^ | F(1,18) = 14.28, p=0.001****** |
| Male rats only  P-values are uncorrected, **significant effect after Bonferroni correction (significance level P < 0.0125)  Posthoc: * P< 0.0125; SERT^-/-^ compared to SERT^+/+^; ^b^ P < 0.0125 BN compared to SERT^-/-^ | | | | |

**Table S4: P0 White Adipose Tissue (WAT)**

| **WAT/100 gram** | **SERT^+/+^** | **SERT^-/-^** | **BN** | **ANOVA** |
| --- | --- | --- | --- | --- |
| **Abdominal (gr)** | 1.65 ± 0.17 | 2.37 ± 0.17* | 1.07 ± 0.05^a,b^ | F(2,16) = 37.578, p<0.001****** |
| **Subcutaneous (gr)** | 0.51 ± 0.05 | 0.64 ± 0.09 | 0.22 ± 0.05 | F(2,16) = 22.510, p<0.001****** |
| Female rats only  P-values are uncorrected, **significant effect after Bonferroni correction (significance level P < 0.025)  Posthoc: *P< 0.025; SERT^-/-^ compared to SERT^+/+^; ^a^ P < 0.025 SERT^+/+^ compared to BN; ^b^P < 0.025 SERT^-/-^ compared to BN. | | | | |

**Table S5: F2 behavioural phenotypes in the Phenotyper**

| **Phenotyper** | **SERT^+/+^** | **SERT^-/-^** | **Student T- test** |
| --- | --- | --- | --- |
| **T in Shelter (s)** | 207737 ± 3505 | 214491 ± 2058 | T(1,80) = 0.230 |
| **T in centre (s)** | 292 ± 35 | 307 ± 37 | T(1,80) = 0.307 |
| **Distance moved (cm)** | 68542 ± 3377 | 67894 ± 2726 | T(1,80) = 0.932 |
| **T immobility** | 42592 ± 2428 | 42462 ± 2174 | T(1,80) = 0.687 |
| **T mobility** | 2979 ± 256 | 2814 ± 159 | T(1,80) = 0.879 |
| **T strong mobility** | 44 ± 7 | 39 ± 8 | T(1,80) = 0.292 |
| Male rats only | | | |

**Table S6:** F2 cocaine-induced locomotor activity

| **Cocaine** | **SERT^+/+^** | **SERT^-/-^** | **Student T- test** |
| --- | --- | --- | --- |
| **T in Centre (s)** | 184 ± 44 | 77 ± 12 | T(1,99) = 2.818, P= 0.006** |
| **DM 0-10 min (cm)** | 2410 ± 352 | 2301 ± 252 | T(1,99) = 0.301 |
| **DM 10-20 min (cm)** | 2445± 303 | 2381 ±191 | T(1,99) = 0.589 |
| **DM 30-120 min (cm)** | 6918 ± 783 | 8976 ± 640 | T(1,99 ) = 0.441, P= 0.021 |
| Male rats only  P-values are uncorrected, **significant effect after Bonferroni correction (significance level P < 0.0125) | | | |

**Table S7: F2 White Adipose Tissue (WAT)**

| **WAT/100 gram-F** | **SERT^+/+^** | **SERT^-/-^** | **Student T- test** |
| --- | --- | --- | --- |
| **Abdominal (gr)** | 1.21 ± 0.05 | 1.66 ± 0.061 | T(1,63) = 6.919, P= 0.0001** |
| **Subcutaneous (gr)** | 0.32 ± 0.01 | 0.43 ± 0.01 | T(1,63) = 5.221, P= 0.0001** |
| Female rats only  P-values are uncorrected, **significant effect after Bonferroni correction (significance level P < 0.025) | | | |

**Table S8**

Candidate genes within significant QTL regions as indicated in table 1. In the left column also the WI QTL submitted to RGD ([http://www.rgd.mcw.edu](http://www.rgd.mcw.edu/)) is indicated. Gene starts and ends are depicted in bp.

| **Parameters - SERT-/-** | **Ensembl Gene ID** | **Chr.** | **Gene Start** | **Gene End** | **Gene Name** | **Description** |
| --- | --- | --- | --- | --- | --- | --- |
| Time in shelter | [ENSRNOG00000043209](http://www.ensembl.org/Rattus_norvegicus/Gene/Summary?db=core;g=ENSRNOG00000043209) | [1](http://www.ensembl.org/Rattus_norvegicus/mapview?chr=1) | [44921171](http://www.ensembl.org/Rattus_norvegicus/contigview?chr=1&vc_start=44921171&vc_end=44921721) | [44921721](http://www.ensembl.org/Rattus_norvegicus/contigview?chr=1&vc_start=44921171&vc_end=44921721) | [XM_001059887.1](http://www.ensembl.org/Rattus_norvegicus/geneview?gene=XM_001059887.1) |  |
|  | ENSRNOG00000017174 | 1 | 44922562 | 45027506 | QKI_RAT | Protein quaking (RqkI) |
|  | [ENSRNOG00000017363](http://www.ensembl.org/Rattus_norvegicus/Gene/Summary?db=core;g=ENSRNOG00000017363) | [1](http://www.ensembl.org/Rattus_norvegicus/mapview?chr=1) | [45126368](http://www.ensembl.org/Rattus_norvegicus/contigview?chr=1&vc_start=45126368&vc_end=45126687) | [45126687](http://www.ensembl.org/Rattus_norvegicus/contigview?chr=1&vc_start=45126368&vc_end=45126687) |  |  |
|  | ENSRNOG00000017367 | 1 | 45590426 | 45593497 | NP_001099678.1 | poly(A) binding protein, cytoplasmic 3 |
|  | [ENSRNOG00000012974](http://www.ensembl.org/Rattus_norvegicus/Gene/Summary?db=core;g=ENSRNOG00000012974) | [1](http://www.ensembl.org/Rattus_norvegicus/mapview?chr=1) | [47109216](http://www.ensembl.org/Rattus_norvegicus/contigview?chr=1&vc_start=47109216&vc_end=47109808) | [47109808](http://www.ensembl.org/Rattus_norvegicus/contigview?chr=1&vc_start=47109216&vc_end=47109808) | [XR_006046.1](http://www.ensembl.org/Rattus_norvegicus/geneview?gene=XR_006046.1) |  |
|  | [ENSRNOG00000013190](http://www.ensembl.org/Rattus_norvegicus/Gene/Summary?db=core;g=ENSRNOG00000013190) | [1](http://www.ensembl.org/Rattus_norvegicus/mapview?chr=1) | [47227491](http://www.ensembl.org/Rattus_norvegicus/contigview?chr=1&vc_start=47227491&vc_end=47244660) | [47244660](http://www.ensembl.org/Rattus_norvegicus/contigview?chr=1&vc_start=47227491&vc_end=47244660) | [NP_001099680.1](http://www.ensembl.org/Rattus_norvegicus/geneview?gene=NP_001099680.1) | ribonuclease T2 |
|  | [ENSRNOG00000013194](http://www.ensembl.org/Rattus_norvegicus/Gene/Summary?db=core;g=ENSRNOG00000013194) | [1](http://www.ensembl.org/Rattus_norvegicus/mapview?chr=1) | [47479545](http://www.ensembl.org/Rattus_norvegicus/contigview?chr=1&vc_start=47479545&vc_end=47557576) | [47557576](http://www.ensembl.org/Rattus_norvegicus/contigview?chr=1&vc_start=47479545&vc_end=47557576) | [Q9Z1I2_RAT](http://www.ensembl.org/Rattus_norvegicus/geneview?gene=Q9Z1I2_RAT) | p90 S6 kinase Fragment |
|  | [ENSRNOG00000013374](http://www.ensembl.org/Rattus_norvegicus/Gene/Summary?db=core;g=ENSRNOG00000013374) | [1](http://www.ensembl.org/Rattus_norvegicus/mapview?chr=1) | [47592824](http://www.ensembl.org/Rattus_norvegicus/contigview?chr=1&vc_start=47592824&vc_end=47616165) | [47616165](http://www.ensembl.org/Rattus_norvegicus/contigview?chr=1&vc_start=47592824&vc_end=47616165) | [NP_001100931.1](http://www.ensembl.org/Rattus_norvegicus/geneview?gene=NP_001100931.1) | t-complex protein 10b |
|  | [ENSRNOG00000043398](http://www.ensembl.org/Rattus_norvegicus/Gene/Summary?db=core;g=ENSRNOG00000043398) | [1](http://www.ensembl.org/Rattus_norvegicus/mapview?chr=1) | [47781091](http://www.ensembl.org/Rattus_norvegicus/contigview?chr=1&vc_start=47781091&vc_end=47783114) | [47783114](http://www.ensembl.org/Rattus_norvegicus/contigview?chr=1&vc_start=47781091&vc_end=47783114) | [LOC685019](http://www.ensembl.org/Rattus_norvegicus/geneview?gene=LOC685019) |  |
|  | [ENSRNOG00000034080](http://www.ensembl.org/Rattus_norvegicus/Gene/Summary?db=core;g=ENSRNOG00000034080) | [1](http://www.ensembl.org/Rattus_norvegicus/mapview?chr=1) | [47909277](http://www.ensembl.org/Rattus_norvegicus/contigview?chr=1&vc_start=47909277&vc_end=47910725) | [47910725](http://www.ensembl.org/Rattus_norvegicus/contigview?chr=1&vc_start=47909277&vc_end=47910725) |  |  |
|  | [ENSRNOG00000042841](http://www.ensembl.org/Rattus_norvegicus/Gene/Summary?db=core;g=ENSRNOG00000042841) | [1](http://www.ensembl.org/Rattus_norvegicus/mapview?chr=1) | [48397272](http://www.ensembl.org/Rattus_norvegicus/contigview?chr=1&vc_start=48397272&vc_end=48398720) | [48398720](http://www.ensembl.org/Rattus_norvegicus/contigview?chr=1&vc_start=48397272&vc_end=48398720) |  |  |
|  | [ENSRNOG00000042364](http://www.ensembl.org/Rattus_norvegicus/Gene/Summary?db=core;g=ENSRNOG00000042364) | [1](http://www.ensembl.org/Rattus_norvegicus/mapview?chr=1) | [48592014](http://www.ensembl.org/Rattus_norvegicus/contigview?chr=1&vc_start=48592014&vc_end=48593462) | [48593462](http://www.ensembl.org/Rattus_norvegicus/contigview?chr=1&vc_start=48592014&vc_end=48593462) | [IPI00766615.1](http://www.ensembl.org/Rattus_norvegicus/geneview?gene=IPI00766615.1) |  |
|  | [ENSRNOG00000014157](http://www.ensembl.org/Rattus_norvegicus/Gene/Summary?db=core;g=ENSRNOG00000014157) | [1](http://www.ensembl.org/Rattus_norvegicus/mapview?chr=1) | [48715390](http://www.ensembl.org/Rattus_norvegicus/contigview?chr=1&vc_start=48715390&vc_end=48718167) | [48718167](http://www.ensembl.org/Rattus_norvegicus/contigview?chr=1&vc_start=48715390&vc_end=48718167) | [LOC365114](http://www.ensembl.org/Rattus_norvegicus/geneview?gene=LOC365114) |  |
|  | [ENSRNOG00000023753](http://www.ensembl.org/Rattus_norvegicus/Gene/Summary?db=core;g=ENSRNOG00000023753) | [1](http://www.ensembl.org/Rattus_norvegicus/mapview?chr=1) | [48827689](http://www.ensembl.org/Rattus_norvegicus/contigview?chr=1&vc_start=48827689&vc_end=48913175) | [48913175](http://www.ensembl.org/Rattus_norvegicus/contigview?chr=1&vc_start=48827689&vc_end=48913175) | [AFAD_RAT](http://www.ensembl.org/Rattus_norvegicus/geneview?gene=AFAD_RAT) | Afadin (Protein Af-6) |
|  | [ENSRNOG00000039226](http://www.ensembl.org/Rattus_norvegicus/Gene/Summary?db=core;g=ENSRNOG00000039226) | [1](http://www.ensembl.org/Rattus_norvegicus/mapview?chr=1) | [48964276](http://www.ensembl.org/Rattus_norvegicus/contigview?chr=1&vc_start=48964276&vc_end=48965190) | [48965190](http://www.ensembl.org/Rattus_norvegicus/contigview?chr=1&vc_start=48964276&vc_end=48965190) | [XR_006355.1](http://www.ensembl.org/Rattus_norvegicus/geneview?gene=XR_006355.1) |  |
|  | [ENSRNOG00000035828](http://www.ensembl.org/Rattus_norvegicus/Gene/Summary?db=core;g=ENSRNOG00000035828) | [1](http://www.ensembl.org/Rattus_norvegicus/mapview?chr=1) | [48991177](http://www.ensembl.org/Rattus_norvegicus/contigview?chr=1&vc_start=48991177&vc_end=48991288) | [48991288](http://www.ensembl.org/Rattus_norvegicus/contigview?chr=1&vc_start=48991177&vc_end=48991288) | [Y_RNA](http://www.ensembl.org/Rattus_norvegicus/geneview?gene=Y_RNA) | Y RNA [Source: RFAM;Acc:RF00019] |
|  | [ENSRNOG00000033713](http://www.ensembl.org/Rattus_norvegicus/Gene/Summary?db=core;g=ENSRNOG00000033713) | [1](http://www.ensembl.org/Rattus_norvegicus/mapview?chr=1) | [49024185](http://www.ensembl.org/Rattus_norvegicus/contigview?chr=1&vc_start=49024185&vc_end=49033584) | [49033584](http://www.ensembl.org/Rattus_norvegicus/contigview?chr=1&vc_start=49024185&vc_end=49033584) | [IPI00561433.2](http://www.ensembl.org/Rattus_norvegicus/geneview?gene=IPI00561433.2) |  |
|  | [ENSRNOG00000026000](http://www.ensembl.org/Rattus_norvegicus/Gene/Summary?db=core;g=ENSRNOG00000026000) | [1](http://www.ensembl.org/Rattus_norvegicus/mapview?chr=1) | [49184902](http://www.ensembl.org/Rattus_norvegicus/contigview?chr=1&vc_start=49184902&vc_end=49214197) | [49214197](http://www.ensembl.org/Rattus_norvegicus/contigview?chr=1&vc_start=49184902&vc_end=49214197) | [NP_001092933.1](http://www.ensembl.org/Rattus_norvegicus/geneview?gene=NP_001092933.1) | vomeronasal 2 receptor 7 |
|  | [ENSRNOG00000031954](http://www.ensembl.org/Rattus_norvegicus/Gene/Summary?db=core;g=ENSRNOG00000031954) | [1](http://www.ensembl.org/Rattus_norvegicus/mapview?chr=1) | [49591810](http://www.ensembl.org/Rattus_norvegicus/contigview?chr=1&vc_start=49591810&vc_end=49631453) | [49631453](http://www.ensembl.org/Rattus_norvegicus/contigview?chr=1&vc_start=49591810&vc_end=49631453) | [NP_001092934.1](http://www.ensembl.org/Rattus_norvegicus/geneview?gene=NP_001092934.1) | vomeronasal 2 receptor 8 |
|  | [ENSRNOG00000013671](http://www.ensembl.org/Rattus_norvegicus/Gene/Summary?db=core;g=ENSRNOG00000013671) | [1](http://www.ensembl.org/Rattus_norvegicus/mapview?chr=1) | [49771554](http://www.ensembl.org/Rattus_norvegicus/contigview?chr=1&vc_start=49771554&vc_end=49785337) | [49785337](http://www.ensembl.org/Rattus_norvegicus/contigview?chr=1&vc_start=49771554&vc_end=49785337) | [NP_001092935.1](http://www.ensembl.org/Rattus_norvegicus/geneview?gene=NP_001092935.1) | vomeronasal 2 receptor 9 |
|  | [ENSRNOG00000023595](http://www.ensembl.org/Rattus_norvegicus/Gene/Summary?db=core;g=ENSRNOG00000023595) | [1](http://www.ensembl.org/Rattus_norvegicus/mapview?chr=1) | [49834280](http://www.ensembl.org/Rattus_norvegicus/contigview?chr=1&vc_start=49834280&vc_end=49835315) | [49835315](http://www.ensembl.org/Rattus_norvegicus/contigview?chr=1&vc_start=49834280&vc_end=49835315) |  |  |
|  | [ENSRNOG00000042518](http://www.ensembl.org/Rattus_norvegicus/Gene/Summary?db=core;g=ENSRNOG00000042518) | [1](http://www.ensembl.org/Rattus_norvegicus/mapview?chr=1) | [49934843](http://www.ensembl.org/Rattus_norvegicus/contigview?chr=1&vc_start=49934843&vc_end=49947137) | [49947137](http://www.ensembl.org/Rattus_norvegicus/contigview?chr=1&vc_start=49934843&vc_end=49947137) |  |  |
|  | [ENSRNOG00000031160](http://www.ensembl.org/Rattus_norvegicus/Gene/Summary?db=core;g=ENSRNOG00000031160) | [1](http://www.ensembl.org/Rattus_norvegicus/mapview?chr=1) | [50141775](http://www.ensembl.org/Rattus_norvegicus/contigview?chr=1&vc_start=50141775&vc_end=50143238) | [50143238](http://www.ensembl.org/Rattus_norvegicus/contigview?chr=1&vc_start=50141775&vc_end=50143238) | [RGD1564848](http://www.ensembl.org/Rattus_norvegicus/geneview?gene=RGD1564848) |  |
|  | [ENSRNOG00000039182](http://www.ensembl.org/Rattus_norvegicus/Gene/Summary?db=core;g=ENSRNOG00000039182) | [1](http://www.ensembl.org/Rattus_norvegicus/mapview?chr=1) | [50431647](http://www.ensembl.org/Rattus_norvegicus/contigview?chr=1&vc_start=50431647&vc_end=50432489) | [50432489](http://www.ensembl.org/Rattus_norvegicus/contigview?chr=1&vc_start=50431647&vc_end=50432489) | [XR_006355.1](http://www.ensembl.org/Rattus_norvegicus/geneview?gene=XR_006355.1) |  |
|  | [ENSRNOG00000042088](http://www.ensembl.org/Rattus_norvegicus/Gene/Summary?db=core;g=ENSRNOG00000042088) | [1](http://www.ensembl.org/Rattus_norvegicus/mapview?chr=1) | [50650733](http://www.ensembl.org/Rattus_norvegicus/contigview?chr=1&vc_start=50650733&vc_end=50652262) | [50652262](http://www.ensembl.org/Rattus_norvegicus/contigview?chr=1&vc_start=50650733&vc_end=50652262) | [RGD1563626](http://www.ensembl.org/Rattus_norvegicus/geneview?gene=RGD1563626) |  |
|  | [ENSRNOG00000039180](http://www.ensembl.org/Rattus_norvegicus/Gene/Summary?db=core;g=ENSRNOG00000039180) | [1](http://www.ensembl.org/Rattus_norvegicus/mapview?chr=1) | [50927926](http://www.ensembl.org/Rattus_norvegicus/contigview?chr=1&vc_start=50927926&vc_end=50943110) | [50943110](http://www.ensembl.org/Rattus_norvegicus/contigview?chr=1&vc_start=50927926&vc_end=50943110) | [IPI00568890.2](http://www.ensembl.org/Rattus_norvegicus/geneview?gene=IPI00568890.2) |  |
|  | [ENSRNOG00000023225](http://www.ensembl.org/Rattus_norvegicus/Gene/Summary?db=core;g=ENSRNOG00000023225) | [1](http://www.ensembl.org/Rattus_norvegicus/mapview?chr=1) | [50994229](http://www.ensembl.org/Rattus_norvegicus/contigview?chr=1&vc_start=50994229&vc_end=50995758) | [50995758](http://www.ensembl.org/Rattus_norvegicus/contigview?chr=1&vc_start=50994229&vc_end=50995758) | [RGD1565231](http://www.ensembl.org/Rattus_norvegicus/geneview?gene=RGD1565231) |  |
|  | [ENSRNOG00000032704](http://www.ensembl.org/Rattus_norvegicus/Gene/Summary?db=core;g=ENSRNOG00000032704) | [1](http://www.ensembl.org/Rattus_norvegicus/mapview?chr=1) | [51326410](http://www.ensembl.org/Rattus_norvegicus/contigview?chr=1&vc_start=51326410&vc_end=51327938) | [51327938](http://www.ensembl.org/Rattus_norvegicus/contigview?chr=1&vc_start=51326410&vc_end=51327938) | [XR_006315.1](http://www.ensembl.org/Rattus_norvegicus/geneview?gene=XR_006315.1) |  |
|  | [ENSRNOG00000039177](http://www.ensembl.org/Rattus_norvegicus/Gene/Summary?db=core;g=ENSRNOG00000039177) | [1](http://www.ensembl.org/Rattus_norvegicus/mapview?chr=1) | [51378345](http://www.ensembl.org/Rattus_norvegicus/contigview?chr=1&vc_start=51378345&vc_end=51379930) | [51379930](http://www.ensembl.org/Rattus_norvegicus/contigview?chr=1&vc_start=51378345&vc_end=51379930) |  |  |
|  | [ENSRNOG00000033340](http://www.ensembl.org/Rattus_norvegicus/Gene/Summary?db=core;g=ENSRNOG00000033340) | [1](http://www.ensembl.org/Rattus_norvegicus/mapview?chr=1) | [51791819](http://www.ensembl.org/Rattus_norvegicus/contigview?chr=1&vc_start=51791819&vc_end=51793348) | [51793348](http://www.ensembl.org/Rattus_norvegicus/contigview?chr=1&vc_start=51791819&vc_end=51793348) | [RGD1565231](http://www.ensembl.org/Rattus_norvegicus/geneview?gene=RGD1565231) |  |
|  | [ENSRNOG00000042315](http://www.ensembl.org/Rattus_norvegicus/Gene/Summary?db=core;g=ENSRNOG00000042315) | [1](http://www.ensembl.org/Rattus_norvegicus/mapview?chr=1) | [52322287](http://www.ensembl.org/Rattus_norvegicus/contigview?chr=1&vc_start=52322287&vc_end=52323729) | [52323729](http://www.ensembl.org/Rattus_norvegicus/contigview?chr=1&vc_start=52322287&vc_end=52323729) | [LOC689315](http://www.ensembl.org/Rattus_norvegicus/geneview?gene=LOC689315) |  |
|  | [ENSRNOG00000030145](http://www.ensembl.org/Rattus_norvegicus/Gene/Summary?db=core;g=ENSRNOG00000030145) | [1](http://www.ensembl.org/Rattus_norvegicus/mapview?chr=1) | [52337585](http://www.ensembl.org/Rattus_norvegicus/contigview?chr=1&vc_start=52337585&vc_end=52338883) | [52338883](http://www.ensembl.org/Rattus_norvegicus/contigview?chr=1&vc_start=52337585&vc_end=52338883) |  |  |
|  | [ENSRNOG00000042452](http://www.ensembl.org/Rattus_norvegicus/Gene/Summary?db=core;g=ENSRNOG00000042452) | [1](http://www.ensembl.org/Rattus_norvegicus/mapview?chr=1) | [52447152](http://www.ensembl.org/Rattus_norvegicus/contigview?chr=1&vc_start=52447152&vc_end=52448612) | [52448612](http://www.ensembl.org/Rattus_norvegicus/contigview?chr=1&vc_start=52447152&vc_end=52448612) |  |  |
|  | [ENSRNOG00000039169](http://www.ensembl.org/Rattus_norvegicus/Gene/Summary?db=core;g=ENSRNOG00000039169) | [1](http://www.ensembl.org/Rattus_norvegicus/mapview?chr=1) | [52475781](http://www.ensembl.org/Rattus_norvegicus/contigview?chr=1&vc_start=52475781&vc_end=52512123) | [52512123](http://www.ensembl.org/Rattus_norvegicus/contigview?chr=1&vc_start=52475781&vc_end=52512123) |  |  |
|  | [ENSRNOG00000033456](http://www.ensembl.org/Rattus_norvegicus/Gene/Summary?db=core;g=ENSRNOG00000033456) | [1](http://www.ensembl.org/Rattus_norvegicus/mapview?chr=1) | [52544218](http://www.ensembl.org/Rattus_norvegicus/contigview?chr=1&vc_start=52544218&vc_end=52545747) | [52545747](http://www.ensembl.org/Rattus_norvegicus/contigview?chr=1&vc_start=52544218&vc_end=52545747) | [RGD1561339](http://www.ensembl.org/Rattus_norvegicus/geneview?gene=RGD1561339) |  |
|  | [ENSRNOG00000014166](http://www.ensembl.org/Rattus_norvegicus/Gene/Summary?db=core;g=ENSRNOG00000014166) | [1](http://www.ensembl.org/Rattus_norvegicus/mapview?chr=1) | [53165792](http://www.ensembl.org/Rattus_norvegicus/contigview?chr=1&vc_start=53165792&vc_end=53295122) | [53295122](http://www.ensembl.org/Rattus_norvegicus/contigview?chr=1&vc_start=53165792&vc_end=53295122) | [NP_001099685.1](http://www.ensembl.org/Rattus_norvegicus/geneview?gene=NP_001099685.1) | SPARC related modular calcium binding 2 |
| **Parameters - SERT-/-** | **Ensembl Gene ID** | **Chr.** | **Gene Start** | **Gene End** | **Gene Name** | **Description** |
| Time in shelter | [ENSRNOG00000016327](http://www.ensembl.org/Rattus_norvegicus/Gene/Summary?db=core;g=ENSRNOG00000016327) | [1](http://www.ensembl.org/Rattus_norvegicus/mapview?chr=1) | [118832889](http://www.ensembl.org/Rattus_norvegicus/contigview?chr=1&vc_start=118832889&vc_end=118856087) | [118856087](http://www.ensembl.org/Rattus_norvegicus/contigview?chr=1&vc_start=118832889&vc_end=118856087) | [NP_001099811.1](http://www.ensembl.org/Rattus_norvegicus/geneview?gene=NP_001099811.1) | methylmalonyl CoA epimerase |
|  | [ENSRNOG00000016358](http://www.ensembl.org/Rattus_norvegicus/Gene/Summary?db=core;g=ENSRNOG00000016358) | [1](http://www.ensembl.org/Rattus_norvegicus/mapview?chr=1) | [118970876](http://www.ensembl.org/Rattus_norvegicus/contigview?chr=1&vc_start=118970876&vc_end=119156598) | [119156598](http://www.ensembl.org/Rattus_norvegicus/contigview?chr=1&vc_start=118970876&vc_end=119156598) | [APBA2_RAT](http://www.ensembl.org/Rattus_norvegicus/geneview?gene=APBA2_RAT) | Amyloid beta A4 precursor protein-binding family A member 2 |
| **Anxiety QTL 13** | [ENSRNOG00000037093](http://www.ensembl.org/Rattus_norvegicus/Gene/Summary?db=core;g=ENSRNOG00000037093) | [1](http://www.ensembl.org/Rattus_norvegicus/mapview?chr=1) | [119407238](http://www.ensembl.org/Rattus_norvegicus/contigview?chr=1&vc_start=119407238&vc_end=119412008) | [119412008](http://www.ensembl.org/Rattus_norvegicus/contigview?chr=1&vc_start=119407238&vc_end=119412008) | [IPI00776923.1](http://www.ensembl.org/Rattus_norvegicus/geneview?gene=IPI00776923.1) |  |
| **84023578-129023578** | [ENSRNOG00000016498](http://www.ensembl.org/Rattus_norvegicus/Gene/Summary?db=core;g=ENSRNOG00000016498) | [1](http://www.ensembl.org/Rattus_norvegicus/mapview?chr=1) | [119538208](http://www.ensembl.org/Rattus_norvegicus/contigview?chr=1&vc_start=119538208&vc_end=119540760) | [119540760](http://www.ensembl.org/Rattus_norvegicus/contigview?chr=1&vc_start=119538208&vc_end=119540760) |  |  |
|  | [ENSRNOG00000037092](http://www.ensembl.org/Rattus_norvegicus/Gene/Summary?db=core;g=ENSRNOG00000037092) | [1](http://www.ensembl.org/Rattus_norvegicus/mapview?chr=1) | [119865327](http://www.ensembl.org/Rattus_norvegicus/contigview?chr=1&vc_start=119865327&vc_end=119868408) | [119868408](http://www.ensembl.org/Rattus_norvegicus/contigview?chr=1&vc_start=119865327&vc_end=119868408) | [IPI00558831.2](http://www.ensembl.org/Rattus_norvegicus/geneview?gene=IPI00558831.2) |  |
|  | [ENSRNOG00000024460](http://www.ensembl.org/Rattus_norvegicus/Gene/Summary?db=core;g=ENSRNOG00000024460) | [1](http://www.ensembl.org/Rattus_norvegicus/mapview?chr=1) | [120050878](http://www.ensembl.org/Rattus_norvegicus/contigview?chr=1&vc_start=120050878&vc_end=120102655) | [120102655](http://www.ensembl.org/Rattus_norvegicus/contigview?chr=1&vc_start=120050878&vc_end=120102655) | [NP_001014042.1](http://www.ensembl.org/Rattus_norvegicus/geneview?gene=NP_001014042.1) | threonyl-tRNA synthetase-like 2 |
|  | [ENSRNOG00000011290](http://www.ensembl.org/Rattus_norvegicus/Gene/Summary?db=core;g=ENSRNOG00000011290) | [1](http://www.ensembl.org/Rattus_norvegicus/mapview?chr=1) | [120105142](http://www.ensembl.org/Rattus_norvegicus/contigview?chr=1&vc_start=120105142&vc_end=120116942) | [120116942](http://www.ensembl.org/Rattus_norvegicus/contigview?chr=1&vc_start=120105142&vc_end=120116942) | [NP_001099737.1](http://www.ensembl.org/Rattus_norvegicus/geneview?gene=NP_001099737.1) | TM2 domain containing 3 |
|  | [ENSRNOG00000011510](http://www.ensembl.org/Rattus_norvegicus/Gene/Summary?db=core;g=ENSRNOG00000011510) | [1](http://www.ensembl.org/Rattus_norvegicus/mapview?chr=1) | [120179468](http://www.ensembl.org/Rattus_norvegicus/contigview?chr=1&vc_start=120179468&vc_end=120180146) | [120180146](http://www.ensembl.org/Rattus_norvegicus/contigview?chr=1&vc_start=120179468&vc_end=120180146) |  |  |
|  | [ENSRNOG00000011526](http://www.ensembl.org/Rattus_norvegicus/Gene/Summary?db=core;g=ENSRNOG00000011526) | [1](http://www.ensembl.org/Rattus_norvegicus/mapview?chr=1) | [120324843](http://www.ensembl.org/Rattus_norvegicus/contigview?chr=1&vc_start=120324843&vc_end=120476913) | [120476913](http://www.ensembl.org/Rattus_norvegicus/contigview?chr=1&vc_start=120324843&vc_end=120476913) | [PCSK6_RAT](http://www.ensembl.org/Rattus_norvegicus/geneview?gene=PCSK6_RAT) | Proprotein convertase subtilisin/kexin type 6 Precursor |
|  | [ENSRNOG00000011932](http://www.ensembl.org/Rattus_norvegicus/Gene/Summary?db=core;g=ENSRNOG00000011932) | [1](http://www.ensembl.org/Rattus_norvegicus/mapview?chr=1) | [120490926](http://www.ensembl.org/Rattus_norvegicus/contigview?chr=1&vc_start=120490926&vc_end=120503971) | [120503971](http://www.ensembl.org/Rattus_norvegicus/contigview?chr=1&vc_start=120490926&vc_end=120503971) | [IPI00560470.1](http://www.ensembl.org/Rattus_norvegicus/geneview?gene=IPI00560470.1) |  |
|  | [ENSRNOG00000012576](http://www.ensembl.org/Rattus_norvegicus/Gene/Summary?db=core;g=ENSRNOG00000012576) | [1](http://www.ensembl.org/Rattus_norvegicus/mapview?chr=1) | [120509096](http://www.ensembl.org/Rattus_norvegicus/contigview?chr=1&vc_start=120509096&vc_end=120518861) | [120518861](http://www.ensembl.org/Rattus_norvegicus/contigview?chr=1&vc_start=120509096&vc_end=120518861) | [SELS_RAT](http://www.ensembl.org/Rattus_norvegicus/geneview?gene=SELS_RAT) | Selenoprotein S (SelS) |
|  | [ENSRNOG00000012698](http://www.ensembl.org/Rattus_norvegicus/Gene/Summary?db=core;g=ENSRNOG00000012698) | [1](http://www.ensembl.org/Rattus_norvegicus/mapview?chr=1) | [120538995](http://www.ensembl.org/Rattus_norvegicus/contigview?chr=1&vc_start=120538995&vc_end=120600102) | [120600102](http://www.ensembl.org/Rattus_norvegicus/contigview?chr=1&vc_start=120538995&vc_end=120600102) | [NP_001099738.1](http://www.ensembl.org/Rattus_norvegicus/geneview?gene=NP_001099738.1) | chondroitin sulfate synthase 1 |
|  | [ENSRNOG00000013811](http://www.ensembl.org/Rattus_norvegicus/Gene/Summary?db=core;g=ENSRNOG00000013811) | [1](http://www.ensembl.org/Rattus_norvegicus/mapview?chr=1) | [121163418](http://www.ensembl.org/Rattus_norvegicus/contigview?chr=1&vc_start=121163418&vc_end=121164899) | [121164899](http://www.ensembl.org/Rattus_norvegicus/contigview?chr=1&vc_start=121163418&vc_end=121164899) | [IPI00779150.1](http://www.ensembl.org/Rattus_norvegicus/geneview?gene=IPI00779150.1) |  |
|  | [ENSRNOG00000042776](http://www.ensembl.org/Rattus_norvegicus/Gene/Summary?db=core;g=ENSRNOG00000042776) | [1](http://www.ensembl.org/Rattus_norvegicus/mapview?chr=1) | [121165554](http://www.ensembl.org/Rattus_norvegicus/contigview?chr=1&vc_start=121165554&vc_end=121169007) | [121169007](http://www.ensembl.org/Rattus_norvegicus/contigview?chr=1&vc_start=121165554&vc_end=121169007) | [Lins1](http://www.ensembl.org/Rattus_norvegicus/geneview?gene=Lins1) |  |
|  | [ENSRNOG00000013823](http://www.ensembl.org/Rattus_norvegicus/Gene/Summary?db=core;g=ENSRNOG00000013823) | [1](http://www.ensembl.org/Rattus_norvegicus/mapview?chr=1) | [121193993](http://www.ensembl.org/Rattus_norvegicus/contigview?chr=1&vc_start=121193993&vc_end=121298746) | [121298746](http://www.ensembl.org/Rattus_norvegicus/contigview?chr=1&vc_start=121193993&vc_end=121298746) | [NP_001121033.1](http://www.ensembl.org/Rattus_norvegicus/geneview?gene=NP_001121033.1) | LAG1 homolog, ceramide synthase 3 |
|  | [ENSRNOG00000037080](http://www.ensembl.org/Rattus_norvegicus/Gene/Summary?db=core;g=ENSRNOG00000037080) | [1](http://www.ensembl.org/Rattus_norvegicus/mapview?chr=1) | [121324404](http://www.ensembl.org/Rattus_norvegicus/contigview?chr=1&vc_start=121324404&vc_end=121491069) | [121491069](http://www.ensembl.org/Rattus_norvegicus/contigview?chr=1&vc_start=121324404&vc_end=121491069) | [Adamts17](http://www.ensembl.org/Rattus_norvegicus/geneview?gene=Adamts17) |  |
|  | [ENSRNOG00000013877](http://www.ensembl.org/Rattus_norvegicus/Gene/Summary?db=core;g=ENSRNOG00000013877) | [1](http://www.ensembl.org/Rattus_norvegicus/mapview?chr=1) | [122402368](http://www.ensembl.org/Rattus_norvegicus/contigview?chr=1&vc_start=122402368&vc_end=122469865) | [122469865](http://www.ensembl.org/Rattus_norvegicus/contigview?chr=1&vc_start=122402368&vc_end=122469865) | [TTC23_RAT](http://www.ensembl.org/Rattus_norvegicus/geneview?gene=TTC23_RAT) | Tetratricopeptide repeat protein 23 |
|  | [ENSRNOG00000014167](http://www.ensembl.org/Rattus_norvegicus/Gene/Summary?db=core;g=ENSRNOG00000014167) | [1](http://www.ensembl.org/Rattus_norvegicus/mapview?chr=1) | [122545485](http://www.ensembl.org/Rattus_norvegicus/contigview?chr=1&vc_start=122545485&vc_end=122546024) | [122546024](http://www.ensembl.org/Rattus_norvegicus/contigview?chr=1&vc_start=122545485&vc_end=122546024) |  |  |
|  | [ENSRNOG00000014187](http://www.ensembl.org/Rattus_norvegicus/Gene/Summary?db=core;g=ENSRNOG00000014187) | [1](http://www.ensembl.org/Rattus_norvegicus/mapview?chr=1) | [122704987](http://www.ensembl.org/Rattus_norvegicus/contigview?chr=1&vc_start=122704987&vc_end=122990007) | [122990007](http://www.ensembl.org/Rattus_norvegicus/contigview?chr=1&vc_start=122704987&vc_end=122990007) | [IGF1R_RAT](http://www.ensembl.org/Rattus_norvegicus/geneview?gene=IGF1R_RAT) | Insulin-like growth factor 1 receptor Precursor |
|  | [ENSRNOG00000022822](http://www.ensembl.org/Rattus_norvegicus/Gene/Summary?db=core;g=ENSRNOG00000022822) | [1](http://www.ensembl.org/Rattus_norvegicus/mapview?chr=1) | [123057582](http://www.ensembl.org/Rattus_norvegicus/contigview?chr=1&vc_start=123057582&vc_end=123115563) | [123115563](http://www.ensembl.org/Rattus_norvegicus/contigview?chr=1&vc_start=123057582&vc_end=123115563) | [LOC680906](http://www.ensembl.org/Rattus_norvegicus/geneview?gene=LOC680906) |  |
|  | [ENSRNOG00000010043](http://www.ensembl.org/Rattus_norvegicus/Gene/Summary?db=core;g=ENSRNOG00000010043) | [1](http://www.ensembl.org/Rattus_norvegicus/mapview?chr=1) | [125470729](http://www.ensembl.org/Rattus_norvegicus/contigview?chr=1&vc_start=125470729&vc_end=125471962) | [125471962](http://www.ensembl.org/Rattus_norvegicus/contigview?chr=1&vc_start=125470729&vc_end=125471962) | [IPI00205626.4](http://www.ensembl.org/Rattus_norvegicus/geneview?gene=IPI00205626.4) |  |
|  | [ENSRNOG00000033319](http://www.ensembl.org/Rattus_norvegicus/Gene/Summary?db=core;g=ENSRNOG00000033319) | [1](http://www.ensembl.org/Rattus_norvegicus/mapview?chr=1) | [125544960](http://www.ensembl.org/Rattus_norvegicus/contigview?chr=1&vc_start=125544960&vc_end=125545646) | [125545646](http://www.ensembl.org/Rattus_norvegicus/contigview?chr=1&vc_start=125544960&vc_end=125545646) | [IPI00564263.2](http://www.ensembl.org/Rattus_norvegicus/geneview?gene=IPI00564263.2) |  |
|  | [ENSRNOG00000035191](http://www.ensembl.org/Rattus_norvegicus/Gene/Summary?db=core;g=ENSRNOG00000035191) | [1](http://www.ensembl.org/Rattus_norvegicus/mapview?chr=1) | [125730525](http://www.ensembl.org/Rattus_norvegicus/contigview?chr=1&vc_start=125730525&vc_end=125730631) | [125730631](http://www.ensembl.org/Rattus_norvegicus/contigview?chr=1&vc_start=125730525&vc_end=125730631) | [U6](http://www.ensembl.org/Rattus_norvegicus/geneview?gene=U6) | U6 spliceosomal RNA |
|  | [ENSRNOG00000043651](http://www.ensembl.org/Rattus_norvegicus/Gene/Summary?db=core;g=ENSRNOG00000043651) | [1](http://www.ensembl.org/Rattus_norvegicus/mapview?chr=1) | [127393595](http://www.ensembl.org/Rattus_norvegicus/contigview?chr=1&vc_start=127393595&vc_end=127393713) | [127393713](http://www.ensembl.org/Rattus_norvegicus/contigview?chr=1&vc_start=127393595&vc_end=127393713) |  |  |
|  | [ENSRNOG00000012874](http://www.ensembl.org/Rattus_norvegicus/Gene/Summary?db=core;g=ENSRNOG00000012874) | [1](http://www.ensembl.org/Rattus_norvegicus/mapview?chr=1) | [128547399](http://www.ensembl.org/Rattus_norvegicus/contigview?chr=1&vc_start=128547399&vc_end=128591304) | [128591304](http://www.ensembl.org/Rattus_norvegicus/contigview?chr=1&vc_start=128547399&vc_end=128591304) | [NP_001100994.1](http://www.ensembl.org/Rattus_norvegicus/geneview?gene=NP_001100994.1) | RGM domain family, member A |
|  | [ENSRNOG00000012461](http://www.ensembl.org/Rattus_norvegicus/Gene/Summary?db=core;g=ENSRNOG00000012461) | [1](http://www.ensembl.org/Rattus_norvegicus/mapview?chr=1) | [128844194](http://www.ensembl.org/Rattus_norvegicus/contigview?chr=1&vc_start=128844194&vc_end=128852576) | [128852576](http://www.ensembl.org/Rattus_norvegicus/contigview?chr=1&vc_start=128844194&vc_end=128852576) | [IPI00361978.2](http://www.ensembl.org/Rattus_norvegicus/geneview?gene=IPI00361978.2) |  |
|  | [ENSRNOG00000012369](http://www.ensembl.org/Rattus_norvegicus/Gene/Summary?db=core;g=ENSRNOG00000012369) | [1](http://www.ensembl.org/Rattus_norvegicus/mapview?chr=1) | [128936112](http://www.ensembl.org/Rattus_norvegicus/contigview?chr=1&vc_start=128936112&vc_end=128989452) | [128989452](http://www.ensembl.org/Rattus_norvegicus/contigview?chr=1&vc_start=128936112&vc_end=128989452) | [IPI00189515.1](http://www.ensembl.org/Rattus_norvegicus/geneview?gene=IPI00189515.1) |  |
|  | [ENSRNOG00000012031](http://www.ensembl.org/Rattus_norvegicus/Gene/Summary?db=core;g=ENSRNOG00000012031) | [1](http://www.ensembl.org/Rattus_norvegicus/mapview?chr=1) | [129158714](http://www.ensembl.org/Rattus_norvegicus/contigview?chr=1&vc_start=129158714&vc_end=129191810) | [129191810](http://www.ensembl.org/Rattus_norvegicus/contigview?chr=1&vc_start=129158714&vc_end=129191810) | [SIA8B_RAT](http://www.ensembl.org/Rattus_norvegicus/geneview?gene=SIA8B_RAT) | Alpha-2,8-sialyltransferase 8B |
|  | [ENSRNOG00000037061](http://www.ensembl.org/Rattus_norvegicus/Gene/Summary?db=core;g=ENSRNOG00000037061) | [1](http://www.ensembl.org/Rattus_norvegicus/mapview?chr=1) | [131052698](http://www.ensembl.org/Rattus_norvegicus/contigview?chr=1&vc_start=131052698&vc_end=131212817) | [131212817](http://www.ensembl.org/Rattus_norvegicus/contigview?chr=1&vc_start=131052698&vc_end=131212817) | [IPI00870320.2](http://www.ensembl.org/Rattus_norvegicus/geneview?gene=IPI00870320.2) |  |
|  | [ENSRNOG00000010964](http://www.ensembl.org/Rattus_norvegicus/Gene/Summary?db=core;g=ENSRNOG00000010964) | [1](http://www.ensembl.org/Rattus_norvegicus/mapview?chr=1) | [131224569](http://www.ensembl.org/Rattus_norvegicus/contigview?chr=1&vc_start=131224569&vc_end=131360512) | [131360512](http://www.ensembl.org/Rattus_norvegicus/contigview?chr=1&vc_start=131224569&vc_end=131360512) | [NP_001099741.2](http://www.ensembl.org/Rattus_norvegicus/geneview?gene=NP_001099741.2) | A kinase (PRKA) anchor protein 13 |
|  | [ENSRNOG00000010959](http://www.ensembl.org/Rattus_norvegicus/Gene/Summary?db=core;g=ENSRNOG00000010959) | [1](http://www.ensembl.org/Rattus_norvegicus/mapview?chr=1) | [131540949](http://www.ensembl.org/Rattus_norvegicus/contigview?chr=1&vc_start=131540949&vc_end=131566140) | [131566140](http://www.ensembl.org/Rattus_norvegicus/contigview?chr=1&vc_start=131540949&vc_end=131566140) | [ENC2_RAT](http://www.ensembl.org/Rattus_norvegicus/geneview?gene=ENC2_RAT) | **Ectoderm-neural cortex protein 2** |
|  | [ENSRNOG00000033460](http://www.ensembl.org/Rattus_norvegicus/Gene/Summary?db=core;g=ENSRNOG00000033460) | [1](http://www.ensembl.org/Rattus_norvegicus/mapview?chr=1) | [131572401](http://www.ensembl.org/Rattus_norvegicus/contigview?chr=1&vc_start=131572401&vc_end=131573212) | [131573212](http://www.ensembl.org/Rattus_norvegicus/contigview?chr=1&vc_start=131572401&vc_end=131573212) |  |  |
| **Parameters - SERT-/-** | **Ensembl Gene ID** | **Chr.** | **Gene Start** | **Gene End** | **Gene Name** | **Description** |
| Time in shelter | [ENSRNOG00000038931](http://www.ensembl.org/Rattus_norvegicus/Gene/Summary?db=core;g=ENSRNOG00000038931) | [11](http://www.ensembl.org/Rattus_norvegicus/mapview?chr=11) | [64659615](http://www.ensembl.org/Rattus_norvegicus/contigview?chr=11&vc_start=64659615&vc_end=64661621) | [64661621](http://www.ensembl.org/Rattus_norvegicus/contigview?chr=11&vc_start=64659615&vc_end=64661621) | [IPI00777499.1](http://www.ensembl.org/Rattus_norvegicus/geneview?gene=IPI00777499.1) |  |
|  | [ENSRNOG00000002721](http://www.ensembl.org/Rattus_norvegicus/Gene/Summary?db=core;g=ENSRNOG00000002721) | [11](http://www.ensembl.org/Rattus_norvegicus/mapview?chr=11) | [64852589](http://www.ensembl.org/Rattus_norvegicus/contigview?chr=11&vc_start=64852589&vc_end=64859549) | [64859549](http://www.ensembl.org/Rattus_norvegicus/contigview?chr=11&vc_start=64852589&vc_end=64859549) | [NP_001032415.1](http://www.ensembl.org/Rattus_norvegicus/geneview?gene=NP_001032415.1) | NADH dehydrogenase (ubiquinone) 1 beta subcomplex 4 |
|  | [ENSRNOG00000027116](http://www.ensembl.org/Rattus_norvegicus/Gene/Summary?db=core;g=ENSRNOG00000027116) | [11](http://www.ensembl.org/Rattus_norvegicus/mapview?chr=11) | [64862958](http://www.ensembl.org/Rattus_norvegicus/contigview?chr=11&vc_start=64862958&vc_end=64864696) | [64864696](http://www.ensembl.org/Rattus_norvegicus/contigview?chr=11&vc_start=64862958&vc_end=64864696) |  |  |
|  | [ENSRNOG00000026008](http://www.ensembl.org/Rattus_norvegicus/Gene/Summary?db=core;g=ENSRNOG00000026008) | [11](http://www.ensembl.org/Rattus_norvegicus/mapview?chr=11) | [64972306](http://www.ensembl.org/Rattus_norvegicus/contigview?chr=11&vc_start=64972306&vc_end=65006125) | [65006125](http://www.ensembl.org/Rattus_norvegicus/contigview?chr=11&vc_start=64972306&vc_end=65006125) | [NP_001094026.1](http://www.ensembl.org/Rattus_norvegicus/geneview?gene=NP_001094026.1) | general transcription factor IIE, polypeptide 1 (alpha subunit) |
|  | [ENSRNOG00000002496](http://www.ensembl.org/Rattus_norvegicus/Gene/Summary?db=core;g=ENSRNOG00000002496) | [11](http://www.ensembl.org/Rattus_norvegicus/mapview?chr=11) | [65129574](http://www.ensembl.org/Rattus_norvegicus/contigview?chr=11&vc_start=65129574&vc_end=65455115) | [65455115](http://www.ensembl.org/Rattus_norvegicus/contigview?chr=11&vc_start=65129574&vc_end=65455115) | [Stxbp5l](http://www.ensembl.org/Rattus_norvegicus/geneview?gene=Stxbp5l) |  |
|  | [ENSRNOG00000002459](http://www.ensembl.org/Rattus_norvegicus/Gene/Summary?db=core;g=ENSRNOG00000002459) | [11](http://www.ensembl.org/Rattus_norvegicus/mapview?chr=11) | [65608431](http://www.ensembl.org/Rattus_norvegicus/contigview?chr=11&vc_start=65608431&vc_end=65613124) | [65613124](http://www.ensembl.org/Rattus_norvegicus/contigview?chr=11&vc_start=65608431&vc_end=65613124) | [Fbxo40](http://www.ensembl.org/Rattus_norvegicus/geneview?gene=Fbxo40) |  |
|  | [ENSRNOG00000002350](http://www.ensembl.org/Rattus_norvegicus/Gene/Summary?db=core;g=ENSRNOG00000002350) | [11](http://www.ensembl.org/Rattus_norvegicus/mapview?chr=11) | [65763410](http://www.ensembl.org/Rattus_norvegicus/contigview?chr=11&vc_start=65763410&vc_end=65809967) | [65809967](http://www.ensembl.org/Rattus_norvegicus/contigview?chr=11&vc_start=65763410&vc_end=65809967) | [EAF2_RAT](http://www.ensembl.org/Rattus_norvegicus/geneview?gene=EAF2_RAT) | ELL-associated factor 2 |
|  | [ENSRNOG00000002305](http://www.ensembl.org/Rattus_norvegicus/Gene/Summary?db=core;g=ENSRNOG00000002305) | [11](http://www.ensembl.org/Rattus_norvegicus/mapview?chr=11) | [65819539](http://www.ensembl.org/Rattus_norvegicus/contigview?chr=11&vc_start=65819539&vc_end=65849928) | [65849928](http://www.ensembl.org/Rattus_norvegicus/contigview?chr=11&vc_start=65819539&vc_end=65849928) | [S15A2_RAT](http://www.ensembl.org/Rattus_norvegicus/geneview?gene=S15A2_RAT) | Solute carrier family 15 member 2 |
|  | [ENSRNOG00000038845](http://www.ensembl.org/Rattus_norvegicus/Gene/Summary?db=core;g=ENSRNOG00000038845) | [11](http://www.ensembl.org/Rattus_norvegicus/mapview?chr=11) | [65898239](http://www.ensembl.org/Rattus_norvegicus/contigview?chr=11&vc_start=65898239&vc_end=66260859) | [66260859](http://www.ensembl.org/Rattus_norvegicus/contigview?chr=11&vc_start=65898239&vc_end=66260859) | [IPI00777117.1](http://www.ensembl.org/Rattus_norvegicus/geneview?gene=IPI00777117.1) |  |
|  | [ENSRNOG00000033929](http://www.ensembl.org/Rattus_norvegicus/Gene/Summary?db=core;g=ENSRNOG00000033929) | [11](http://www.ensembl.org/Rattus_norvegicus/mapview?chr=11) | [66322275](http://www.ensembl.org/Rattus_norvegicus/contigview?chr=11&vc_start=66322275&vc_end=66324849) | [66324849](http://www.ensembl.org/Rattus_norvegicus/contigview?chr=11&vc_start=66322275&vc_end=66324849) | [Stfa2l2](http://www.ensembl.org/Rattus_norvegicus/geneview?gene=Stfa2l2) |  |
|  | [ENSRNOG00000028918](http://www.ensembl.org/Rattus_norvegicus/Gene/Summary?db=core;g=ENSRNOG00000028918) | [11](http://www.ensembl.org/Rattus_norvegicus/mapview?chr=11) | [66357732](http://www.ensembl.org/Rattus_norvegicus/contigview?chr=11&vc_start=66357732&vc_end=66360072) | [66360072](http://www.ensembl.org/Rattus_norvegicus/contigview?chr=11&vc_start=66357732&vc_end=66360072) |  |  |
|  | [ENSRNOG00000038831](http://www.ensembl.org/Rattus_norvegicus/Gene/Summary?db=core;g=ENSRNOG00000038831) | [11](http://www.ensembl.org/Rattus_norvegicus/mapview?chr=11) | [66418773](http://www.ensembl.org/Rattus_norvegicus/contigview?chr=11&vc_start=66418773&vc_end=66424924) | [66424924](http://www.ensembl.org/Rattus_norvegicus/contigview?chr=11&vc_start=66418773&vc_end=66424924) | [Stfa2](http://www.ensembl.org/Rattus_norvegicus/geneview?gene=Stfa2) |  |
|  | [ENSRNOG00000023953](http://www.ensembl.org/Rattus_norvegicus/Gene/Summary?db=core;g=ENSRNOG00000023953) | [11](http://www.ensembl.org/Rattus_norvegicus/mapview?chr=11) | [66456248](http://www.ensembl.org/Rattus_norvegicus/contigview?chr=11&vc_start=66456248&vc_end=66467253) | [66467253](http://www.ensembl.org/Rattus_norvegicus/contigview?chr=11&vc_start=66456248&vc_end=66467253) | [NP_001099346.1](http://www.ensembl.org/Rattus_norvegicus/geneview?gene=NP_001099346.1) | cystatin A (stefin A) |
|  | [ENSRNOG00000033941](http://www.ensembl.org/Rattus_norvegicus/Gene/Summary?db=core;g=ENSRNOG00000033941) | [11](http://www.ensembl.org/Rattus_norvegicus/mapview?chr=11) | [66475803](http://www.ensembl.org/Rattus_norvegicus/contigview?chr=11&vc_start=66475803&vc_end=66476529) | [66476529](http://www.ensembl.org/Rattus_norvegicus/contigview?chr=11&vc_start=66475803&vc_end=66476529) |  |  |
|  | [ENSRNOG00000002255](http://www.ensembl.org/Rattus_norvegicus/Gene/Summary?db=core;g=ENSRNOG00000002255) | [11](http://www.ensembl.org/Rattus_norvegicus/mapview?chr=11) | [66518069](http://www.ensembl.org/Rattus_norvegicus/contigview?chr=11&vc_start=66518069&vc_end=66546302) | [66546302](http://www.ensembl.org/Rattus_norvegicus/contigview?chr=11&vc_start=66518069&vc_end=66546302) | [F162A_RAT](http://www.ensembl.org/Rattus_norvegicus/geneview?gene=F162A_RAT) | UPF0389 protein FAM162A |
|  | [ENSRNOG00000023400](http://www.ensembl.org/Rattus_norvegicus/Gene/Summary?db=core;g=ENSRNOG00000023400) | [11](http://www.ensembl.org/Rattus_norvegicus/mapview?chr=11) | [66649508](http://www.ensembl.org/Rattus_norvegicus/contigview?chr=11&vc_start=66649508&vc_end=66659119) | [66659119](http://www.ensembl.org/Rattus_norvegicus/contigview?chr=11&vc_start=66649508&vc_end=66659119) | [NP_001102523.1](http://www.ensembl.org/Rattus_norvegicus/geneview?gene=NP_001102523.1) | deltex 3-like |
|  | [ENSRNOG00000042343](http://www.ensembl.org/Rattus_norvegicus/Gene/Summary?db=core;g=ENSRNOG00000042343) | [11](http://www.ensembl.org/Rattus_norvegicus/mapview?chr=11) | [66736902](http://www.ensembl.org/Rattus_norvegicus/contigview?chr=11&vc_start=66736902&vc_end=66748278) | [66748278](http://www.ensembl.org/Rattus_norvegicus/contigview?chr=11&vc_start=66736902&vc_end=66748278) | [IPI00392050.3](http://www.ensembl.org/Rattus_norvegicus/geneview?gene=IPI00392050.3) |  |
|  | [ENSRNOG00000023334](http://www.ensembl.org/Rattus_norvegicus/Gene/Summary?db=core;g=ENSRNOG00000023334) | [11](http://www.ensembl.org/Rattus_norvegicus/mapview?chr=11) | [66751986](http://www.ensembl.org/Rattus_norvegicus/contigview?chr=11&vc_start=66751986&vc_end=66767306) | [66767306](http://www.ensembl.org/Rattus_norvegicus/contigview?chr=11&vc_start=66751986&vc_end=66767306) | [Parp14](http://www.ensembl.org/Rattus_norvegicus/geneview?gene=Parp14) | Poly [ADP-ribose] polymerase 14 |
|  | [ENSRNOG00000002240](http://www.ensembl.org/Rattus_norvegicus/Gene/Summary?db=core;g=ENSRNOG00000002240) | [11](http://www.ensembl.org/Rattus_norvegicus/mapview?chr=11) | [66829715](http://www.ensembl.org/Rattus_norvegicus/contigview?chr=11&vc_start=66829715&vc_end=66910198) | [66910198](http://www.ensembl.org/Rattus_norvegicus/contigview?chr=11&vc_start=66829715&vc_end=66910198) | [DIRC2_RAT](http://www.ensembl.org/Rattus_norvegicus/geneview?gene=DIRC2_RAT) | Disrupted in renal carcinoma protein 2 homolog |
|  | [ENSRNOG00000032327](http://www.ensembl.org/Rattus_norvegicus/Gene/Summary?db=core;g=ENSRNOG00000032327) | [11](http://www.ensembl.org/Rattus_norvegicus/mapview?chr=11) | [67117749](http://www.ensembl.org/Rattus_norvegicus/contigview?chr=11&vc_start=67117749&vc_end=67204478) | [67204478](http://www.ensembl.org/Rattus_norvegicus/contigview?chr=11&vc_start=67117749&vc_end=67204478) | [PDIA5_RAT](http://www.ensembl.org/Rattus_norvegicus/geneview?gene=PDIA5_RAT) | Protein disulfide-isomerase A5 Precursor |
|  | [ENSRNOG00000043069](http://www.ensembl.org/Rattus_norvegicus/Gene/Summary?db=core;g=ENSRNOG00000043069) | [11](http://www.ensembl.org/Rattus_norvegicus/mapview?chr=11) | [67267957](http://www.ensembl.org/Rattus_norvegicus/contigview?chr=11&vc_start=67267957&vc_end=67281675) | [67281675](http://www.ensembl.org/Rattus_norvegicus/contigview?chr=11&vc_start=67267957&vc_end=67281675) | [SC22A_RAT](http://www.ensembl.org/Rattus_norvegicus/geneview?gene=SC22A_RAT) | Vesicle-trafficking protein SEC22a |
|  | [ENSRNOG00000028737](http://www.ensembl.org/Rattus_norvegicus/Gene/Summary?db=core;g=ENSRNOG00000028737) | [11](http://www.ensembl.org/Rattus_norvegicus/mapview?chr=11) | [67933969](http://www.ensembl.org/Rattus_norvegicus/contigview?chr=11&vc_start=67933969&vc_end=67934751) | [67934751](http://www.ensembl.org/Rattus_norvegicus/contigview?chr=11&vc_start=67933969&vc_end=67934751) |  |  |
|  | [ENSRNOG00000001706](http://www.ensembl.org/Rattus_norvegicus/Gene/Summary?db=core;g=ENSRNOG00000001706) | [11](http://www.ensembl.org/Rattus_norvegicus/mapview?chr=11) | [68195340](http://www.ensembl.org/Rattus_norvegicus/contigview?chr=11&vc_start=68195340&vc_end=68611336) | [68611336](http://www.ensembl.org/Rattus_norvegicus/contigview?chr=11&vc_start=68195340&vc_end=68611336) | [KALRN_RAT](http://www.ensembl.org/Rattus_norvegicus/geneview?gene=KALRN_RAT) | Kalirin |
|  | [ENSRNOG00000001797](http://www.ensembl.org/Rattus_norvegicus/Gene/Summary?db=core;g=ENSRNOG00000001797) | [11](http://www.ensembl.org/Rattus_norvegicus/mapview?chr=11) | [68619835](http://www.ensembl.org/Rattus_norvegicus/contigview?chr=11&vc_start=68619835&vc_end=68630244) | [68630244](http://www.ensembl.org/Rattus_norvegicus/contigview?chr=11&vc_start=68619835&vc_end=68630244) | [NP_001020573.1](http://www.ensembl.org/Rattus_norvegicus/geneview?gene=NP_001020573.1) | uridine monophosphate synthase |
|  | [ENSRNOG00000026420](http://www.ensembl.org/Rattus_norvegicus/Gene/Summary?db=core;g=ENSRNOG00000026420) | [11](http://www.ensembl.org/Rattus_norvegicus/mapview?chr=11) | [69553215](http://www.ensembl.org/Rattus_norvegicus/contigview?chr=11&vc_start=69553215&vc_end=69591042) | [69591042](http://www.ensembl.org/Rattus_norvegicus/contigview?chr=11&vc_start=69553215&vc_end=69591042) | [IQCG_RAT](http://www.ensembl.org/Rattus_norvegicus/geneview?gene=IQCG_RAT) | IQ domain-containing protein G |
|  | [ENSRNOG00000025885](http://www.ensembl.org/Rattus_norvegicus/Gene/Summary?db=core;g=ENSRNOG00000025885) | [11](http://www.ensembl.org/Rattus_norvegicus/mapview?chr=11) | [69735303](http://www.ensembl.org/Rattus_norvegicus/contigview?chr=11&vc_start=69735303&vc_end=69782506) | [69782506](http://www.ensembl.org/Rattus_norvegicus/contigview?chr=11&vc_start=69735303&vc_end=69782506) | [RGD1305422](http://www.ensembl.org/Rattus_norvegicus/geneview?gene=RGD1305422) |  |
|  | [ENSRNOG00000001776](http://www.ensembl.org/Rattus_norvegicus/Gene/Summary?db=core;g=ENSRNOG00000001776) | [11](http://www.ensembl.org/Rattus_norvegicus/mapview?chr=11) | [69808275](http://www.ensembl.org/Rattus_norvegicus/contigview?chr=11&vc_start=69808275&vc_end=69823525) | [69823525](http://www.ensembl.org/Rattus_norvegicus/contigview?chr=11&vc_start=69808275&vc_end=69823525) | [NP_001100559.1](http://www.ensembl.org/Rattus_norvegicus/geneview?gene=NP_001100559.1) | mucin 20, cell surface associated |
|  | [ENSRNOG00000038635](http://www.ensembl.org/Rattus_norvegicus/Gene/Summary?db=core;g=ENSRNOG00000038635) | [11](http://www.ensembl.org/Rattus_norvegicus/mapview?chr=11) | [69936508](http://www.ensembl.org/Rattus_norvegicus/contigview?chr=11&vc_start=69936508&vc_end=70068900) | [70068900](http://www.ensembl.org/Rattus_norvegicus/contigview?chr=11&vc_start=69936508&vc_end=70068900) | [IPI00780670.1](http://www.ensembl.org/Rattus_norvegicus/geneview?gene=IPI00780670.1) |  |
|  | [ENSRNOG00000001765](http://www.ensembl.org/Rattus_norvegicus/Gene/Summary?db=core;g=ENSRNOG00000001765) | [11](http://www.ensembl.org/Rattus_norvegicus/mapview?chr=11) | [70115430](http://www.ensembl.org/Rattus_norvegicus/contigview?chr=11&vc_start=70115430&vc_end=70129828) | [70129828](http://www.ensembl.org/Rattus_norvegicus/contigview?chr=11&vc_start=70115430&vc_end=70129828) | [NP_001100557.1](http://www.ensembl.org/Rattus_norvegicus/geneview?gene=NP_001100557.1) | organic solute transporter alpha |
|  | [ENSRNOG00000001753](http://www.ensembl.org/Rattus_norvegicus/Gene/Summary?db=core;g=ENSRNOG00000001753) | [11](http://www.ensembl.org/Rattus_norvegicus/mapview?chr=11) | [70371625](http://www.ensembl.org/Rattus_norvegicus/contigview?chr=11&vc_start=70371625&vc_end=70383636) | [70383636](http://www.ensembl.org/Rattus_norvegicus/contigview?chr=11&vc_start=70371625&vc_end=70383636) | [Fbxo45](http://www.ensembl.org/Rattus_norvegicus/geneview?gene=Fbxo45) | F-box/SPRY domain-containing protein 1 |
|  | [ENSRNOG00000001752](http://www.ensembl.org/Rattus_norvegicus/Gene/Summary?db=core;g=ENSRNOG00000001752) | [11](http://www.ensembl.org/Rattus_norvegicus/mapview?chr=11) | [70449116](http://www.ensembl.org/Rattus_norvegicus/contigview?chr=11&vc_start=70449116&vc_end=70466402) | [70466402](http://www.ensembl.org/Rattus_norvegicus/contigview?chr=11&vc_start=70449116&vc_end=70466402) | [LRC33_RAT](http://www.ensembl.org/Rattus_norvegicus/geneview?gene=LRC33_RAT) | Leucine-rich repeat-containing protein 33 Precursor |
|  | [ENSRNOG00000033623](http://www.ensembl.org/Rattus_norvegicus/Gene/Summary?db=core;g=ENSRNOG00000033623) | [11](http://www.ensembl.org/Rattus_norvegicus/mapview?chr=11) | [70508995](http://www.ensembl.org/Rattus_norvegicus/contigview?chr=11&vc_start=70508995&vc_end=70524957) | [70524957](http://www.ensembl.org/Rattus_norvegicus/contigview?chr=11&vc_start=70508995&vc_end=70524957) | [PIGX_RAT](http://www.ensembl.org/Rattus_norvegicus/geneview?gene=PIGX_RAT) | Phosphatidylinositol-glycan biosynthesis class X protein Precursor |
|  | [ENSRNOG00000001747](http://www.ensembl.org/Rattus_norvegicus/Gene/Summary?db=core;g=ENSRNOG00000001747) | [11](http://www.ensembl.org/Rattus_norvegicus/mapview?chr=11) | [70529961](http://www.ensembl.org/Rattus_norvegicus/contigview?chr=11&vc_start=70529961&vc_end=70588515) | [70588515](http://www.ensembl.org/Rattus_norvegicus/contigview?chr=11&vc_start=70529961&vc_end=70588515) | [PAK2_RAT](http://www.ensembl.org/Rattus_norvegicus/geneview?gene=PAK2_RAT) | **Serine/threonine-protein kinase PAK 2** |
|  | [ENSRNOG00000024794](http://www.ensembl.org/Rattus_norvegicus/Gene/Summary?db=core;g=ENSRNOG00000024794) | [11](http://www.ensembl.org/Rattus_norvegicus/mapview?chr=11) | [70615144](http://www.ensembl.org/Rattus_norvegicus/contigview?chr=11&vc_start=70615144&vc_end=70638148) | [70638148](http://www.ensembl.org/Rattus_norvegicus/contigview?chr=11&vc_start=70615144&vc_end=70638148) | [Senp5](http://www.ensembl.org/Rattus_norvegicus/geneview?gene=Senp5) |  |
|  | [ENSRNOG00000001733](http://www.ensembl.org/Rattus_norvegicus/Gene/Summary?db=core;g=ENSRNOG00000001733) | [11](http://www.ensembl.org/Rattus_norvegicus/mapview?chr=11) | [71319294](http://www.ensembl.org/Rattus_norvegicus/contigview?chr=11&vc_start=71319294&vc_end=71342715) | [71342715](http://www.ensembl.org/Rattus_norvegicus/contigview?chr=11&vc_start=71319294&vc_end=71342715) | [IPP2_RAT](http://www.ensembl.org/Rattus_norvegicus/geneview?gene=IPP2_RAT) | Protein phosphatase inhibitor 2 (IPP-2) |
|  | [ENSRNOG00000001732](http://www.ensembl.org/Rattus_norvegicus/Gene/Summary?db=core;g=ENSRNOG00000001732) | [11](http://www.ensembl.org/Rattus_norvegicus/mapview?chr=11) | [71328053](http://www.ensembl.org/Rattus_norvegicus/contigview?chr=11&vc_start=71328053&vc_end=71328492) | [71328492](http://www.ensembl.org/Rattus_norvegicus/contigview?chr=11&vc_start=71328053&vc_end=71328492) |  |  |
|  | [ENSRNOG00000001730](http://www.ensembl.org/Rattus_norvegicus/Gene/Summary?db=core;g=ENSRNOG00000001730) | [11](http://www.ensembl.org/Rattus_norvegicus/mapview?chr=11) | [71411706](http://www.ensembl.org/Rattus_norvegicus/contigview?chr=11&vc_start=71411706&vc_end=71524825) | [71524825](http://www.ensembl.org/Rattus_norvegicus/contigview?chr=11&vc_start=71411706&vc_end=71524825) | [ACAP2_RAT](http://www.ensembl.org/Rattus_norvegicus/geneview?gene=ACAP2_RAT) | ARFGAP with ANK repeat and PH domain-containing protein 2 |
|  | [ENSRNOG00000001729](http://www.ensembl.org/Rattus_norvegicus/Gene/Summary?db=core;g=ENSRNOG00000001729) | [11](http://www.ensembl.org/Rattus_norvegicus/mapview?chr=11) | [71532958](http://www.ensembl.org/Rattus_norvegicus/contigview?chr=11&vc_start=71532958&vc_end=71658701) | [71658701](http://www.ensembl.org/Rattus_norvegicus/contigview?chr=11&vc_start=71532958&vc_end=71658701) | [RGD1308154](http://www.ensembl.org/Rattus_norvegicus/geneview?gene=RGD1308154) |  |
|  | [ENSRNOG00000001727](http://www.ensembl.org/Rattus_norvegicus/Gene/Summary?db=core;g=ENSRNOG00000001727) | [11](http://www.ensembl.org/Rattus_norvegicus/mapview?chr=11) | [72026815](http://www.ensembl.org/Rattus_norvegicus/contigview?chr=11&vc_start=72026815&vc_end=72051400) | [72051400](http://www.ensembl.org/Rattus_norvegicus/contigview?chr=11&vc_start=72026815&vc_end=72051400) | [LSG1_RAT](http://www.ensembl.org/Rattus_norvegicus/geneview?gene=LSG1_RAT) | Large subunit GTPase 1 homolog |
|  | [ENSRNOG00000001726](http://www.ensembl.org/Rattus_norvegicus/Gene/Summary?db=core;g=ENSRNOG00000001726) | [11](http://www.ensembl.org/Rattus_norvegicus/mapview?chr=11) | [72062843](http://www.ensembl.org/Rattus_norvegicus/contigview?chr=11&vc_start=72062843&vc_end=72083318) | [72083318](http://www.ensembl.org/Rattus_norvegicus/contigview?chr=11&vc_start=72062843&vc_end=72083318) | [Tmem44](http://www.ensembl.org/Rattus_norvegicus/geneview?gene=Tmem44) |  |
|  | [ENSRNOG00000001724](http://www.ensembl.org/Rattus_norvegicus/Gene/Summary?db=core;g=ENSRNOG00000001724) | [11](http://www.ensembl.org/Rattus_norvegicus/mapview?chr=11) | [72263084](http://www.ensembl.org/Rattus_norvegicus/contigview?chr=11&vc_start=72263084&vc_end=72336927) | [72336927](http://www.ensembl.org/Rattus_norvegicus/contigview?chr=11&vc_start=72263084&vc_end=72336927) | [NP_001102839.1](http://www.ensembl.org/Rattus_norvegicus/geneview?gene=NP_001102839.1) | hypothetical protein LOC678704 |
|  | [ENSRNOG00000038540](http://www.ensembl.org/Rattus_norvegicus/Gene/Summary?db=core;g=ENSRNOG00000038540) | [11](http://www.ensembl.org/Rattus_norvegicus/mapview?chr=11) | [72341773](http://www.ensembl.org/Rattus_norvegicus/contigview?chr=11&vc_start=72341773&vc_end=72344865) | [72344865](http://www.ensembl.org/Rattus_norvegicus/contigview?chr=11&vc_start=72341773&vc_end=72344865) | [GPV_RAT](http://www.ensembl.org/Rattus_norvegicus/geneview?gene=GPV_RAT) | Platelet glycoprotein V Precursor |
|  | [ENSRNOG00000038539](http://www.ensembl.org/Rattus_norvegicus/Gene/Summary?db=core;g=ENSRNOG00000038539) | [11](http://www.ensembl.org/Rattus_norvegicus/mapview?chr=11) | [72377090](http://www.ensembl.org/Rattus_norvegicus/contigview?chr=11&vc_start=72377090&vc_end=72378263) | [72378263](http://www.ensembl.org/Rattus_norvegicus/contigview?chr=11&vc_start=72377090&vc_end=72378263) | [NM_145083.1](http://www.ensembl.org/Rattus_norvegicus/geneview?gene=NM_145083.1) | leucine rich repeat containing 15 (Lrrc15) |
|  | [ENSRNOG00000024384](http://www.ensembl.org/Rattus_norvegicus/Gene/Summary?db=core;g=ENSRNOG00000024384) | [11](http://www.ensembl.org/Rattus_norvegicus/mapview?chr=11) | [72384307](http://www.ensembl.org/Rattus_norvegicus/contigview?chr=11&vc_start=72384307&vc_end=72393946) | [72393946](http://www.ensembl.org/Rattus_norvegicus/contigview?chr=11&vc_start=72384307&vc_end=72393946) | [NP_001100555.1](http://www.ensembl.org/Rattus_norvegicus/geneview?gene=NP_001100555.1) | carboxypeptidase N, polypeptide 2 |
|  | [ENSRNOG00000001714](http://www.ensembl.org/Rattus_norvegicus/Gene/Summary?db=core;g=ENSRNOG00000001714) | [11](http://www.ensembl.org/Rattus_norvegicus/mapview?chr=11) | [73167332](http://www.ensembl.org/Rattus_norvegicus/contigview?chr=11&vc_start=73167332&vc_end=73251065) | [73251065](http://www.ensembl.org/Rattus_norvegicus/contigview?chr=11&vc_start=73167332&vc_end=73251065) | [Atp13a4](http://www.ensembl.org/Rattus_norvegicus/geneview?gene=Atp13a4) |  |
|  | [ENSRNOG00000024127](http://www.ensembl.org/Rattus_norvegicus/Gene/Summary?db=core;g=ENSRNOG00000024127) | [11](http://www.ensembl.org/Rattus_norvegicus/mapview?chr=11) | [73293142](http://www.ensembl.org/Rattus_norvegicus/contigview?chr=11&vc_start=73293142&vc_end=73418275) | [73418275](http://www.ensembl.org/Rattus_norvegicus/contigview?chr=11&vc_start=73293142&vc_end=73418275) | [Atp13a5](http://www.ensembl.org/Rattus_norvegicus/geneview?gene=Atp13a5) |  |
|  | [ENSRNOG00000038518](http://www.ensembl.org/Rattus_norvegicus/Gene/Summary?db=core;g=ENSRNOG00000038518) | [11](http://www.ensembl.org/Rattus_norvegicus/mapview?chr=11) | [73595573](http://www.ensembl.org/Rattus_norvegicus/contigview?chr=11&vc_start=73595573&vc_end=73616895) | [73616895](http://www.ensembl.org/Rattus_norvegicus/contigview?chr=11&vc_start=73595573&vc_end=73616895) |  |  |
|  | [ENSRNOG00000024040](http://www.ensembl.org/Rattus_norvegicus/Gene/Summary?db=core;g=ENSRNOG00000024040) | [11](http://www.ensembl.org/Rattus_norvegicus/mapview?chr=11) | [73733241](http://www.ensembl.org/Rattus_norvegicus/contigview?chr=11&vc_start=73733241&vc_end=73831764) | [73831764](http://www.ensembl.org/Rattus_norvegicus/contigview?chr=11&vc_start=73733241&vc_end=73831764) | [NP_001102526.2](http://www.ensembl.org/Rattus_norvegicus/geneview?gene=NP_001102526.2) | hypothetical protein LOC498100 |
|  | [ENSRNOG00000001931](http://www.ensembl.org/Rattus_norvegicus/Gene/Summary?db=core;g=ENSRNOG00000001931) | [11](http://www.ensembl.org/Rattus_norvegicus/mapview?chr=11) | [74208696](http://www.ensembl.org/Rattus_norvegicus/contigview?chr=11&vc_start=74208696&vc_end=74481037) | [74481037](http://www.ensembl.org/Rattus_norvegicus/contigview?chr=11&vc_start=74208696&vc_end=74481037) | [FGF12_RAT](http://www.ensembl.org/Rattus_norvegicus/geneview?gene=FGF12_RAT) | **Fibroblast growth factor 12 (FGF-12)** |
|  | [ENSRNOG00000038512](http://www.ensembl.org/Rattus_norvegicus/Gene/Summary?db=core;g=ENSRNOG00000038512) | [11](http://www.ensembl.org/Rattus_norvegicus/mapview?chr=11) | [75389917](http://www.ensembl.org/Rattus_norvegicus/contigview?chr=11&vc_start=75389917&vc_end=75424944) | [75424944](http://www.ensembl.org/Rattus_norvegicus/contigview?chr=11&vc_start=75389917&vc_end=75424944) | [Uts2d](http://www.ensembl.org/Rattus_norvegicus/geneview?gene=Uts2d) | Urotensin-2B Precursor |
|  | [ENSRNOG00000038505](http://www.ensembl.org/Rattus_norvegicus/Gene/Summary?db=core;g=ENSRNOG00000038505) | [11](http://www.ensembl.org/Rattus_norvegicus/mapview?chr=11) | [75829765](http://www.ensembl.org/Rattus_norvegicus/contigview?chr=11&vc_start=75829765&vc_end=75835294) | [75835294](http://www.ensembl.org/Rattus_norvegicus/contigview?chr=11&vc_start=75829765&vc_end=75835294) | [RGD1563170](http://www.ensembl.org/Rattus_norvegicus/geneview?gene=RGD1563170) |  |
|  | [ENSRNOG00000001926](http://www.ensembl.org/Rattus_norvegicus/Gene/Summary?db=core;g=ENSRNOG00000001926) | [11](http://www.ensembl.org/Rattus_norvegicus/mapview?chr=11) | [76473654](http://www.ensembl.org/Rattus_norvegicus/contigview?chr=11&vc_start=76473654&vc_end=76488805) | [76488805](http://www.ensembl.org/Rattus_norvegicus/contigview?chr=11&vc_start=76473654&vc_end=76488805) | [CLD1_RAT](http://www.ensembl.org/Rattus_norvegicus/geneview?gene=CLD1_RAT) | Claudin-1 |
|  | [ENSRNOG00000001925](http://www.ensembl.org/Rattus_norvegicus/Gene/Summary?db=core;g=ENSRNOG00000001925) | [11](http://www.ensembl.org/Rattus_norvegicus/mapview?chr=11) | [76687590](http://www.ensembl.org/Rattus_norvegicus/contigview?chr=11&vc_start=76687590&vc_end=76828687) | [76828687](http://www.ensembl.org/Rattus_norvegicus/contigview?chr=11&vc_start=76687590&vc_end=76828687) | [P3H2_RAT](http://www.ensembl.org/Rattus_norvegicus/geneview?gene=P3H2_RAT) | Prolyl 3-hydroxylase 2 Precursor |
|  | [ENSRNOG00000022687](http://www.ensembl.org/Rattus_norvegicus/Gene/Summary?db=core;g=ENSRNOG00000022687) | [11](http://www.ensembl.org/Rattus_norvegicus/mapview?chr=11) | [76732240](http://www.ensembl.org/Rattus_norvegicus/contigview?chr=11&vc_start=76732240&vc_end=76733137) | [76733137](http://www.ensembl.org/Rattus_norvegicus/contigview?chr=11&vc_start=76732240&vc_end=76733137) |  |  |
|  | [ENSRNOG00000042175](http://www.ensembl.org/Rattus_norvegicus/Gene/Summary?db=core;g=ENSRNOG00000042175) | [11](http://www.ensembl.org/Rattus_norvegicus/mapview?chr=11) | [64631466](http://www.ensembl.org/Rattus_norvegicus/contigview?chr=11&vc_start=64631466&vc_end=64632612) | [64632612](http://www.ensembl.org/Rattus_norvegicus/contigview?chr=11&vc_start=64631466&vc_end=64632612) | [B6VQA7_RAT](http://www.ensembl.org/Rattus_norvegicus/geneview?gene=B6VQA7_RAT) | Putative uncharacterized protein |
|  | [ENSRNOG00000026991](http://www.ensembl.org/Rattus_norvegicus/Gene/Summary?db=core;g=ENSRNOG00000026991) | [11](http://www.ensembl.org/Rattus_norvegicus/mapview?chr=11) | [69277175](http://www.ensembl.org/Rattus_norvegicus/contigview?chr=11&vc_start=69277175&vc_end=69278008) | [69278008](http://www.ensembl.org/Rattus_norvegicus/contigview?chr=11&vc_start=69277175&vc_end=69278008) | [RGD1559590](http://www.ensembl.org/Rattus_norvegicus/geneview?gene=RGD1559590) |  |
|  | [ENSRNOG00000031450](http://www.ensembl.org/Rattus_norvegicus/Gene/Summary?db=core;g=ENSRNOG00000031450) | [11](http://www.ensembl.org/Rattus_norvegicus/mapview?chr=11) | [72257082](http://www.ensembl.org/Rattus_norvegicus/contigview?chr=11&vc_start=72257082&vc_end=72257496) | [72257496](http://www.ensembl.org/Rattus_norvegicus/contigview?chr=11&vc_start=72257082&vc_end=72257496) | [RGD1562415](http://www.ensembl.org/Rattus_norvegicus/geneview?gene=RGD1562415) |  |
|  | [ENSRNOG00000023917](http://www.ensembl.org/Rattus_norvegicus/Gene/Summary?db=core;g=ENSRNOG00000023917) | [11](http://www.ensembl.org/Rattus_norvegicus/mapview?chr=11) | [66482490](http://www.ensembl.org/Rattus_norvegicus/contigview?chr=11&vc_start=66482490&vc_end=66483101) | [66483101](http://www.ensembl.org/Rattus_norvegicus/contigview?chr=11&vc_start=66482490&vc_end=66483101) | [XM_001070451.1](http://www.ensembl.org/Rattus_norvegicus/geneview?gene=XM_001070451.1) |  |
|  | [ENSRNOG00000041476](http://www.ensembl.org/Rattus_norvegicus/Gene/Summary?db=core;g=ENSRNOG00000041476) | [11](http://www.ensembl.org/Rattus_norvegicus/mapview?chr=11) | [69255834](http://www.ensembl.org/Rattus_norvegicus/contigview?chr=11&vc_start=69255834&vc_end=69255909) | [69255909](http://www.ensembl.org/Rattus_norvegicus/contigview?chr=11&vc_start=69255834&vc_end=69255909) | [SNORD111](http://www.ensembl.org/Rattus_norvegicus/geneview?gene=SNORD111) | Small Nucleolar RNA SNORD111 |
|  | [ENSRNOG00000040667](http://www.ensembl.org/Rattus_norvegicus/Gene/Summary?db=core;g=ENSRNOG00000040667) | [11](http://www.ensembl.org/Rattus_norvegicus/mapview?chr=11) | [66065354](http://www.ensembl.org/Rattus_norvegicus/contigview?chr=11&vc_start=66065354&vc_end=66065422) | [66065422](http://www.ensembl.org/Rattus_norvegicus/contigview?chr=11&vc_start=66065354&vc_end=66065422) | [SNORD29](http://www.ensembl.org/Rattus_norvegicus/geneview?gene=SNORD29) | Small nucleolar RNA SNORD29 |
|  | [ENSRNOG00000036327](http://www.ensembl.org/Rattus_norvegicus/Gene/Summary?db=core;g=ENSRNOG00000036327) | [11](http://www.ensembl.org/Rattus_norvegicus/mapview?chr=11) | [74057906](http://www.ensembl.org/Rattus_norvegicus/contigview?chr=11&vc_start=74057906&vc_end=74058015) | [74058015](http://www.ensembl.org/Rattus_norvegicus/contigview?chr=11&vc_start=74057906&vc_end=74058015) |  |  |
|  | [ENSRNOG00000036328](http://www.ensembl.org/Rattus_norvegicus/Gene/Summary?db=core;g=ENSRNOG00000036328) | [11](http://www.ensembl.org/Rattus_norvegicus/mapview?chr=11) | [72549437](http://www.ensembl.org/Rattus_norvegicus/contigview?chr=11&vc_start=72549437&vc_end=72549531) | [72549531](http://www.ensembl.org/Rattus_norvegicus/contigview?chr=11&vc_start=72549437&vc_end=72549531) |  |  |
|  | [ENSRNOG00000035756](http://www.ensembl.org/Rattus_norvegicus/Gene/Summary?db=core;g=ENSRNOG00000035756) | [11](http://www.ensembl.org/Rattus_norvegicus/mapview?chr=11) | [70013839](http://www.ensembl.org/Rattus_norvegicus/contigview?chr=11&vc_start=70013839&vc_end=70013945) | [70013945](http://www.ensembl.org/Rattus_norvegicus/contigview?chr=11&vc_start=70013839&vc_end=70013945) | [U6](http://www.ensembl.org/Rattus_norvegicus/geneview?gene=U6) | U6 spliceosomal RNA |
|  | [ENSRNOG00000041452](http://www.ensembl.org/Rattus_norvegicus/Gene/Summary?db=core;g=ENSRNOG00000041452) | [11](http://www.ensembl.org/Rattus_norvegicus/mapview?chr=11) | [70374717](http://www.ensembl.org/Rattus_norvegicus/contigview?chr=11&vc_start=70374717&vc_end=70374859) | [70374859](http://www.ensembl.org/Rattus_norvegicus/contigview?chr=11&vc_start=70374717&vc_end=70374859) | [SNORA79](http://www.ensembl.org/Rattus_norvegicus/geneview?gene=SNORA79) | Small nucleolar RNA SNORA79 |
|  | [ENSRNOG00000041667](http://www.ensembl.org/Rattus_norvegicus/Gene/Summary?db=core;g=ENSRNOG00000041667) | [11](http://www.ensembl.org/Rattus_norvegicus/mapview?chr=11) | [72509918](http://www.ensembl.org/Rattus_norvegicus/contigview?chr=11&vc_start=72509918&vc_end=72510023) | [72510023](http://www.ensembl.org/Rattus_norvegicus/contigview?chr=11&vc_start=72509918&vc_end=72510023) | [5S_rRNA](http://www.ensembl.org/Rattus_norvegicus/geneview?gene=5S_rRNA) | 5S ribosomal RNA |
| **Parameters - SERT-/-** | **Ensembl Gene ID** | **Chr.** | **Gene Start** | **Gene End** | **Gene Name** | **Description** |
| Time in shelter | [ENSRNOG00000002209](http://www.ensembl.org/Rattus_norvegicus/Gene/Summary?db=core;g=ENSRNOG00000002209) | [14](http://www.ensembl.org/Rattus_norvegicus/mapview?chr=14) | [50609443](http://www.ensembl.org/Rattus_norvegicus/contigview?chr=14&vc_start=50609443&vc_end=50789829) | [50789829](http://www.ensembl.org/Rattus_norvegicus/contigview?chr=14&vc_start=50609443&vc_end=50789829) | [NP_001100686.1](http://www.ensembl.org/Rattus_norvegicus/geneview?gene=NP_001100686.1) | ArfGAP with RhoGAP domain, ankyrin repeat and PH domain 2 |
|  | [ENSRNOG00000015015](http://www.ensembl.org/Rattus_norvegicus/Gene/Summary?db=core;g=ENSRNOG00000015015) | [14](http://www.ensembl.org/Rattus_norvegicus/mapview?chr=14) | [51487386](http://www.ensembl.org/Rattus_norvegicus/contigview?chr=14&vc_start=51487386&vc_end=51489562) | [51489562](http://www.ensembl.org/Rattus_norvegicus/contigview?chr=14&vc_start=51487386&vc_end=51489562) | [IPI00567593.2](http://www.ensembl.org/Rattus_norvegicus/geneview?gene=IPI00567593.2) |  |
|  | [ENSRNOG00000028835](http://www.ensembl.org/Rattus_norvegicus/Gene/Summary?db=core;g=ENSRNOG00000028835) | [14](http://www.ensembl.org/Rattus_norvegicus/mapview?chr=14) | [54840966](http://www.ensembl.org/Rattus_norvegicus/contigview?chr=14&vc_start=54840966&vc_end=54842637) | [54842637](http://www.ensembl.org/Rattus_norvegicus/contigview?chr=14&vc_start=54840966&vc_end=54842637) | [IPI00562679.1](http://www.ensembl.org/Rattus_norvegicus/geneview?gene=IPI00562679.1) |  |
|  | [ENSRNOG00000042398](http://www.ensembl.org/Rattus_norvegicus/Gene/Summary?db=core;g=ENSRNOG00000042398) | [14](http://www.ensembl.org/Rattus_norvegicus/mapview?chr=14) | [56733153](http://www.ensembl.org/Rattus_norvegicus/contigview?chr=14&vc_start=56733153&vc_end=56733561) | [56733561](http://www.ensembl.org/Rattus_norvegicus/contigview?chr=14&vc_start=56733153&vc_end=56733561) |  |  |
|  | [ENSRNOG00000014430](http://www.ensembl.org/Rattus_norvegicus/Gene/Summary?db=core;g=ENSRNOG00000014430) | [14](http://www.ensembl.org/Rattus_norvegicus/mapview?chr=14) | [59286251](http://www.ensembl.org/Rattus_norvegicus/contigview?chr=14&vc_start=59286251&vc_end=59287934) | [59287934](http://www.ensembl.org/Rattus_norvegicus/contigview?chr=14&vc_start=59286251&vc_end=59287934) |  |  |
|  | [ENSRNOG00000043124](http://www.ensembl.org/Rattus_norvegicus/Gene/Summary?db=core;g=ENSRNOG00000043124) | [14](http://www.ensembl.org/Rattus_norvegicus/mapview?chr=14) | [62052510](http://www.ensembl.org/Rattus_norvegicus/contigview?chr=14&vc_start=62052510&vc_end=62057205) | [62057205](http://www.ensembl.org/Rattus_norvegicus/contigview?chr=14&vc_start=62052510&vc_end=62057205) | [CCKAR_RAT](http://www.ensembl.org/Rattus_norvegicus/geneview?gene=CCKAR_RAT) | **Cholecystokinin receptor type A** |
|  | [ENSRNOG00000004932](http://www.ensembl.org/Rattus_norvegicus/Gene/Summary?db=core;g=ENSRNOG00000004932) | [14](http://www.ensembl.org/Rattus_norvegicus/mapview?chr=14) | [62523780](http://www.ensembl.org/Rattus_norvegicus/contigview?chr=14&vc_start=62523780&vc_end=62630809) | [62630809](http://www.ensembl.org/Rattus_norvegicus/contigview?chr=14&vc_start=62523780&vc_end=62630809) | [RGD1562860](http://www.ensembl.org/Rattus_norvegicus/geneview?gene=RGD1562860) |  |
|  | [ENSRNOG00000027791](http://www.ensembl.org/Rattus_norvegicus/Gene/Summary?db=core;g=ENSRNOG00000027791) | [14](http://www.ensembl.org/Rattus_norvegicus/mapview?chr=14) | [55352566](http://www.ensembl.org/Rattus_norvegicus/contigview?chr=14&vc_start=55352566&vc_end=55353001) | [55353001](http://www.ensembl.org/Rattus_norvegicus/contigview?chr=14&vc_start=55352566&vc_end=55353001) | [CX7A2_RAT](http://www.ensembl.org/Rattus_norvegicus/geneview?gene=CX7A2_RAT) | Cytochrome c oxidase polypeptide 7A2, mitochondrial Precursor |
|  | [ENSRNOG00000028963](http://www.ensembl.org/Rattus_norvegicus/Gene/Summary?db=core;g=ENSRNOG00000028963) | [14](http://www.ensembl.org/Rattus_norvegicus/mapview?chr=14) | [61301643](http://www.ensembl.org/Rattus_norvegicus/contigview?chr=14&vc_start=61301643&vc_end=61302107) | [61302107](http://www.ensembl.org/Rattus_norvegicus/contigview?chr=14&vc_start=61301643&vc_end=61302107) | [RGD1562755](http://www.ensembl.org/Rattus_norvegicus/geneview?gene=RGD1562755) |  |
|  | [ENSRNOG00000042001](http://www.ensembl.org/Rattus_norvegicus/Gene/Summary?db=core;g=ENSRNOG00000042001) | [14](http://www.ensembl.org/Rattus_norvegicus/mapview?chr=14) | [52366309](http://www.ensembl.org/Rattus_norvegicus/contigview?chr=14&vc_start=52366309&vc_end=52366597) | [52366597](http://www.ensembl.org/Rattus_norvegicus/contigview?chr=14&vc_start=52366309&vc_end=52366597) | [SRP_euk_arch](http://www.ensembl.org/Rattus_norvegicus/geneview?gene=SRP_euk_arch) | Eukaryotic type signal recognition particle RNA |
|  | [ENSRNOG00000041646](http://www.ensembl.org/Rattus_norvegicus/Gene/Summary?db=core;g=ENSRNOG00000041646) | [14](http://www.ensembl.org/Rattus_norvegicus/mapview?chr=14) | [61711680](http://www.ensembl.org/Rattus_norvegicus/contigview?chr=14&vc_start=61711680&vc_end=61711868) | [61711868](http://www.ensembl.org/Rattus_norvegicus/contigview?chr=14&vc_start=61711680&vc_end=61711868) | [U2](http://www.ensembl.org/Rattus_norvegicus/geneview?gene=U2) | U2 spliceosomal RNA |
|  | [ENSRNOG00000041479](http://www.ensembl.org/Rattus_norvegicus/Gene/Summary?db=core;g=ENSRNOG00000041479) | [14](http://www.ensembl.org/Rattus_norvegicus/mapview?chr=14) | [60849570](http://www.ensembl.org/Rattus_norvegicus/contigview?chr=14&vc_start=60849570&vc_end=60849638) | [60849638](http://www.ensembl.org/Rattus_norvegicus/contigview?chr=14&vc_start=60849570&vc_end=60849638) |  |  |
|  | [ENSRNOG00000034366](http://www.ensembl.org/Rattus_norvegicus/Gene/Summary?db=core;g=ENSRNOG00000034366) | [14](http://www.ensembl.org/Rattus_norvegicus/mapview?chr=14) | [61910452](http://www.ensembl.org/Rattus_norvegicus/contigview?chr=14&vc_start=61910452&vc_end=61910556) | [61910556](http://www.ensembl.org/Rattus_norvegicus/contigview?chr=14&vc_start=61910452&vc_end=61910556) | [5S_rRNA](http://www.ensembl.org/Rattus_norvegicus/geneview?gene=5S_rRNA) | 5S ribosomal RNA |
|  | [ENSRNOG00000034707](http://www.ensembl.org/Rattus_norvegicus/Gene/Summary?db=core;g=ENSRNOG00000034707) | [14](http://www.ensembl.org/Rattus_norvegicus/mapview?chr=14) | [61018826](http://www.ensembl.org/Rattus_norvegicus/contigview?chr=14&vc_start=61018826&vc_end=61018929) | [61018929](http://www.ensembl.org/Rattus_norvegicus/contigview?chr=14&vc_start=61018826&vc_end=61018929) | [U6](http://www.ensembl.org/Rattus_norvegicus/geneview?gene=U6) | U6 spliceosomal RNA |
|  | [ENSRNOG00000041655](http://www.ensembl.org/Rattus_norvegicus/Gene/Summary?db=core;g=ENSRNOG00000041655) | [14](http://www.ensembl.org/Rattus_norvegicus/mapview?chr=14) | [58310271](http://www.ensembl.org/Rattus_norvegicus/contigview?chr=14&vc_start=58310271&vc_end=58310458) | [58310458](http://www.ensembl.org/Rattus_norvegicus/contigview?chr=14&vc_start=58310271&vc_end=58310458) | [U2](http://www.ensembl.org/Rattus_norvegicus/geneview?gene=U2) | U2 spliceosomal RNA |
|  | [ENSRNOG00000043696](http://www.ensembl.org/Rattus_norvegicus/Gene/Summary?db=core;g=ENSRNOG00000043696) | [14](http://www.ensembl.org/Rattus_norvegicus/mapview?chr=14) | [52758505](http://www.ensembl.org/Rattus_norvegicus/contigview?chr=14&vc_start=52758505&vc_end=52758616) | [52758616](http://www.ensembl.org/Rattus_norvegicus/contigview?chr=14&vc_start=52758505&vc_end=52758616) |  |  |
|  | [ENSRNOG00000034513](http://www.ensembl.org/Rattus_norvegicus/Gene/Summary?db=core;g=ENSRNOG00000034513) | [14](http://www.ensembl.org/Rattus_norvegicus/mapview?chr=14) | [62395295](http://www.ensembl.org/Rattus_norvegicus/contigview?chr=14&vc_start=62395295&vc_end=62395399) | [62395399](http://www.ensembl.org/Rattus_norvegicus/contigview?chr=14&vc_start=62395295&vc_end=62395399) | [5S_rRNA](http://www.ensembl.org/Rattus_norvegicus/geneview?gene=5S_rRNA) | 5S ribosomal RNA |
|  | [ENSRNOG00000040684](http://www.ensembl.org/Rattus_norvegicus/Gene/Summary?db=core;g=ENSRNOG00000040684) | [14](http://www.ensembl.org/Rattus_norvegicus/mapview?chr=14) | [50860576](http://www.ensembl.org/Rattus_norvegicus/contigview?chr=14&vc_start=50860576&vc_end=50860706) | [50860706](http://www.ensembl.org/Rattus_norvegicus/contigview?chr=14&vc_start=50860576&vc_end=50860706) | [SNORA17](http://www.ensembl.org/Rattus_norvegicus/geneview?gene=SNORA17) | Small nucleolar RNA SNORA17 |
| **Parameters - SERT-/-** | **Ensembl Gene ID** | **Chr.** | **Gene Start** | **Gene End** | **Gene Name** | **Description** |
| Cocaine-induced locomotor | [ENSRNOG00000042411](http://www.ensembl.org/Rattus_norvegicus/Gene/Summary?db=core;g=ENSRNOG00000042411) | [5](http://www.ensembl.org/Rattus_norvegicus/mapview?chr=5) | [151274641](http://www.ensembl.org/Rattus_norvegicus/contigview?chr=5&vc_start=151274641&vc_end=151288004) | [151288004](http://www.ensembl.org/Rattus_norvegicus/contigview?chr=5&vc_start=151274641&vc_end=151288004) | [NP_112369.1](http://www.ensembl.org/Rattus_norvegicus/geneview?gene=NP_112369.1) | ribosomal protein S6 kinase polypeptide 1 |
| activity 3-120 min | [ENSRNOG00000006137](http://www.ensembl.org/Rattus_norvegicus/Gene/Summary?db=core;g=ENSRNOG00000006137) | [5](http://www.ensembl.org/Rattus_norvegicus/mapview?chr=5) | [151325661](http://www.ensembl.org/Rattus_norvegicus/contigview?chr=5&vc_start=151325661&vc_end=151398647) | [151398647](http://www.ensembl.org/Rattus_norvegicus/contigview?chr=5&vc_start=151325661&vc_end=151398647) | [NP_001100105.1](http://www.ensembl.org/Rattus_norvegicus/geneview?gene=NP_001100105.1) | AT rich interactive domain 1A (SWI-like) |
|  | [ENSRNOG00000000121](http://www.ensembl.org/Rattus_norvegicus/Gene/Summary?db=core;g=ENSRNOG00000000121) | [5](http://www.ensembl.org/Rattus_norvegicus/mapview?chr=5) | [151404858](http://www.ensembl.org/Rattus_norvegicus/contigview?chr=5&vc_start=151404858&vc_end=151416744) | [151416744](http://www.ensembl.org/Rattus_norvegicus/contigview?chr=5&vc_start=151404858&vc_end=151416744) | [PIGV_RAT](http://www.ensembl.org/Rattus_norvegicus/geneview?gene=PIGV_RAT) | GPI mannosyltransferase 2 |
|  | [ENSRNOG00000007021](http://www.ensembl.org/Rattus_norvegicus/Gene/Summary?db=core;g=ENSRNOG00000007021) | [5](http://www.ensembl.org/Rattus_norvegicus/mapview?chr=5) | [151447114](http://www.ensembl.org/Rattus_norvegicus/contigview?chr=5&vc_start=151447114&vc_end=151472403) | [151472403](http://www.ensembl.org/Rattus_norvegicus/contigview?chr=5&vc_start=151447114&vc_end=151472403) | [ZDH18_RAT](http://www.ensembl.org/Rattus_norvegicus/geneview?gene=ZDH18_RAT) | Palmitoyltransferase ZDHHC18 |
|  | [ENSRNOG00000007399](http://www.ensembl.org/Rattus_norvegicus/Gene/Summary?db=core;g=ENSRNOG00000007399) | [5](http://www.ensembl.org/Rattus_norvegicus/mapview?chr=5) | [151535900](http://www.ensembl.org/Rattus_norvegicus/contigview?chr=5&vc_start=151535900&vc_end=151550041) | [151550041](http://www.ensembl.org/Rattus_norvegicus/contigview?chr=5&vc_start=151535900&vc_end=151550041) | [NUDC_RAT](http://www.ensembl.org/Rattus_norvegicus/geneview?gene=NUDC_RAT) | Nuclear migration protein nudC |
|  | [ENSRNOG00000026561](http://www.ensembl.org/Rattus_norvegicus/Gene/Summary?db=core;g=ENSRNOG00000026561) | [5](http://www.ensembl.org/Rattus_norvegicus/mapview?chr=5) | [151555174](http://www.ensembl.org/Rattus_norvegicus/contigview?chr=5&vc_start=151555174&vc_end=151555714) | [151555714](http://www.ensembl.org/Rattus_norvegicus/contigview?chr=5&vc_start=151555174&vc_end=151555714) |  |  |
|  | [ENSRNOG00000026483](http://www.ensembl.org/Rattus_norvegicus/Gene/Summary?db=core;g=ENSRNOG00000026483) | [5](http://www.ensembl.org/Rattus_norvegicus/mapview?chr=5) | [151595273](http://www.ensembl.org/Rattus_norvegicus/contigview?chr=5&vc_start=151595273&vc_end=151596015) | [151596015](http://www.ensembl.org/Rattus_norvegicus/contigview?chr=5&vc_start=151595273&vc_end=151596015) | [LOC688990](http://www.ensembl.org/Rattus_norvegicus/geneview?gene=LOC688990) |  |
|  | [ENSRNOG00000008377](http://www.ensembl.org/Rattus_norvegicus/Gene/Summary?db=core;g=ENSRNOG00000008377) | [5](http://www.ensembl.org/Rattus_norvegicus/mapview?chr=5) | [151784746](http://www.ensembl.org/Rattus_norvegicus/contigview?chr=5&vc_start=151784746&vc_end=151834368) | [151834368](http://www.ensembl.org/Rattus_norvegicus/contigview?chr=5&vc_start=151784746&vc_end=151834368) | [NP_001101378.1](http://www.ensembl.org/Rattus_norvegicus/geneview?gene=NP_001101378.1) | WD and tetratricopeptide repeats 1 |
|  | [ENSRNOG00000008564](http://www.ensembl.org/Rattus_norvegicus/Gene/Summary?db=core;g=ENSRNOG00000008564) | [5](http://www.ensembl.org/Rattus_norvegicus/mapview?chr=5) | [151848197](http://www.ensembl.org/Rattus_norvegicus/contigview?chr=5&vc_start=151848197&vc_end=151859984) | [151859984](http://www.ensembl.org/Rattus_norvegicus/contigview?chr=5&vc_start=151848197&vc_end=151859984) | [NP_001107252.1](http://www.ensembl.org/Rattus_norvegicus/geneview?gene=NP_001107252.1) | transmembrane protein 222 |
|  | [ENSRNOG00000008600](http://www.ensembl.org/Rattus_norvegicus/Gene/Summary?db=core;g=ENSRNOG00000008600) | [5](http://www.ensembl.org/Rattus_norvegicus/mapview?chr=5) | [151862910](http://www.ensembl.org/Rattus_norvegicus/contigview?chr=5&vc_start=151862910&vc_end=151873304) | [151873304](http://www.ensembl.org/Rattus_norvegicus/contigview?chr=5&vc_start=151862910&vc_end=151873304) | [NP_001020822.1](http://www.ensembl.org/Rattus_norvegicus/geneview?gene=NP_001020822.1) | synaptotagmin-like 1 |
|  | [ENSRNOG00000011254](http://www.ensembl.org/Rattus_norvegicus/Gene/Summary?db=core;g=ENSRNOG00000011254) | [5](http://www.ensembl.org/Rattus_norvegicus/mapview?chr=5) | [152218077](http://www.ensembl.org/Rattus_norvegicus/contigview?chr=5&vc_start=152218077&vc_end=152246849) | [152246849](http://www.ensembl.org/Rattus_norvegicus/contigview?chr=5&vc_start=152218077&vc_end=152246849) | [NP_001102156.1](http://www.ensembl.org/Rattus_norvegicus/geneview?gene=NP_001102156.1) | hypothetical protein LOC362618 |
|  | [ENSRNOG00000037202](http://www.ensembl.org/Rattus_norvegicus/Gene/Summary?db=core;g=ENSRNOG00000037202) | [5](http://www.ensembl.org/Rattus_norvegicus/mapview?chr=5) | [152218267](http://www.ensembl.org/Rattus_norvegicus/contigview?chr=5&vc_start=152218267&vc_end=152452354) | [152452354](http://www.ensembl.org/Rattus_norvegicus/contigview?chr=5&vc_start=152218267&vc_end=152452354) | [IPI00779434.1](http://www.ensembl.org/Rattus_norvegicus/geneview?gene=IPI00779434.1) |  |
|  | [ENSRNOG00000011804](http://www.ensembl.org/Rattus_norvegicus/Gene/Summary?db=core;g=ENSRNOG00000011804) | [5](http://www.ensembl.org/Rattus_norvegicus/mapview?chr=5) | [152261969](http://www.ensembl.org/Rattus_norvegicus/contigview?chr=5&vc_start=152261969&vc_end=152290210) | [152290210](http://www.ensembl.org/Rattus_norvegicus/contigview?chr=5&vc_start=152261969&vc_end=152290210) | [NP_075228.2](http://www.ensembl.org/Rattus_norvegicus/geneview?gene=NP_075228.2) | syntaxin 12 |
|  | [ENSRNOG00000012935](http://www.ensembl.org/Rattus_norvegicus/Gene/Summary?db=core;g=ENSRNOG00000012935) | [5](http://www.ensembl.org/Rattus_norvegicus/mapview?chr=5) | [152305936](http://www.ensembl.org/Rattus_norvegicus/contigview?chr=5&vc_start=152305936&vc_end=152323075) | [152323075](http://www.ensembl.org/Rattus_norvegicus/contigview?chr=5&vc_start=152305936&vc_end=152323075) | [NP_001101381.1](http://www.ensembl.org/Rattus_norvegicus/geneview?gene=NP_001101381.1) | protein phosphatase 1, regulatory (inhibitor) subunit 8 |
|  | [ENSRNOG00000012965](http://www.ensembl.org/Rattus_norvegicus/Gene/Summary?db=core;g=ENSRNOG00000012965) | [5](http://www.ensembl.org/Rattus_norvegicus/mapview?chr=5) | [152349541](http://www.ensembl.org/Rattus_norvegicus/contigview?chr=5&vc_start=152349541&vc_end=152364664) | [152364664](http://www.ensembl.org/Rattus_norvegicus/contigview?chr=5&vc_start=152349541&vc_end=152364664) | [RGD1561016](http://www.ensembl.org/Rattus_norvegicus/geneview?gene=RGD1561016) |  |
|  | [ENSRNOG00000042326](http://www.ensembl.org/Rattus_norvegicus/Gene/Summary?db=core;g=ENSRNOG00000042326) | [5](http://www.ensembl.org/Rattus_norvegicus/mapview?chr=5) | [152390454](http://www.ensembl.org/Rattus_norvegicus/contigview?chr=5&vc_start=152390454&vc_end=152415334) | [152415334](http://www.ensembl.org/Rattus_norvegicus/contigview?chr=5&vc_start=152390454&vc_end=152415334) | [NP_001020908.1](http://www.ensembl.org/Rattus_norvegicus/geneview?gene=NP_001020908.1) | sphingomyelin phosphodiesterase, acid-like 3B |
|  | [ENSRNOG00000024312](http://www.ensembl.org/Rattus_norvegicus/Gene/Summary?db=core;g=ENSRNOG00000024312) | [5](http://www.ensembl.org/Rattus_norvegicus/mapview?chr=5) | [152415634](http://www.ensembl.org/Rattus_norvegicus/contigview?chr=5&vc_start=152415634&vc_end=152422706) | [152422706](http://www.ensembl.org/Rattus_norvegicus/contigview?chr=5&vc_start=152415634&vc_end=152422706) | [NP_001012099.1](http://www.ensembl.org/Rattus_norvegicus/geneview?gene=NP_001012099.1) | XK, Kell blood group complex subunit-related family, member 8 |
|  | [ENSRNOG00000013300](http://www.ensembl.org/Rattus_norvegicus/Gene/Summary?db=core;g=ENSRNOG00000013300) | [5](http://www.ensembl.org/Rattus_norvegicus/mapview?chr=5) | [152618307](http://www.ensembl.org/Rattus_norvegicus/contigview?chr=5&vc_start=152618307&vc_end=152622024) | [152622024](http://www.ensembl.org/Rattus_norvegicus/contigview?chr=5&vc_start=152618307&vc_end=152622024) | [ATIF1_RAT](http://www.ensembl.org/Rattus_norvegicus/geneview?gene=ATIF1_RAT) | ATPase inhibitor, mitochondrial Precursor |
|  | [ENSRNOG00000042785](http://www.ensembl.org/Rattus_norvegicus/Gene/Summary?db=core;g=ENSRNOG00000042785) | [5](http://www.ensembl.org/Rattus_norvegicus/mapview?chr=5) | [152639951](http://www.ensembl.org/Rattus_norvegicus/contigview?chr=5&vc_start=152639951&vc_end=152651043) | [152651043](http://www.ensembl.org/Rattus_norvegicus/contigview?chr=5&vc_start=152639951&vc_end=152651043) | [NP_001102828.1](http://www.ensembl.org/Rattus_norvegicus/geneview?gene=NP_001102828.1) | hypothetical protein LOC502988 |
|  | [ENSRNOG00000043343](http://www.ensembl.org/Rattus_norvegicus/Gene/Summary?db=core;g=ENSRNOG00000043343) | [5](http://www.ensembl.org/Rattus_norvegicus/mapview?chr=5) | [152801084](http://www.ensembl.org/Rattus_norvegicus/contigview?chr=5&vc_start=152801084&vc_end=152806793) | [152806793](http://www.ensembl.org/Rattus_norvegicus/contigview?chr=5&vc_start=152801084&vc_end=152806793) | [IPI00369116.3](http://www.ensembl.org/Rattus_norvegicus/geneview?gene=IPI00369116.3) |  |
|  | [ENSRNOG00000023423](http://www.ensembl.org/Rattus_norvegicus/Gene/Summary?db=core;g=ENSRNOG00000023423) | [5](http://www.ensembl.org/Rattus_norvegicus/mapview?chr=5) | [152821172](http://www.ensembl.org/Rattus_norvegicus/contigview?chr=5&vc_start=152821172&vc_end=152841613) | [152841613](http://www.ensembl.org/Rattus_norvegicus/contigview?chr=5&vc_start=152821172&vc_end=152841613) | [Aim1l](http://www.ensembl.org/Rattus_norvegicus/geneview?gene=Aim1l) |  |
|  | [ENSRNOG00000015476](http://www.ensembl.org/Rattus_norvegicus/Gene/Summary?db=core;g=ENSRNOG00000015476) | [5](http://www.ensembl.org/Rattus_norvegicus/mapview?chr=5) | [152853129](http://www.ensembl.org/Rattus_norvegicus/contigview?chr=5&vc_start=152853129&vc_end=152876717) | [152876717](http://www.ensembl.org/Rattus_norvegicus/contigview?chr=5&vc_start=152853129&vc_end=152876717) | [UBX11_RAT](http://www.ensembl.org/Rattus_norvegicus/geneview?gene=UBX11_RAT) | UBX domain-containing protein 11 |
|  | [ENSRNOG00000031998](http://www.ensembl.org/Rattus_norvegicus/Gene/Summary?db=core;g=ENSRNOG00000031998) | [5](http://www.ensembl.org/Rattus_norvegicus/mapview?chr=5) | [152994321](http://www.ensembl.org/Rattus_norvegicus/contigview?chr=5&vc_start=152994321&vc_end=153146701) | [153146701](http://www.ensembl.org/Rattus_norvegicus/contigview?chr=5&vc_start=152994321&vc_end=153146701) | [IPI00563115.2](http://www.ensembl.org/Rattus_norvegicus/geneview?gene=IPI00563115.2) |  |
|  | [ENSRNOG00000016543](http://www.ensembl.org/Rattus_norvegicus/Gene/Summary?db=core;g=ENSRNOG00000016543) | [5](http://www.ensembl.org/Rattus_norvegicus/mapview?chr=5) | [153059582](http://www.ensembl.org/Rattus_norvegicus/contigview?chr=5&vc_start=153059582&vc_end=153073887) | [153073887](http://www.ensembl.org/Rattus_norvegicus/contigview?chr=5&vc_start=153059582&vc_end=153073887) | [TRI63_RAT](http://www.ensembl.org/Rattus_norvegicus/geneview?gene=TRI63_RAT) | E3 ubiquitin-protein ligase TRIM63 |
|  | [ENSRNOG00000016650](http://www.ensembl.org/Rattus_norvegicus/Gene/Summary?db=core;g=ENSRNOG00000016650) | [5](http://www.ensembl.org/Rattus_norvegicus/mapview?chr=5) | [153086346](http://www.ensembl.org/Rattus_norvegicus/contigview?chr=5&vc_start=153086346&vc_end=153098568) | [153098568](http://www.ensembl.org/Rattus_norvegicus/contigview?chr=5&vc_start=153086346&vc_end=153098568) | [ZNT2_RAT](http://www.ensembl.org/Rattus_norvegicus/geneview?gene=ZNT2_RAT) | Zinc transporter 2 (ZnT-2)(Solute carrier family 30 member 2) |
|  | [ENSRNOG00000022288](http://www.ensembl.org/Rattus_norvegicus/Gene/Summary?db=core;g=ENSRNOG00000022288) | [5](http://www.ensembl.org/Rattus_norvegicus/mapview?chr=5) | [153157638](http://www.ensembl.org/Rattus_norvegicus/contigview?chr=5&vc_start=153157638&vc_end=153178179) | [153178179](http://www.ensembl.org/Rattus_norvegicus/contigview?chr=5&vc_start=153157638&vc_end=153178179) | [PAFA2_RAT](http://www.ensembl.org/Rattus_norvegicus/geneview?gene=PAFA2_RAT) | Platelet-activating factor acetylhydrolase 2, cytoplasmic |
|  | [ENSRNOG00000016810](http://www.ensembl.org/Rattus_norvegicus/Gene/Summary?db=core;g=ENSRNOG00000016810) | [5](http://www.ensembl.org/Rattus_norvegicus/mapview?chr=5) | [153226409](http://www.ensembl.org/Rattus_norvegicus/contigview?chr=5&vc_start=153226409&vc_end=153232084) | [153232084](http://www.ensembl.org/Rattus_norvegicus/contigview?chr=5&vc_start=153226409&vc_end=153232084) | [STMN1_RAT](http://www.ensembl.org/Rattus_norvegicus/geneview?gene=STMN1_RAT) | Stathmin (Phosphoprotein p19) |
|  | [ENSRNOG00000022030](http://www.ensembl.org/Rattus_norvegicus/Gene/Summary?db=core;g=ENSRNOG00000022030) | [5](http://www.ensembl.org/Rattus_norvegicus/mapview?chr=5) | [153267331](http://www.ensembl.org/Rattus_norvegicus/contigview?chr=5&vc_start=153267331&vc_end=153280029) | [153280029](http://www.ensembl.org/Rattus_norvegicus/contigview?chr=5&vc_start=153267331&vc_end=153280029) | [LOC689656](http://www.ensembl.org/Rattus_norvegicus/geneview?gene=LOC689656) |  |
|  | [ENSRNOG00000017130](http://www.ensembl.org/Rattus_norvegicus/Gene/Summary?db=core;g=ENSRNOG00000017130) | [5](http://www.ensembl.org/Rattus_norvegicus/mapview?chr=5) | [153638642](http://www.ensembl.org/Rattus_norvegicus/contigview?chr=5&vc_start=153638642&vc_end=153672531) | [153672531](http://www.ensembl.org/Rattus_norvegicus/contigview?chr=5&vc_start=153638642&vc_end=153672531) | [NP_071950.1](http://www.ensembl.org/Rattus_norvegicus/geneview?gene=NP_071950.1) | Rh blood group D antigen |
|  | [ENSRNOG00000037180](http://www.ensembl.org/Rattus_norvegicus/Gene/Summary?db=core;g=ENSRNOG00000037180) | [5](http://www.ensembl.org/Rattus_norvegicus/mapview?chr=5) | [153665494](http://www.ensembl.org/Rattus_norvegicus/contigview?chr=5&vc_start=153665494&vc_end=153849512) | [153849512](http://www.ensembl.org/Rattus_norvegicus/contigview?chr=5&vc_start=153665494&vc_end=153849512) | [IPI00780402.1](http://www.ensembl.org/Rattus_norvegicus/geneview?gene=IPI00780402.1) |  |
|  | [ENSRNOG00000017309](http://www.ensembl.org/Rattus_norvegicus/Gene/Summary?db=core;g=ENSRNOG00000017309) | [5](http://www.ensembl.org/Rattus_norvegicus/mapview?chr=5) | [153698998](http://www.ensembl.org/Rattus_norvegicus/contigview?chr=5&vc_start=153698998&vc_end=153702609) | [153702609](http://www.ensembl.org/Rattus_norvegicus/contigview?chr=5&vc_start=153698998&vc_end=153702609) | [CA063_RAT](http://www.ensembl.org/Rattus_norvegicus/geneview?gene=CA063_RAT) | UPF0471 protein C1orf63 homolog |
|  | [ENSRNOG00000017742](http://www.ensembl.org/Rattus_norvegicus/Gene/Summary?db=core;g=ENSRNOG00000017742) | [5](http://www.ensembl.org/Rattus_norvegicus/mapview?chr=5) | [153707652](http://www.ensembl.org/Rattus_norvegicus/contigview?chr=5&vc_start=153707652&vc_end=153715578) | [153715578](http://www.ensembl.org/Rattus_norvegicus/contigview?chr=5&vc_start=153707652&vc_end=153715578) | [SYF2_RAT](http://www.ensembl.org/Rattus_norvegicus/geneview?gene=SYF2_RAT) | Pre-mRNA-splicing factor SYF2 |
|  | [ENSRNOG00000018058](http://www.ensembl.org/Rattus_norvegicus/Gene/Summary?db=core;g=ENSRNOG00000018058) | [5](http://www.ensembl.org/Rattus_norvegicus/mapview?chr=5) | [153950116](http://www.ensembl.org/Rattus_norvegicus/contigview?chr=5&vc_start=153950116&vc_end=153973141) | [153973141](http://www.ensembl.org/Rattus_norvegicus/contigview?chr=5&vc_start=153950116&vc_end=153973141) | [NP_569109.1](http://www.ensembl.org/Rattus_norvegicus/geneview?gene=NP_569109.1) | runt-related transcription factor 3 |
|  | [ENSRNOG00000031448](http://www.ensembl.org/Rattus_norvegicus/Gene/Summary?db=core;g=ENSRNOG00000031448) | [5](http://www.ensembl.org/Rattus_norvegicus/mapview?chr=5) | [154264790](http://www.ensembl.org/Rattus_norvegicus/contigview?chr=5&vc_start=154264790&vc_end=154308314) | [154308314](http://www.ensembl.org/Rattus_norvegicus/contigview?chr=5&vc_start=154264790&vc_end=154308314) | [CA201_RAT](http://www.ensembl.org/Rattus_norvegicus/geneview?gene=CA201_RAT) | UPF0490 protein C1orf201 homolog |
|  | [ENSRNOG00000026676](http://www.ensembl.org/Rattus_norvegicus/Gene/Summary?db=core;g=ENSRNOG00000026676) | [5](http://www.ensembl.org/Rattus_norvegicus/mapview?chr=5) | [154398479](http://www.ensembl.org/Rattus_norvegicus/contigview?chr=5&vc_start=154398479&vc_end=154400790) | [154400790](http://www.ensembl.org/Rattus_norvegicus/contigview?chr=5&vc_start=154398479&vc_end=154400790) | [NP_001019519.1](http://www.ensembl.org/Rattus_norvegicus/geneview?gene=NP_001019519.1) | hypothetical protein LOC500567 |
|  | [ENSRNOG00000033984](http://www.ensembl.org/Rattus_norvegicus/Gene/Summary?db=core;g=ENSRNOG00000033984) | [5](http://www.ensembl.org/Rattus_norvegicus/mapview?chr=5) | [154461469](http://www.ensembl.org/Rattus_norvegicus/contigview?chr=5&vc_start=154461469&vc_end=154480844) | [154480844](http://www.ensembl.org/Rattus_norvegicus/contigview?chr=5&vc_start=154461469&vc_end=154480844) | [Il28ra](http://www.ensembl.org/Rattus_norvegicus/geneview?gene=Il28ra) |  |
|  | [ENSRNOG00000027976](http://www.ensembl.org/Rattus_norvegicus/Gene/Summary?db=core;g=ENSRNOG00000027976) | [5](http://www.ensembl.org/Rattus_norvegicus/mapview?chr=5) | [154502235](http://www.ensembl.org/Rattus_norvegicus/contigview?chr=5&vc_start=154502235&vc_end=154526724) | [154526724](http://www.ensembl.org/Rattus_norvegicus/contigview?chr=5&vc_start=154502235&vc_end=154526724) | [Il22ra1](http://www.ensembl.org/Rattus_norvegicus/geneview?gene=Il22ra1) |  |
|  | [ENSRNOG00000032994](http://www.ensembl.org/Rattus_norvegicus/Gene/Summary?db=core;g=ENSRNOG00000032994) | [5](http://www.ensembl.org/Rattus_norvegicus/mapview?chr=5) | [154544049](http://www.ensembl.org/Rattus_norvegicus/contigview?chr=5&vc_start=154544049&vc_end=154591541) | [154591541](http://www.ensembl.org/Rattus_norvegicus/contigview?chr=5&vc_start=154544049&vc_end=154591541) | [IPI00359371.5](http://www.ensembl.org/Rattus_norvegicus/geneview?gene=IPI00359371.5) |  |
|  | [ENSRNOG00000007992](http://www.ensembl.org/Rattus_norvegicus/Gene/Summary?db=core;g=ENSRNOG00000007992) | [5](http://www.ensembl.org/Rattus_norvegicus/mapview?chr=5) | [154637426](http://www.ensembl.org/Rattus_norvegicus/contigview?chr=5&vc_start=154637426&vc_end=154648795) | [154648795](http://www.ensembl.org/Rattus_norvegicus/contigview?chr=5&vc_start=154637426&vc_end=154648795) | [NP_001020909.1](http://www.ensembl.org/Rattus_norvegicus/geneview?gene=NP_001020909.1) | FUS interacting protein (serine-arginine rich) 1 |
|  | [ENSRNOG00000009260](http://www.ensembl.org/Rattus_norvegicus/Gene/Summary?db=core;g=ENSRNOG00000009260) | [5](http://www.ensembl.org/Rattus_norvegicus/mapview?chr=5) | [154699523](http://www.ensembl.org/Rattus_norvegicus/contigview?chr=5&vc_start=154699523&vc_end=154702057) | [154702057](http://www.ensembl.org/Rattus_norvegicus/contigview?chr=5&vc_start=154699523&vc_end=154702057) | [CNR2_RAT](http://www.ensembl.org/Rattus_norvegicus/geneview?gene=CNR2_RAT) | **Cannabinoid receptor 2** |
|  | [ENSRNOG00000009325](http://www.ensembl.org/Rattus_norvegicus/Gene/Summary?db=core;g=ENSRNOG00000009325) | [5](http://www.ensembl.org/Rattus_norvegicus/mapview?chr=5) | [154703722](http://www.ensembl.org/Rattus_norvegicus/contigview?chr=5&vc_start=154703722&vc_end=154720970) | [154720970](http://www.ensembl.org/Rattus_norvegicus/contigview?chr=5&vc_start=154703722&vc_end=154720970) | [FUCO_RAT](http://www.ensembl.org/Rattus_norvegicus/geneview?gene=FUCO_RAT) | Tissue alpha-L-fucosidase Precursor |
|  | [ENSRNOG00000009422](http://www.ensembl.org/Rattus_norvegicus/Gene/Summary?db=core;g=ENSRNOG00000009422) | [5](http://www.ensembl.org/Rattus_norvegicus/mapview?chr=5) | [154730230](http://www.ensembl.org/Rattus_norvegicus/contigview?chr=5&vc_start=154730230&vc_end=154743974) | [154743974](http://www.ensembl.org/Rattus_norvegicus/contigview?chr=5&vc_start=154730230&vc_end=154743974) | [HMGCL_RAT](http://www.ensembl.org/Rattus_norvegicus/geneview?gene=HMGCL_RAT) | Hydroxymethylglutaryl-CoA lyase, mitochondrial Precursor |
|  | [ENSRNOG00000009712](http://www.ensembl.org/Rattus_norvegicus/Gene/Summary?db=core;g=ENSRNOG00000009712) | [5](http://www.ensembl.org/Rattus_norvegicus/mapview?chr=5) | [154745853](http://www.ensembl.org/Rattus_norvegicus/contigview?chr=5&vc_start=154745853&vc_end=154750355) | [154750355](http://www.ensembl.org/Rattus_norvegicus/contigview?chr=5&vc_start=154745853&vc_end=154750355) | [GALE_RAT](http://www.ensembl.org/Rattus_norvegicus/geneview?gene=GALE_RAT) | UDP-glucose 4-epimerase |
|  | [ENSRNOG00000026124](http://www.ensembl.org/Rattus_norvegicus/Gene/Summary?db=core;g=ENSRNOG00000026124) | [5](http://www.ensembl.org/Rattus_norvegicus/mapview?chr=5) | [154934138](http://www.ensembl.org/Rattus_norvegicus/contigview?chr=5&vc_start=154934138&vc_end=154935703) | [154935703](http://www.ensembl.org/Rattus_norvegicus/contigview?chr=5&vc_start=154934138&vc_end=154935703) | [ID3_RAT](http://www.ensembl.org/Rattus_norvegicus/geneview?gene=ID3_RAT) | DNA-binding protein inhibitor ID-3 (Inhibitor of DNA binding 3) |
|  | [ENSRNOG00000011794](http://www.ensembl.org/Rattus_norvegicus/Gene/Summary?db=core;g=ENSRNOG00000011794) | [5](http://www.ensembl.org/Rattus_norvegicus/mapview?chr=5) | [154979581](http://www.ensembl.org/Rattus_norvegicus/contigview?chr=5&vc_start=154979581&vc_end=155019310) | [155019310](http://www.ensembl.org/Rattus_norvegicus/contigview?chr=5&vc_start=154979581&vc_end=155019310) | [NP_001015008.1](http://www.ensembl.org/Rattus_norvegicus/geneview?gene=NP_001015008.1) | transcription elongation factor A (SII), 3 |
|  | [ENSRNOG00000022561](http://www.ensembl.org/Rattus_norvegicus/Gene/Summary?db=core;g=ENSRNOG00000022561) | [5](http://www.ensembl.org/Rattus_norvegicus/mapview?chr=5) | [155031667](http://www.ensembl.org/Rattus_norvegicus/contigview?chr=5&vc_start=155031667&vc_end=155037313) | [155037313](http://www.ensembl.org/Rattus_norvegicus/contigview?chr=5&vc_start=155031667&vc_end=155037313) | [NP_001100161.1](http://www.ensembl.org/Rattus_norvegicus/geneview?gene=NP_001100161.1) | zinc finger protein 46 |
|  | [ENSRNOG00000011910](http://www.ensembl.org/Rattus_norvegicus/Gene/Summary?db=core;g=ENSRNOG00000011910) | [5](http://www.ensembl.org/Rattus_norvegicus/mapview?chr=5) | [155053555](http://www.ensembl.org/Rattus_norvegicus/contigview?chr=5&vc_start=155053555&vc_end=155086481) | [155086481](http://www.ensembl.org/Rattus_norvegicus/contigview?chr=5&vc_start=155053555&vc_end=155086481) | [NP_783193.2](http://www.ensembl.org/Rattus_norvegicus/geneview?gene=NP_783193.2) | heterogeneous nuclear ribonucleoprotein R |
|  | [ENSRNOG00000022457](http://www.ensembl.org/Rattus_norvegicus/Gene/Summary?db=core;g=ENSRNOG00000022457) | [5](http://www.ensembl.org/Rattus_norvegicus/mapview?chr=5) | [155208891](http://www.ensembl.org/Rattus_norvegicus/contigview?chr=5&vc_start=155208891&vc_end=155209365) | [155209365](http://www.ensembl.org/Rattus_norvegicus/contigview?chr=5&vc_start=155208891&vc_end=155209365) |  |  |
|  | [ENSRNOG00000029467](http://www.ensembl.org/Rattus_norvegicus/Gene/Summary?db=core;g=ENSRNOG00000029467) | [5](http://www.ensembl.org/Rattus_norvegicus/mapview?chr=5) | [155258920](http://www.ensembl.org/Rattus_norvegicus/contigview?chr=5&vc_start=155258920&vc_end=155259785) | [155259785](http://www.ensembl.org/Rattus_norvegicus/contigview?chr=5&vc_start=155258920&vc_end=155259785) | [IPI00558257.2](http://www.ensembl.org/Rattus_norvegicus/geneview?gene=IPI00558257.2) |  |
|  | [ENSRNOG00000022402](http://www.ensembl.org/Rattus_norvegicus/Gene/Summary?db=core;g=ENSRNOG00000022402) | [5](http://www.ensembl.org/Rattus_norvegicus/mapview?chr=5) | [155289215](http://www.ensembl.org/Rattus_norvegicus/contigview?chr=5&vc_start=155289215&vc_end=155297920) | [155297920](http://www.ensembl.org/Rattus_norvegicus/contigview?chr=5&vc_start=155289215&vc_end=155297920) | [LUZP1_RAT](http://www.ensembl.org/Rattus_norvegicus/geneview?gene=LUZP1_RAT) | Leucine zipper protein 1 |
|  | [ENSRNOG00000022257](http://www.ensembl.org/Rattus_norvegicus/Gene/Summary?db=core;g=ENSRNOG00000022257) | [5](http://www.ensembl.org/Rattus_norvegicus/mapview?chr=5) | [155370254](http://www.ensembl.org/Rattus_norvegicus/contigview?chr=5&vc_start=155370254&vc_end=155375024) | [155375024](http://www.ensembl.org/Rattus_norvegicus/contigview?chr=5&vc_start=155370254&vc_end=155375024) | [RGD1311870](http://www.ensembl.org/Rattus_norvegicus/geneview?gene=RGD1311870) |  |
|  | [ENSRNOG00000013166](http://www.ensembl.org/Rattus_norvegicus/Gene/Summary?db=core;g=ENSRNOG00000013166) | [5](http://www.ensembl.org/Rattus_norvegicus/mapview?chr=5) | [156064350](http://www.ensembl.org/Rattus_norvegicus/contigview?chr=5&vc_start=156064350&vc_end=156083190) | [156083190](http://www.ensembl.org/Rattus_norvegicus/contigview?chr=5&vc_start=156064350&vc_end=156083190) | [WNT4_RAT](http://www.ensembl.org/Rattus_norvegicus/geneview?gene=WNT4_RAT) | Protein Wnt-4 Precursor |
|  | [ENSRNOG00000013566](http://www.ensembl.org/Rattus_norvegicus/Gene/Summary?db=core;g=ENSRNOG00000013566) | [5](http://www.ensembl.org/Rattus_norvegicus/mapview?chr=5) | [156209107](http://www.ensembl.org/Rattus_norvegicus/contigview?chr=5&vc_start=156209107&vc_end=156212465) | [156212465](http://www.ensembl.org/Rattus_norvegicus/contigview?chr=5&vc_start=156209107&vc_end=156212465) | [NP_001103037.1](http://www.ensembl.org/Rattus_norvegicus/geneview?gene=NP_001103037.1) | hypothetical protein LOC690206 |
|  | [ENSRNOG00000021437](http://www.ensembl.org/Rattus_norvegicus/Gene/Summary?db=core;g=ENSRNOG00000021437) | [5](http://www.ensembl.org/Rattus_norvegicus/mapview?chr=5) | [156259586](http://www.ensembl.org/Rattus_norvegicus/contigview?chr=5&vc_start=156259586&vc_end=156324646) | [156324646](http://www.ensembl.org/Rattus_norvegicus/contigview?chr=5&vc_start=156259586&vc_end=156324646) | [IPI00388323.5](http://www.ensembl.org/Rattus_norvegicus/geneview?gene=IPI00388323.5) |  |
|  | [ENSRNOG00000042707](http://www.ensembl.org/Rattus_norvegicus/Gene/Summary?db=core;g=ENSRNOG00000042707) | [5](http://www.ensembl.org/Rattus_norvegicus/mapview?chr=5) | [156326218](http://www.ensembl.org/Rattus_norvegicus/contigview?chr=5&vc_start=156326218&vc_end=156326676) | [156326676](http://www.ensembl.org/Rattus_norvegicus/contigview?chr=5&vc_start=156326218&vc_end=156326676) | [O08591_RAT](http://www.ensembl.org/Rattus_norvegicus/geneview?gene=O08591_RAT) | Perlecan Fragment |
|  | [ENSRNOG00000013602](http://www.ensembl.org/Rattus_norvegicus/Gene/Summary?db=core;g=ENSRNOG00000013602) | [5](http://www.ensembl.org/Rattus_norvegicus/mapview?chr=5) | [156351117](http://www.ensembl.org/Rattus_norvegicus/contigview?chr=5&vc_start=156351117&vc_end=156417899) | [156417899](http://www.ensembl.org/Rattus_norvegicus/contigview?chr=5&vc_start=156351117&vc_end=156417899) | [UBP48_RAT](http://www.ensembl.org/Rattus_norvegicus/geneview?gene=UBP48_RAT) | Ubiquitin carboxyl-terminal hydrolase 48 |
|  | [ENSRNOG00000013825](http://www.ensembl.org/Rattus_norvegicus/Gene/Summary?db=core;g=ENSRNOG00000013825) | [5](http://www.ensembl.org/Rattus_norvegicus/mapview?chr=5) | [156424673](http://www.ensembl.org/Rattus_norvegicus/contigview?chr=5&vc_start=156424673&vc_end=156489590) | [156489590](http://www.ensembl.org/Rattus_norvegicus/contigview?chr=5&vc_start=156424673&vc_end=156489590) | [Q5EB70_RAT](http://www.ensembl.org/Rattus_norvegicus/geneview?gene=Q5EB70_RAT) | Rap1gap protein Fragment |
|  | [ENSRNOG00000014211](http://www.ensembl.org/Rattus_norvegicus/Gene/Summary?db=core;g=ENSRNOG00000014211) | [5](http://www.ensembl.org/Rattus_norvegicus/mapview?chr=5) | [156586343](http://www.ensembl.org/Rattus_norvegicus/contigview?chr=5&vc_start=156586343&vc_end=156586583) | [156586583](http://www.ensembl.org/Rattus_norvegicus/contigview?chr=5&vc_start=156586343&vc_end=156586583) |  |  |
|  | [ENSRNOG00000014241](http://www.ensembl.org/Rattus_norvegicus/Gene/Summary?db=core;g=ENSRNOG00000014241) | [5](http://www.ensembl.org/Rattus_norvegicus/mapview?chr=5) | [156635621](http://www.ensembl.org/Rattus_norvegicus/contigview?chr=5&vc_start=156635621&vc_end=156737433) | [156737433](http://www.ensembl.org/Rattus_norvegicus/contigview?chr=5&vc_start=156635621&vc_end=156737433) | [ECE1_RAT](http://www.ensembl.org/Rattus_norvegicus/geneview?gene=ECE1_RAT) | Endothelin-converting enzyme 1 |
|  | [ENSRNOG00000014368](http://www.ensembl.org/Rattus_norvegicus/Gene/Summary?db=core;g=ENSRNOG00000014368) | [5](http://www.ensembl.org/Rattus_norvegicus/mapview?chr=5) | [156809618](http://www.ensembl.org/Rattus_norvegicus/contigview?chr=5&vc_start=156809618&vc_end=156978841) | [156978841](http://www.ensembl.org/Rattus_norvegicus/contigview?chr=5&vc_start=156809618&vc_end=156978841) | [NP_001100163.1](http://www.ensembl.org/Rattus_norvegicus/geneview?gene=NP_001100163.1) | eukaryotic translation initiation factor 4 gamma, 3 |
|  | [ENSRNOG00000014445](http://www.ensembl.org/Rattus_norvegicus/Gene/Summary?db=core;g=ENSRNOG00000014445) | [5](http://www.ensembl.org/Rattus_norvegicus/mapview?chr=5) | [156996752](http://www.ensembl.org/Rattus_norvegicus/contigview?chr=5&vc_start=156996752&vc_end=157024041) | [157024041](http://www.ensembl.org/Rattus_norvegicus/contigview?chr=5&vc_start=156996752&vc_end=157024041) | [HP1B3_RAT](http://www.ensembl.org/Rattus_norvegicus/geneview?gene=HP1B3_RAT) | Heterochromatin protein 1-binding protein 3 |
|  | [ENSRNOG00000014909](http://www.ensembl.org/Rattus_norvegicus/Gene/Summary?db=core;g=ENSRNOG00000014909) | [5](http://www.ensembl.org/Rattus_norvegicus/mapview?chr=5) | [157028727](http://www.ensembl.org/Rattus_norvegicus/contigview?chr=5&vc_start=157028727&vc_end=157039797) | [157039797](http://www.ensembl.org/Rattus_norvegicus/contigview?chr=5&vc_start=157028727&vc_end=157039797) | [Sh2d5](http://www.ensembl.org/Rattus_norvegicus/geneview?gene=Sh2d5) |  |
|  | [ENSRNOG00000014970](http://www.ensembl.org/Rattus_norvegicus/Gene/Summary?db=core;g=ENSRNOG00000014970) | [5](http://www.ensembl.org/Rattus_norvegicus/mapview?chr=5) | [157043603](http://www.ensembl.org/Rattus_norvegicus/contigview?chr=5&vc_start=157043603&vc_end=157080111) | [157080111](http://www.ensembl.org/Rattus_norvegicus/contigview?chr=5&vc_start=157043603&vc_end=157080111) | [IPI00781261.1](http://www.ensembl.org/Rattus_norvegicus/geneview?gene=IPI00781261.1) |  |
|  | [ENSRNOG00000015079](http://www.ensembl.org/Rattus_norvegicus/Gene/Summary?db=core;g=ENSRNOG00000015079) | [5](http://www.ensembl.org/Rattus_norvegicus/mapview?chr=5) | [157083337](http://www.ensembl.org/Rattus_norvegicus/contigview?chr=5&vc_start=157083337&vc_end=157089835) | [157089835](http://www.ensembl.org/Rattus_norvegicus/contigview?chr=5&vc_start=157083337&vc_end=157089835) | [OST48_RAT](http://www.ensembl.org/Rattus_norvegicus/geneview?gene=OST48_RAT) | Dolichyl-diphosphooligosaccharide--protein glycosyltransferase |
|  | [ENSRNOG00000037091](http://www.ensembl.org/Rattus_norvegicus/Gene/Summary?db=core;g=ENSRNOG00000037091) | [5](http://www.ensembl.org/Rattus_norvegicus/mapview?chr=5) | [157172537](http://www.ensembl.org/Rattus_norvegicus/contigview?chr=5&vc_start=157172537&vc_end=157173321) | [157173321](http://www.ensembl.org/Rattus_norvegicus/contigview?chr=5&vc_start=157172537&vc_end=157173321) | [IPI00781615.1](http://www.ensembl.org/Rattus_norvegicus/geneview?gene=IPI00781615.1) |  |
|  | [ENSRNOG00000016010](http://www.ensembl.org/Rattus_norvegicus/Gene/Summary?db=core;g=ENSRNOG00000016010) | [5](http://www.ensembl.org/Rattus_norvegicus/mapview?chr=5) | [157213350](http://www.ensembl.org/Rattus_norvegicus/contigview?chr=5&vc_start=157213350&vc_end=157222401) | [157222401](http://www.ensembl.org/Rattus_norvegicus/contigview?chr=5&vc_start=157213350&vc_end=157222401) | [NP_001100165.1](http://www.ensembl.org/Rattus_norvegicus/geneview?gene=NP_001100165.1) | mitochondrial ubiquitin ligase activator of NFKB 1 |
|  | [ENSRNOG00000016322](http://www.ensembl.org/Rattus_norvegicus/Gene/Summary?db=core;g=ENSRNOG00000016322) | [5](http://www.ensembl.org/Rattus_norvegicus/mapview?chr=5) | [157235534](http://www.ensembl.org/Rattus_norvegicus/contigview?chr=5&vc_start=157235534&vc_end=157237314) | [157237314](http://www.ensembl.org/Rattus_norvegicus/contigview?chr=5&vc_start=157235534&vc_end=157237314) | [CK2N1_RAT](http://www.ensembl.org/Rattus_norvegicus/geneview?gene=CK2N1_RAT) | Calcium/calmodulin-dependent protein kinase II inhibitor 1 |
|  | [ENSRNOG00000027882](http://www.ensembl.org/Rattus_norvegicus/Gene/Summary?db=core;g=ENSRNOG00000027882) | [5](http://www.ensembl.org/Rattus_norvegicus/mapview?chr=5) | [157256432](http://www.ensembl.org/Rattus_norvegicus/contigview?chr=5&vc_start=157256432&vc_end=157256993) | [157256993](http://www.ensembl.org/Rattus_norvegicus/contigview?chr=5&vc_start=157256432&vc_end=157256993) | [XR_006862.1](http://www.ensembl.org/Rattus_norvegicus/geneview?gene=XR_006862.1) |  |
|  | [ENSRNOG00000016647](http://www.ensembl.org/Rattus_norvegicus/Gene/Summary?db=core;g=ENSRNOG00000016647) | [5](http://www.ensembl.org/Rattus_norvegicus/mapview?chr=5) | [157536546](http://www.ensembl.org/Rattus_norvegicus/contigview?chr=5&vc_start=157536546&vc_end=157555895) | [157555895](http://www.ensembl.org/Rattus_norvegicus/contigview?chr=5&vc_start=157536546&vc_end=157555895) | [PA2GC_RAT](http://www.ensembl.org/Rattus_norvegicus/geneview?gene=PA2GC_RAT) | Group IIC secretory phospholipase A2 Precursor |
|  | [ENSRNOG00000016826](http://www.ensembl.org/Rattus_norvegicus/Gene/Summary?db=core;g=ENSRNOG00000016826) | [5](http://www.ensembl.org/Rattus_norvegicus/mapview?chr=5) | [157594693](http://www.ensembl.org/Rattus_norvegicus/contigview?chr=5&vc_start=157594693&vc_end=157600861) | [157600861](http://www.ensembl.org/Rattus_norvegicus/contigview?chr=5&vc_start=157594693&vc_end=157600861) | [NP_001013446.1](http://www.ensembl.org/Rattus_norvegicus/geneview?gene=NP_001013446.1) | **phospholipase A2, group IID** |
|  | [ENSRNOG00000016945](http://www.ensembl.org/Rattus_norvegicus/Gene/Summary?db=core;g=ENSRNOG00000016945) | [5](http://www.ensembl.org/Rattus_norvegicus/mapview?chr=5) | [157654786](http://www.ensembl.org/Rattus_norvegicus/contigview?chr=5&vc_start=157654786&vc_end=157657360) | [157657360](http://www.ensembl.org/Rattus_norvegicus/contigview?chr=5&vc_start=157654786&vc_end=157657360) | [PA2GA_RAT](http://www.ensembl.org/Rattus_norvegicus/geneview?gene=PA2GA_RAT) | **Phospholipase A2, membrane associated Precursor** |
|  | [ENSRNOG00000017024](http://www.ensembl.org/Rattus_norvegicus/Gene/Summary?db=core;g=ENSRNOG00000017024) | [5](http://www.ensembl.org/Rattus_norvegicus/mapview?chr=5) | [157699794](http://www.ensembl.org/Rattus_norvegicus/contigview?chr=5&vc_start=157699794&vc_end=157705176) | [157705176](http://www.ensembl.org/Rattus_norvegicus/contigview?chr=5&vc_start=157699794&vc_end=157705176) | [NP_001100166.1](http://www.ensembl.org/Rattus_norvegicus/geneview?gene=NP_001100166.1) | **phospholipase A2, group IIE** |
|  | [ENSRNOG00000017401](http://www.ensembl.org/Rattus_norvegicus/Gene/Summary?db=core;g=ENSRNOG00000017401) | [5](http://www.ensembl.org/Rattus_norvegicus/mapview?chr=5) | [157796782](http://www.ensembl.org/Rattus_norvegicus/contigview?chr=5&vc_start=157796782&vc_end=157851042) | [157851042](http://www.ensembl.org/Rattus_norvegicus/contigview?chr=5&vc_start=157796782&vc_end=157851042) | [TMCO4_RAT](http://www.ensembl.org/Rattus_norvegicus/geneview?gene=TMCO4_RAT) | Transmembrane and coiled-coil domain-containing protein 4 |
|  | [ENSRNOG00000007330](http://www.ensembl.org/Rattus_norvegicus/Gene/Summary?db=core;g=ENSRNOG00000007330) | [5](http://www.ensembl.org/Rattus_norvegicus/mapview?chr=5) | [157980877](http://www.ensembl.org/Rattus_norvegicus/contigview?chr=5&vc_start=157980877&vc_end=158080678) | [158080678](http://www.ensembl.org/Rattus_norvegicus/contigview?chr=5&vc_start=157980877&vc_end=158080678) | [CAPZB_RAT](http://www.ensembl.org/Rattus_norvegicus/geneview?gene=CAPZB_RAT) | F-actin-capping protein subunit beta (CapZ beta) |
|  | [ENSRNOG00000017780](http://www.ensembl.org/Rattus_norvegicus/Gene/Summary?db=core;g=ENSRNOG00000017780) | [5](http://www.ensembl.org/Rattus_norvegicus/mapview?chr=5) | [158097641](http://www.ensembl.org/Rattus_norvegicus/contigview?chr=5&vc_start=158097641&vc_end=158106199) | [158106199](http://www.ensembl.org/Rattus_norvegicus/contigview?chr=5&vc_start=158097641&vc_end=158106199) | [ARK72_RAT](http://www.ensembl.org/Rattus_norvegicus/geneview?gene=ARK72_RAT) | Aflatoxin B1 aldehyde reductase member 2 |
|  | [ENSRNOG00000017899](http://www.ensembl.org/Rattus_norvegicus/Gene/Summary?db=core;g=ENSRNOG00000017899) | [5](http://www.ensembl.org/Rattus_norvegicus/mapview?chr=5) | [158139986](http://www.ensembl.org/Rattus_norvegicus/contigview?chr=5&vc_start=158139986&vc_end=158147516) | [158147516](http://www.ensembl.org/Rattus_norvegicus/contigview?chr=5&vc_start=158139986&vc_end=158147516) | [ARK73_RAT](http://www.ensembl.org/Rattus_norvegicus/geneview?gene=ARK73_RAT) | Aflatoxin B1 aldehyde reductase member 3 |
|  | [ENSRNOG00000018097](http://www.ensembl.org/Rattus_norvegicus/Gene/Summary?db=core;g=ENSRNOG00000018097) | [5](http://www.ensembl.org/Rattus_norvegicus/mapview?chr=5) | [158154690](http://www.ensembl.org/Rattus_norvegicus/contigview?chr=5&vc_start=158154690&vc_end=158180008) | [158180008](http://www.ensembl.org/Rattus_norvegicus/contigview?chr=5&vc_start=158154690&vc_end=158180008) | [NP_001102160.1](http://www.ensembl.org/Rattus_norvegicus/geneview?gene=NP_001102160.1) | hypothetical protein LOC362643 |
|  | [ENSRNOG00000018183](http://www.ensembl.org/Rattus_norvegicus/Gene/Summary?db=core;g=ENSRNOG00000018183) | [5](http://www.ensembl.org/Rattus_norvegicus/mapview?chr=5) | [158181988](http://www.ensembl.org/Rattus_norvegicus/contigview?chr=5&vc_start=158181988&vc_end=158289896) | [158289896](http://www.ensembl.org/Rattus_norvegicus/contigview?chr=5&vc_start=158181988&vc_end=158289896) | [UBR4_RAT](http://www.ensembl.org/Rattus_norvegicus/geneview?gene=UBR4_RAT) | E3 ubiquitin-protein ligase UBR4 |
|  | [ENSRNOG00000042920](http://www.ensembl.org/Rattus_norvegicus/Gene/Summary?db=core;g=ENSRNOG00000042920) | [5](http://www.ensembl.org/Rattus_norvegicus/mapview?chr=5) | [158287475](http://www.ensembl.org/Rattus_norvegicus/contigview?chr=5&vc_start=158287475&vc_end=158452408) | [158452408](http://www.ensembl.org/Rattus_norvegicus/contigview?chr=5&vc_start=158287475&vc_end=158452408) | [AL4A1_RAT](http://www.ensembl.org/Rattus_norvegicus/geneview?gene=AL4A1_RAT) | Delta-1-pyrroline-5-carboxylate dehydrogenase |
|  | [ENSRNOG00000026383](http://www.ensembl.org/Rattus_norvegicus/Gene/Summary?db=core;g=ENSRNOG00000026383) | [5](http://www.ensembl.org/Rattus_norvegicus/mapview?chr=5) | [158339222](http://www.ensembl.org/Rattus_norvegicus/contigview?chr=5&vc_start=158339222&vc_end=158339502) | [158339502](http://www.ensembl.org/Rattus_norvegicus/contigview?chr=5&vc_start=158339222&vc_end=158339502) |  |  |
|  | [ENSRNOG00000042417](http://www.ensembl.org/Rattus_norvegicus/Gene/Summary?db=core;g=ENSRNOG00000042417) | [5](http://www.ensembl.org/Rattus_norvegicus/mapview?chr=5) | [158404338](http://www.ensembl.org/Rattus_norvegicus/contigview?chr=5&vc_start=158404338&vc_end=158418394) | [158418394](http://www.ensembl.org/Rattus_norvegicus/contigview?chr=5&vc_start=158404338&vc_end=158418394) | [IFFO2_RAT](http://www.ensembl.org/Rattus_norvegicus/geneview?gene=IFFO2_RAT) | Intermediate filament family orphan 2 |
|  | [ENSRNOG00000018608](http://www.ensembl.org/Rattus_norvegicus/Gene/Summary?db=core;g=ENSRNOG00000018608) | [5](http://www.ensembl.org/Rattus_norvegicus/mapview?chr=5) | [158457995](http://www.ensembl.org/Rattus_norvegicus/contigview?chr=5&vc_start=158457995&vc_end=158474013) | [158474013](http://www.ensembl.org/Rattus_norvegicus/contigview?chr=5&vc_start=158457995&vc_end=158474013) | [TS1R2_RAT](http://www.ensembl.org/Rattus_norvegicus/geneview?gene=TS1R2_RAT) | Taste receptor type 1 member 2 Precursor |
|  | [ENSRNOG00000006327](http://www.ensembl.org/Rattus_norvegicus/Gene/Summary?db=core;g=ENSRNOG00000006327) | [5](http://www.ensembl.org/Rattus_norvegicus/mapview?chr=5) | [159540931](http://www.ensembl.org/Rattus_norvegicus/contigview?chr=5&vc_start=159540931&vc_end=159554484) | [159554484](http://www.ensembl.org/Rattus_norvegicus/contigview?chr=5&vc_start=159540931&vc_end=159554484) | [Rcc2](http://www.ensembl.org/Rattus_norvegicus/geneview?gene=Rcc2) |  |
|  | [ENSRNOG00000007574](http://www.ensembl.org/Rattus_norvegicus/Gene/Summary?db=core;g=ENSRNOG00000007574) | [5](http://www.ensembl.org/Rattus_norvegicus/mapview?chr=5) | [159762824](http://www.ensembl.org/Rattus_norvegicus/contigview?chr=5&vc_start=159762824&vc_end=159804039) | [159804039](http://www.ensembl.org/Rattus_norvegicus/contigview?chr=5&vc_start=159762824&vc_end=159804039) | [PADI2_RAT](http://www.ensembl.org/Rattus_norvegicus/geneview?gene=PADI2_RAT) | Protein-arginine deiminase type-2 |
|  | [ENSRNOG00000007967](http://www.ensembl.org/Rattus_norvegicus/Gene/Summary?db=core;g=ENSRNOG00000007967) | [5](http://www.ensembl.org/Rattus_norvegicus/mapview?chr=5) | [159818669](http://www.ensembl.org/Rattus_norvegicus/contigview?chr=5&vc_start=159818669&vc_end=159839767) | [159839767](http://www.ensembl.org/Rattus_norvegicus/contigview?chr=5&vc_start=159818669&vc_end=159839767) | [DHSB_RAT](http://www.ensembl.org/Rattus_norvegicus/geneview?gene=DHSB_RAT) | Succinate dehydrogenase [ubiquinone] |
|  | [ENSRNOG00000008052](http://www.ensembl.org/Rattus_norvegicus/Gene/Summary?db=core;g=ENSRNOG00000008052) | [5](http://www.ensembl.org/Rattus_norvegicus/mapview?chr=5) | [159847016](http://www.ensembl.org/Rattus_norvegicus/contigview?chr=5&vc_start=159847016&vc_end=159866361) | [159866361](http://www.ensembl.org/Rattus_norvegicus/contigview?chr=5&vc_start=159847016&vc_end=159866361) | [B5DEH6_RAT](http://www.ensembl.org/Rattus_norvegicus/geneview?gene=B5DEH6_RAT) | Atp13a2 protein |
|  | [ENSRNOG00000008257](http://www.ensembl.org/Rattus_norvegicus/Gene/Summary?db=core;g=ENSRNOG00000008257) | [5](http://www.ensembl.org/Rattus_norvegicus/mapview?chr=5) | [159868933](http://www.ensembl.org/Rattus_norvegicus/contigview?chr=5&vc_start=159868933&vc_end=159874481) | [159874481](http://www.ensembl.org/Rattus_norvegicus/contigview?chr=5&vc_start=159868933&vc_end=159874481) | [NP_001101459.1](http://www.ensembl.org/Rattus_norvegicus/geneview?gene=NP_001101459.1) | microfibrillar-associated protein 2 |
|  | [ENSRNOG00000009058](http://www.ensembl.org/Rattus_norvegicus/Gene/Summary?db=core;g=ENSRNOG00000009058) | [5](http://www.ensembl.org/Rattus_norvegicus/mapview?chr=5) | [159947710](http://www.ensembl.org/Rattus_norvegicus/contigview?chr=5&vc_start=159947710&vc_end=159972904) | [159972904](http://www.ensembl.org/Rattus_norvegicus/contigview?chr=5&vc_start=159947710&vc_end=159972904) | [SPT21_RAT](http://www.ensembl.org/Rattus_norvegicus/geneview?gene=SPT21_RAT) | Spermatogenesis-associated protein 21 |
|  | [ENSRNOG00000014305](http://www.ensembl.org/Rattus_norvegicus/Gene/Summary?db=core;g=ENSRNOG00000014305) | [5](http://www.ensembl.org/Rattus_norvegicus/mapview?chr=5) | [160005496](http://www.ensembl.org/Rattus_norvegicus/contigview?chr=5&vc_start=160005496&vc_end=160061687) | [160061687](http://www.ensembl.org/Rattus_norvegicus/contigview?chr=5&vc_start=160005496&vc_end=160061687) | [IPI00359126.2](http://www.ensembl.org/Rattus_norvegicus/geneview?gene=IPI00359126.2) |  |
|  | [ENSRNOG00000023346](http://www.ensembl.org/Rattus_norvegicus/Gene/Summary?db=core;g=ENSRNOG00000023346) | [5](http://www.ensembl.org/Rattus_norvegicus/mapview?chr=5) | [160070852](http://www.ensembl.org/Rattus_norvegicus/contigview?chr=5&vc_start=160070852&vc_end=160076478) | [160076478](http://www.ensembl.org/Rattus_norvegicus/contigview?chr=5&vc_start=160070852&vc_end=160076478) | [NP_001102742.1](http://www.ensembl.org/Rattus_norvegicus/geneview?gene=NP_001102742.1) | hypothetical protein LOC500576 |
|  | [ENSRNOG00000023313](http://www.ensembl.org/Rattus_norvegicus/Gene/Summary?db=core;g=ENSRNOG00000023313) | [5](http://www.ensembl.org/Rattus_norvegicus/mapview?chr=5) | [160091794](http://www.ensembl.org/Rattus_norvegicus/contigview?chr=5&vc_start=160091794&vc_end=160109955) | [160109955](http://www.ensembl.org/Rattus_norvegicus/contigview?chr=5&vc_start=160091794&vc_end=160109955) | [NP_001102162.1](http://www.ensembl.org/Rattus_norvegicus/geneview?gene=NP_001102162.1) | Rho guanine nucleotide exchange factor (GEF) 19 |
|  | [ENSRNOG00000009222](http://www.ensembl.org/Rattus_norvegicus/Gene/Summary?db=core;g=ENSRNOG00000009222) | [5](http://www.ensembl.org/Rattus_norvegicus/mapview?chr=5) | [160185253](http://www.ensembl.org/Rattus_norvegicus/contigview?chr=5&vc_start=160185253&vc_end=160214727) | [160214727](http://www.ensembl.org/Rattus_norvegicus/contigview?chr=5&vc_start=160185253&vc_end=160214727) | [NP_001102447.1](http://www.ensembl.org/Rattus_norvegicus/geneview?gene=NP_001102447.1) | **ephrin receptor EphA2** |
|  | [ENSRNOG00000037062](http://www.ensembl.org/Rattus_norvegicus/Gene/Summary?db=core;g=ENSRNOG00000037062) | [5](http://www.ensembl.org/Rattus_norvegicus/mapview?chr=5) | [160237393](http://www.ensembl.org/Rattus_norvegicus/contigview?chr=5&vc_start=160237393&vc_end=160464928) | [160464928](http://www.ensembl.org/Rattus_norvegicus/contigview?chr=5&vc_start=160237393&vc_end=160464928) | [IPI00568873.2](http://www.ensembl.org/Rattus_norvegicus/geneview?gene=IPI00568873.2) |  |
|  | [ENSRNOG00000023008](http://www.ensembl.org/Rattus_norvegicus/Gene/Summary?db=core;g=ENSRNOG00000023008) | [5](http://www.ensembl.org/Rattus_norvegicus/mapview?chr=5) | [160269118](http://www.ensembl.org/Rattus_norvegicus/contigview?chr=5&vc_start=160269118&vc_end=160273424) | [160273424](http://www.ensembl.org/Rattus_norvegicus/contigview?chr=5&vc_start=160269118&vc_end=160273424) | [Fam131c](http://www.ensembl.org/Rattus_norvegicus/geneview?gene=Fam131c) |  |
|  | [ENSRNOG00000029079](http://www.ensembl.org/Rattus_norvegicus/Gene/Summary?db=core;g=ENSRNOG00000029079) | [5](http://www.ensembl.org/Rattus_norvegicus/mapview?chr=5) | [160311908](http://www.ensembl.org/Rattus_norvegicus/contigview?chr=5&vc_start=160311908&vc_end=160315392) | [160315392](http://www.ensembl.org/Rattus_norvegicus/contigview?chr=5&vc_start=160311908&vc_end=160315392) | [HSPB7_RAT](http://www.ensembl.org/Rattus_norvegicus/geneview?gene=HSPB7_RAT) | Heat shock protein beta-7 Fragment (HspB7) |
|  | [ENSRNOG00000010436](http://www.ensembl.org/Rattus_norvegicus/Gene/Summary?db=core;g=ENSRNOG00000010436) | [5](http://www.ensembl.org/Rattus_norvegicus/mapview?chr=5) | [160337988](http://www.ensembl.org/Rattus_norvegicus/contigview?chr=5&vc_start=160337988&vc_end=160358953) | [160358953](http://www.ensembl.org/Rattus_norvegicus/contigview?chr=5&vc_start=160337988&vc_end=160358953) | [NP_001012105.1](http://www.ensembl.org/Rattus_norvegicus/geneview?gene=NP_001012105.1) | zinc finger and BTB domain containing 17 |
|  | [ENSRNOG00000037053](http://www.ensembl.org/Rattus_norvegicus/Gene/Summary?db=core;g=ENSRNOG00000037053) | [5](http://www.ensembl.org/Rattus_norvegicus/mapview?chr=5) | [160514756](http://www.ensembl.org/Rattus_norvegicus/contigview?chr=5&vc_start=160514756&vc_end=160515242) | [160515242](http://www.ensembl.org/Rattus_norvegicus/contigview?chr=5&vc_start=160514756&vc_end=160515242) | [IPI00779426.1](http://www.ensembl.org/Rattus_norvegicus/geneview?gene=IPI00779426.1) |  |
|  | [ENSRNOG00000012315](http://www.ensembl.org/Rattus_norvegicus/Gene/Summary?db=core;g=ENSRNOG00000012315) | [5](http://www.ensembl.org/Rattus_norvegicus/mapview?chr=5) | [160646314](http://www.ensembl.org/Rattus_norvegicus/contigview?chr=5&vc_start=160646314&vc_end=160660437) | [160660437](http://www.ensembl.org/Rattus_norvegicus/contigview?chr=5&vc_start=160646314&vc_end=160660437) | [SPEB_RAT](http://www.ensembl.org/Rattus_norvegicus/geneview?gene=SPEB_RAT) | Agmatinase, mitochondrial Precursor ( |
|  | [ENSRNOG00000012944](http://www.ensembl.org/Rattus_norvegicus/Gene/Summary?db=core;g=ENSRNOG00000012944) | [5](http://www.ensembl.org/Rattus_norvegicus/mapview?chr=5) | [160704225](http://www.ensembl.org/Rattus_norvegicus/contigview?chr=5&vc_start=160704225&vc_end=160721800) | [160721800](http://www.ensembl.org/Rattus_norvegicus/contigview?chr=5&vc_start=160704225&vc_end=160721800) | [NP_113820.1](http://www.ensembl.org/Rattus_norvegicus/geneview?gene=NP_113820.1) | **caspase 9, apoptosis-related cysteine peptidase** |
|  | [ENSRNOG00000033153](http://www.ensembl.org/Rattus_norvegicus/Gene/Summary?db=core;g=ENSRNOG00000033153) | [5](http://www.ensembl.org/Rattus_norvegicus/mapview?chr=5) | [151479150](http://www.ensembl.org/Rattus_norvegicus/contigview?chr=5&vc_start=151479150&vc_end=151480388) | [151480388](http://www.ensembl.org/Rattus_norvegicus/contigview?chr=5&vc_start=151479150&vc_end=151480388) | [NP_001013963.1](http://www.ensembl.org/Rattus_norvegicus/geneview?gene=NP_001013963.1) | hypothetical protein LOC298795 |
|  | [ENSRNOG00000009540](http://www.ensembl.org/Rattus_norvegicus/Gene/Summary?db=core;g=ENSRNOG00000009540) | [5](http://www.ensembl.org/Rattus_norvegicus/mapview?chr=5) | [151915699](http://www.ensembl.org/Rattus_norvegicus/contigview?chr=5&vc_start=151915699&vc_end=151916688) | [151916688](http://www.ensembl.org/Rattus_norvegicus/contigview?chr=5&vc_start=151915699&vc_end=151916688) | [NP_714949.1](http://www.ensembl.org/Rattus_norvegicus/geneview?gene=NP_714949.1) | G protein-coupled receptor 3 |
|  | [ENSRNOG00000029754](http://www.ensembl.org/Rattus_norvegicus/Gene/Summary?db=core;g=ENSRNOG00000029754) | [5](http://www.ensembl.org/Rattus_norvegicus/mapview?chr=5) | [152707601](http://www.ensembl.org/Rattus_norvegicus/contigview?chr=5&vc_start=152707601&vc_end=152707846) | [152707846](http://www.ensembl.org/Rattus_norvegicus/contigview?chr=5&vc_start=152707601&vc_end=152707846) | [LOC689346](http://www.ensembl.org/Rattus_norvegicus/geneview?gene=LOC689346) |  |
|  | [ENSRNOG00000022054](http://www.ensembl.org/Rattus_norvegicus/Gene/Summary?db=core;g=ENSRNOG00000022054) | [5](http://www.ensembl.org/Rattus_norvegicus/mapview?chr=5) | [153263432](http://www.ensembl.org/Rattus_norvegicus/contigview?chr=5&vc_start=153263432&vc_end=153264469) | [153264469](http://www.ensembl.org/Rattus_norvegicus/contigview?chr=5&vc_start=153263432&vc_end=153264469) | [NP_001029253.1](http://www.ensembl.org/Rattus_norvegicus/geneview?gene=NP_001029253.1) | progestin and adipoQ receptor family member VII |
|  | [ENSRNOG00000037174](http://www.ensembl.org/Rattus_norvegicus/Gene/Summary?db=core;g=ENSRNOG00000037174) | [5](http://www.ensembl.org/Rattus_norvegicus/mapview?chr=5) | [153917122](http://www.ensembl.org/Rattus_norvegicus/contigview?chr=5&vc_start=153917122&vc_end=153917178) | [153917178](http://www.ensembl.org/Rattus_norvegicus/contigview?chr=5&vc_start=153917122&vc_end=153917178) | [IPI00782458.1](http://www.ensembl.org/Rattus_norvegicus/geneview?gene=IPI00782458.1) |  |
|  | [ENSRNOG00000012038](http://www.ensembl.org/Rattus_norvegicus/Gene/Summary?db=core;g=ENSRNOG00000012038) | [5](http://www.ensembl.org/Rattus_norvegicus/mapview?chr=5) | [155188669](http://www.ensembl.org/Rattus_norvegicus/contigview?chr=5&vc_start=155188669&vc_end=155189793) | [155189793](http://www.ensembl.org/Rattus_norvegicus/contigview?chr=5&vc_start=155188669&vc_end=155189793) | [5HT1D_RAT](http://www.ensembl.org/Rattus_norvegicus/geneview?gene=5HT1D_RAT) | **5-hydroxytryptamine receptor 1D** |
|  | [ENSRNOG00000029657](http://www.ensembl.org/Rattus_norvegicus/Gene/Summary?db=core;g=ENSRNOG00000029657) | [5](http://www.ensembl.org/Rattus_norvegicus/mapview?chr=5) | [155348459](http://www.ensembl.org/Rattus_norvegicus/contigview?chr=5&vc_start=155348459&vc_end=155348893) | [155348893](http://www.ensembl.org/Rattus_norvegicus/contigview?chr=5&vc_start=155348459&vc_end=155348893) |  |  |
|  | [ENSRNOG00000037119](http://www.ensembl.org/Rattus_norvegicus/Gene/Summary?db=core;g=ENSRNOG00000037119) | [5](http://www.ensembl.org/Rattus_norvegicus/mapview?chr=5) | [156083962](http://www.ensembl.org/Rattus_norvegicus/contigview?chr=5&vc_start=156083962&vc_end=156084201) | [156084201](http://www.ensembl.org/Rattus_norvegicus/contigview?chr=5&vc_start=156083962&vc_end=156084201) | [IPI00779169.1](http://www.ensembl.org/Rattus_norvegicus/geneview?gene=IPI00779169.1) |  |
|  | [ENSRNOG00000017244](http://www.ensembl.org/Rattus_norvegicus/Gene/Summary?db=core;g=ENSRNOG00000017244) | [5](http://www.ensembl.org/Rattus_norvegicus/mapview?chr=5) | [157790446](http://www.ensembl.org/Rattus_norvegicus/contigview?chr=5&vc_start=157790446&vc_end=157791670) | [157791670](http://www.ensembl.org/Rattus_norvegicus/contigview?chr=5&vc_start=157790446&vc_end=157791670) | [NP_001103062.1](http://www.ensembl.org/Rattus_norvegicus/geneview?gene=NP_001103062.1) | ring finger protein 186 |
|  | [ENSRNOG00000033916](http://www.ensembl.org/Rattus_norvegicus/Gene/Summary?db=core;g=ENSRNOG00000033916) | [5](http://www.ensembl.org/Rattus_norvegicus/mapview?chr=5) | [159941519](http://www.ensembl.org/Rattus_norvegicus/contigview?chr=5&vc_start=159941519&vc_end=159941829) | [159941829](http://www.ensembl.org/Rattus_norvegicus/contigview?chr=5&vc_start=159941519&vc_end=159941829) | [IPI00480790.1](http://www.ensembl.org/Rattus_norvegicus/geneview?gene=IPI00480790.1) |  |
|  | [ENSRNOG00000033541](http://www.ensembl.org/Rattus_norvegicus/Gene/Summary?db=core;g=ENSRNOG00000033541) | [5](http://www.ensembl.org/Rattus_norvegicus/mapview?chr=5) | [151295235](http://www.ensembl.org/Rattus_norvegicus/contigview?chr=5&vc_start=151295235&vc_end=151295631) | [151295631](http://www.ensembl.org/Rattus_norvegicus/contigview?chr=5&vc_start=151295235&vc_end=151295631) |  |  |
|  | [ENSRNOG00000031506](http://www.ensembl.org/Rattus_norvegicus/Gene/Summary?db=core;g=ENSRNOG00000031506) | [5](http://www.ensembl.org/Rattus_norvegicus/mapview?chr=5) | [152846138](http://www.ensembl.org/Rattus_norvegicus/contigview?chr=5&vc_start=152846138&vc_end=152846862) | [152846862](http://www.ensembl.org/Rattus_norvegicus/contigview?chr=5&vc_start=152846138&vc_end=152846862) | [XR_006958.1](http://www.ensembl.org/Rattus_norvegicus/geneview?gene=XR_006958.1) |  |
|  | [ENSRNOG00000043531](http://www.ensembl.org/Rattus_norvegicus/Gene/Summary?db=core;g=ENSRNOG00000043531) | [5](http://www.ensembl.org/Rattus_norvegicus/mapview?chr=5) | [153662507](http://www.ensembl.org/Rattus_norvegicus/contigview?chr=5&vc_start=153662507&vc_end=153662602) | [153662602](http://www.ensembl.org/Rattus_norvegicus/contigview?chr=5&vc_start=153662507&vc_end=153662602) |  |  |
|  | [ENSRNOG00000040741](http://www.ensembl.org/Rattus_norvegicus/Gene/Summary?db=core;g=ENSRNOG00000040741) | [5](http://www.ensembl.org/Rattus_norvegicus/mapview?chr=5) | [152310540](http://www.ensembl.org/Rattus_norvegicus/contigview?chr=5&vc_start=152310540&vc_end=152310703) | [152310703](http://www.ensembl.org/Rattus_norvegicus/contigview?chr=5&vc_start=152310540&vc_end=152310703) | [SCARNA1](http://www.ensembl.org/Rattus_norvegicus/geneview?gene=SCARNA1) | Small Cajal body specific RNA 1 |
|  | [ENSRNOG00000036469](http://www.ensembl.org/Rattus_norvegicus/Gene/Summary?db=core;g=ENSRNOG00000036469) | [5](http://www.ensembl.org/Rattus_norvegicus/mapview?chr=5) | [156608875](http://www.ensembl.org/Rattus_norvegicus/contigview?chr=5&vc_start=156608875&vc_end=156609015) | [156609015](http://www.ensembl.org/Rattus_norvegicus/contigview?chr=5&vc_start=156608875&vc_end=156609015) | [U4](http://www.ensembl.org/Rattus_norvegicus/geneview?gene=U4) | U4 spliceosomal RNA |
|  | [ENSRNOG00000043560](http://www.ensembl.org/Rattus_norvegicus/Gene/Summary?db=core;g=ENSRNOG00000043560) | [5](http://www.ensembl.org/Rattus_norvegicus/mapview?chr=5) | [152524359](http://www.ensembl.org/Rattus_norvegicus/contigview?chr=5&vc_start=152524359&vc_end=152524431) | [152524431](http://www.ensembl.org/Rattus_norvegicus/contigview?chr=5&vc_start=152524359&vc_end=152524431) |  |  |
|  | [ENSRNOG00000034426](http://www.ensembl.org/Rattus_norvegicus/Gene/Summary?db=core;g=ENSRNOG00000034426) | [5](http://www.ensembl.org/Rattus_norvegicus/mapview?chr=5) | [159944334](http://www.ensembl.org/Rattus_norvegicus/contigview?chr=5&vc_start=159944334&vc_end=159944440) | [159944440](http://www.ensembl.org/Rattus_norvegicus/contigview?chr=5&vc_start=159944334&vc_end=159944440) | [U6](http://www.ensembl.org/Rattus_norvegicus/geneview?gene=U6) | U6 spliceosomal RNA |
|  | [ENSRNOG00000036471](http://www.ensembl.org/Rattus_norvegicus/Gene/Summary?db=core;g=ENSRNOG00000036471) | [5](http://www.ensembl.org/Rattus_norvegicus/mapview?chr=5) | [152846611](http://www.ensembl.org/Rattus_norvegicus/contigview?chr=5&vc_start=152846611&vc_end=152846716) | [152846716](http://www.ensembl.org/Rattus_norvegicus/contigview?chr=5&vc_start=152846611&vc_end=152846716) |  |  |
|  | [ENSRNOG00000034502](http://www.ensembl.org/Rattus_norvegicus/Gene/Summary?db=core;g=ENSRNOG00000034502) | [5](http://www.ensembl.org/Rattus_norvegicus/mapview?chr=5) | [159552803](http://www.ensembl.org/Rattus_norvegicus/contigview?chr=5&vc_start=159552803&vc_end=159552932) | [159552932](http://www.ensembl.org/Rattus_norvegicus/contigview?chr=5&vc_start=159552803&vc_end=159552932) | [SNORA15](http://www.ensembl.org/Rattus_norvegicus/geneview?gene=SNORA15) | Small nucleolar RNA SNORA15 |
|  | [ENSRNOG00000040619](http://www.ensembl.org/Rattus_norvegicus/Gene/Summary?db=core;g=ENSRNOG00000040619) | [5](http://www.ensembl.org/Rattus_norvegicus/mapview?chr=5) | [155791349](http://www.ensembl.org/Rattus_norvegicus/contigview?chr=5&vc_start=155791349&vc_end=155791478) | [155791478](http://www.ensembl.org/Rattus_norvegicus/contigview?chr=5&vc_start=155791349&vc_end=155791478) | [SNORA17](http://www.ensembl.org/Rattus_norvegicus/geneview?gene=SNORA17) | Small nucleolar RNA SNORA17 |
|  | [ENSRNOG00000035382](http://www.ensembl.org/Rattus_norvegicus/Gene/Summary?db=core;g=ENSRNOG00000035382) | [5](http://www.ensembl.org/Rattus_norvegicus/mapview?chr=5) | [152398005](http://www.ensembl.org/Rattus_norvegicus/contigview?chr=5&vc_start=152398005&vc_end=152398111) | [152398111](http://www.ensembl.org/Rattus_norvegicus/contigview?chr=5&vc_start=152398005&vc_end=152398111) | [U6](http://www.ensembl.org/Rattus_norvegicus/geneview?gene=U6) | U6 spliceosomal RNA |
| **Parameters - SERT-/-** | **Ensembl Gene ID** | **Chr.** | **Gene Start** | **Gene End** | **Gene Name** | **Description** |
| Cocaine-induced | [ENSRNOG00000033711](http://www.ensembl.org/Rattus_norvegicus/Gene/Summary?db=core;g=ENSRNOG00000033711) | [8](http://www.ensembl.org/Rattus_norvegicus/mapview?chr=8) | [44321665](http://www.ensembl.org/Rattus_norvegicus/contigview?chr=8&vc_start=44321665&vc_end=44322881) | [44322881](http://www.ensembl.org/Rattus_norvegicus/contigview?chr=8&vc_start=44321665&vc_end=44322881) |  |  |
| time in centre | [ENSRNOG00000041330](http://www.ensembl.org/Rattus_norvegicus/Gene/Summary?db=core;g=ENSRNOG00000041330) | [8](http://www.ensembl.org/Rattus_norvegicus/mapview?chr=8) | [44515566](http://www.ensembl.org/Rattus_norvegicus/contigview?chr=8&vc_start=44515566&vc_end=44515656) | [44515656](http://www.ensembl.org/Rattus_norvegicus/contigview?chr=8&vc_start=44515566&vc_end=44515656) |  |  |
|  | [ENSRNOG00000035502](http://www.ensembl.org/Rattus_norvegicus/Gene/Summary?db=core;g=ENSRNOG00000035502) | [8](http://www.ensembl.org/Rattus_norvegicus/mapview?chr=8) | [44518670](http://www.ensembl.org/Rattus_norvegicus/contigview?chr=8&vc_start=44518670&vc_end=44518749) | [44518749](http://www.ensembl.org/Rattus_norvegicus/contigview?chr=8&vc_start=44518670&vc_end=44518749) | [rno-mir-100](http://www.ensembl.org/Rattus_norvegicus/geneview?gene=rno-mir-100) | rno-mir-100 |
|  | [ENSRNOG00000035548](http://www.ensembl.org/Rattus_norvegicus/Gene/Summary?db=core;g=ENSRNOG00000035548) | [8](http://www.ensembl.org/Rattus_norvegicus/mapview?chr=8) | [44525769](http://www.ensembl.org/Rattus_norvegicus/contigview?chr=8&vc_start=44525769&vc_end=44525864) | [44525864](http://www.ensembl.org/Rattus_norvegicus/contigview?chr=8&vc_start=44525769&vc_end=44525864) | [rno-let-7a-2](http://www.ensembl.org/Rattus_norvegicus/geneview?gene=rno-let-7a-2) | rno-let-7a-2 |
|  | [ENSRNOG00000039200](http://www.ensembl.org/Rattus_norvegicus/Gene/Summary?db=core;g=ENSRNOG00000039200) | [8](http://www.ensembl.org/Rattus_norvegicus/mapview?chr=8) | [44569210](http://www.ensembl.org/Rattus_norvegicus/contigview?chr=8&vc_start=44569210&vc_end=44578533) | [44578533](http://www.ensembl.org/Rattus_norvegicus/contigview?chr=8&vc_start=44569210&vc_end=44578533) | [IPI00776999.1](http://www.ensembl.org/Rattus_norvegicus/geneview?gene=IPI00776999.1) |  |
|  | [ENSRNOG00000035550](http://www.ensembl.org/Rattus_norvegicus/Gene/Summary?db=core;g=ENSRNOG00000035550) | [8](http://www.ensembl.org/Rattus_norvegicus/mapview?chr=8) | [44570155](http://www.ensembl.org/Rattus_norvegicus/contigview?chr=8&vc_start=44570155&vc_end=44570241) | [44570241](http://www.ensembl.org/Rattus_norvegicus/contigview?chr=8&vc_start=44570155&vc_end=44570241) | [rno-mir-125b-1](http://www.ensembl.org/Rattus_norvegicus/geneview?gene=rno-mir-125b-1) | rno-mir-125b-1 |
|  | [ENSRNOG00000021771](http://www.ensembl.org/Rattus_norvegicus/Gene/Summary?db=core;g=ENSRNOG00000021771) | [8](http://www.ensembl.org/Rattus_norvegicus/mapview?chr=8) | [46312645](http://www.ensembl.org/Rattus_norvegicus/contigview?chr=8&vc_start=46312645&vc_end=46337389) | [46337389](http://www.ensembl.org/Rattus_norvegicus/contigview?chr=8&vc_start=46312645&vc_end=46337389) | [NP_001100285.1](http://www.ensembl.org/Rattus_norvegicus/geneview?gene=NP_001100285.1) | tripartite motif-containing 29 |
|  | [ENSRNOG00000041456](http://www.ensembl.org/Rattus_norvegicus/Gene/Summary?db=core;g=ENSRNOG00000041456) | [8](http://www.ensembl.org/Rattus_norvegicus/mapview?chr=8) | [46391215](http://www.ensembl.org/Rattus_norvegicus/contigview?chr=8&vc_start=46391215&vc_end=46391330) | [46391330](http://www.ensembl.org/Rattus_norvegicus/contigview?chr=8&vc_start=46391215&vc_end=46391330) |  |  |
|  | [ENSRNOG00000006403](http://www.ensembl.org/Rattus_norvegicus/Gene/Summary?db=core;g=ENSRNOG00000006403) | [8](http://www.ensembl.org/Rattus_norvegicus/mapview?chr=8) | [46785431](http://www.ensembl.org/Rattus_norvegicus/contigview?chr=8&vc_start=46785431&vc_end=46798633) | [46798633](http://www.ensembl.org/Rattus_norvegicus/contigview?chr=8&vc_start=46785431&vc_end=46798633) | [Q9WTY6_RAT](http://www.ensembl.org/Rattus_norvegicus/geneview?gene=Q9WTY6_RAT) | Nectin-1 Fragment |
|  | [ENSRNOG00000039124](http://www.ensembl.org/Rattus_norvegicus/Gene/Summary?db=core;g=ENSRNOG00000039124) | [8](http://www.ensembl.org/Rattus_norvegicus/mapview?chr=8) | [46824565](http://www.ensembl.org/Rattus_norvegicus/contigview?chr=8&vc_start=46824565&vc_end=46827474) | [46827474](http://www.ensembl.org/Rattus_norvegicus/contigview?chr=8&vc_start=46824565&vc_end=46827474) | [IPI00565500.2](http://www.ensembl.org/Rattus_norvegicus/geneview?gene=IPI00565500.2) |  |
|  | [ENSRNOG00000006604](http://www.ensembl.org/Rattus_norvegicus/Gene/Summary?db=core;g=ENSRNOG00000006604) | [8](http://www.ensembl.org/Rattus_norvegicus/mapview?chr=8) | [47027721](http://www.ensembl.org/Rattus_norvegicus/contigview?chr=8&vc_start=47027721&vc_end=47031857) | [47031857](http://www.ensembl.org/Rattus_norvegicus/contigview?chr=8&vc_start=47027721&vc_end=47031857) | [THY1_RAT](http://www.ensembl.org/Rattus_norvegicus/geneview?gene=THY1_RAT) | Thy-1 membrane glycoprotein Precursor |
|  | [ENSRNOG00000006663](http://www.ensembl.org/Rattus_norvegicus/Gene/Summary?db=core;g=ENSRNOG00000006663) | [8](http://www.ensembl.org/Rattus_norvegicus/mapview?chr=8) | [47052085](http://www.ensembl.org/Rattus_norvegicus/contigview?chr=8&vc_start=47052085&vc_end=47076279) | [47076279](http://www.ensembl.org/Rattus_norvegicus/contigview?chr=8&vc_start=47052085&vc_end=47076279) | [NP_446226.2](http://www.ensembl.org/Rattus_norvegicus/geneview?gene=NP_446226.2) | ubiquitin specific peptidase 2 |
|  | [ENSRNOG00000039107](http://www.ensembl.org/Rattus_norvegicus/Gene/Summary?db=core;g=ENSRNOG00000039107) | [8](http://www.ensembl.org/Rattus_norvegicus/mapview?chr=8) | [47084053](http://www.ensembl.org/Rattus_norvegicus/contigview?chr=8&vc_start=47084053&vc_end=47088848) | [47088848](http://www.ensembl.org/Rattus_norvegicus/contigview?chr=8&vc_start=47084053&vc_end=47088848) | [NP_001101607.1](http://www.ensembl.org/Rattus_norvegicus/geneview?gene=NP_001101607.1) | membrane frizzled-related protein |
|  | [ENSRNOG00000007613](http://www.ensembl.org/Rattus_norvegicus/Gene/Summary?db=core;g=ENSRNOG00000007613) | [8](http://www.ensembl.org/Rattus_norvegicus/mapview?chr=8) | [47089564](http://www.ensembl.org/Rattus_norvegicus/contigview?chr=8&vc_start=47089564&vc_end=47091434) | [47091434](http://www.ensembl.org/Rattus_norvegicus/contigview?chr=8&vc_start=47089564&vc_end=47091434) | [C1QT5_RAT](http://www.ensembl.org/Rattus_norvegicus/geneview?gene=C1QT5_RAT) | Complement C1q tumor necrosis factor-related protein 5 Precursor |
|  | [ENSRNOG00000007726](http://www.ensembl.org/Rattus_norvegicus/Gene/Summary?db=core;g=ENSRNOG00000007726) | [8](http://www.ensembl.org/Rattus_norvegicus/mapview?chr=8) | [47121596](http://www.ensembl.org/Rattus_norvegicus/contigview?chr=8&vc_start=47121596&vc_end=47127932) | [47127932](http://www.ensembl.org/Rattus_norvegicus/contigview?chr=8&vc_start=47121596&vc_end=47127932) | [MUC18_RAT](http://www.ensembl.org/Rattus_norvegicus/geneview?gene=MUC18_RAT) | Cell surface glycoprotein MUC18 Precursor |
|  | [ENSRNOG00000039086](http://www.ensembl.org/Rattus_norvegicus/Gene/Summary?db=core;g=ENSRNOG00000039086) | [8](http://www.ensembl.org/Rattus_norvegicus/mapview?chr=8) | [47218487](http://www.ensembl.org/Rattus_norvegicus/contigview?chr=8&vc_start=47218487&vc_end=47225170) | [47225170](http://www.ensembl.org/Rattus_norvegicus/contigview?chr=8&vc_start=47218487&vc_end=47225170) | [CC153_RAT](http://www.ensembl.org/Rattus_norvegicus/geneview?gene=CC153_RAT) | Coiled-coil domain-containing protein 153 |
|  | [ENSRNOG00000009799](http://www.ensembl.org/Rattus_norvegicus/Gene/Summary?db=core;g=ENSRNOG00000009799) | [8](http://www.ensembl.org/Rattus_norvegicus/mapview?chr=8) | [47305087](http://www.ensembl.org/Rattus_norvegicus/contigview?chr=8&vc_start=47305087&vc_end=47311488) | [47311488](http://www.ensembl.org/Rattus_norvegicus/contigview?chr=8&vc_start=47305087&vc_end=47311488) | [NP_955420.1](http://www.ensembl.org/Rattus_norvegicus/geneview?gene=NP_955420.1) | N-acetylglucosaminephosphotransferase 1 |
|  | [ENSRNOG00000010386](http://www.ensembl.org/Rattus_norvegicus/Gene/Summary?db=core;g=ENSRNOG00000010386) | [8](http://www.ensembl.org/Rattus_norvegicus/mapview?chr=8) | [47312609](http://www.ensembl.org/Rattus_norvegicus/contigview?chr=8&vc_start=47312609&vc_end=47313938) | [47313938](http://www.ensembl.org/Rattus_norvegicus/contigview?chr=8&vc_start=47312609&vc_end=47313938) | [NP_001102761.1](http://www.ensembl.org/Rattus_norvegicus/geneview?gene=NP_001102761.1) | H2A histone family, member X |
|  | [ENSRNOG00000010944](http://www.ensembl.org/Rattus_norvegicus/Gene/Summary?db=core;g=ENSRNOG00000010944) | [8](http://www.ensembl.org/Rattus_norvegicus/mapview?chr=8) | [47346943](http://www.ensembl.org/Rattus_norvegicus/contigview?chr=8&vc_start=47346943&vc_end=47358866) | [47358866](http://www.ensembl.org/Rattus_norvegicus/contigview?chr=8&vc_start=47346943&vc_end=47358866) | [HYOU1_RAT](http://www.ensembl.org/Rattus_norvegicus/geneview?gene=HYOU1_RAT) | Hypoxia up-regulated protein 1 Precursor |
|  | [ENSRNOG00000011361](http://www.ensembl.org/Rattus_norvegicus/Gene/Summary?db=core;g=ENSRNOG00000011361) | [8](http://www.ensembl.org/Rattus_norvegicus/mapview?chr=8) | [47363896](http://www.ensembl.org/Rattus_norvegicus/contigview?chr=8&vc_start=47363896&vc_end=47369978) | [47369978](http://www.ensembl.org/Rattus_norvegicus/contigview?chr=8&vc_start=47363896&vc_end=47369978) | [NP_113777.2](http://www.ensembl.org/Rattus_norvegicus/geneview?gene=NP_113777.2) | solute carrier family 37, member 4 |
|  | [ENSRNOG00000027503](http://www.ensembl.org/Rattus_norvegicus/Gene/Summary?db=core;g=ENSRNOG00000027503) | [8](http://www.ensembl.org/Rattus_norvegicus/mapview?chr=8) | [47374298](http://www.ensembl.org/Rattus_norvegicus/contigview?chr=8&vc_start=47374298&vc_end=47376678) | [47376678](http://www.ensembl.org/Rattus_norvegicus/contigview?chr=8&vc_start=47374298&vc_end=47376678) | [RS25_RAT](http://www.ensembl.org/Rattus_norvegicus/geneview?gene=RS25_RAT) | 40S ribosomal protein S25 |
|  | [ENSRNOG00000012420](http://www.ensembl.org/Rattus_norvegicus/Gene/Summary?db=core;g=ENSRNOG00000012420) | [8](http://www.ensembl.org/Rattus_norvegicus/mapview?chr=8) | [47453556](http://www.ensembl.org/Rattus_norvegicus/contigview?chr=8&vc_start=47453556&vc_end=47482323) | [47482323](http://www.ensembl.org/Rattus_norvegicus/contigview?chr=8&vc_start=47453556&vc_end=47482323) | [NP_001100287.1](http://www.ensembl.org/Rattus_norvegicus/geneview?gene=NP_001100287.1) | B-cell CLL/lymphoma 9-like |
|  | [ENSRNOG00000040978](http://www.ensembl.org/Rattus_norvegicus/Gene/Summary?db=core;g=ENSRNOG00000040978) | [8](http://www.ensembl.org/Rattus_norvegicus/mapview?chr=8) | [47473720](http://www.ensembl.org/Rattus_norvegicus/contigview?chr=8&vc_start=47473720&vc_end=47473830) | [47473830](http://www.ensembl.org/Rattus_norvegicus/contigview?chr=8&vc_start=47473720&vc_end=47473830) | [SNORA32](http://www.ensembl.org/Rattus_norvegicus/geneview?gene=SNORA32) | Small nucleolar RNA SNORA32 |
|  | [ENSRNOG00000012453](http://www.ensembl.org/Rattus_norvegicus/Gene/Summary?db=core;g=ENSRNOG00000012453) | [8](http://www.ensembl.org/Rattus_norvegicus/mapview?chr=8) | [47573536](http://www.ensembl.org/Rattus_norvegicus/contigview?chr=8&vc_start=47573536&vc_end=47605957) | [47605957](http://www.ensembl.org/Rattus_norvegicus/contigview?chr=8&vc_start=47573536&vc_end=47605957) | [NP_001102762.1](http://www.ensembl.org/Rattus_norvegicus/geneview?gene=NP_001102762.1) | hypothetical protein LOC500988 |
|  | [ENSRNOG00000013049](http://www.ensembl.org/Rattus_norvegicus/Gene/Summary?db=core;g=ENSRNOG00000013049) | [8](http://www.ensembl.org/Rattus_norvegicus/mapview?chr=8) | [47631777](http://www.ensembl.org/Rattus_norvegicus/contigview?chr=8&vc_start=47631777&vc_end=47645166) | [47645166](http://www.ensembl.org/Rattus_norvegicus/contigview?chr=8&vc_start=47631777&vc_end=47645166) | [NP_001129613.1](http://www.ensembl.org/Rattus_norvegicus/geneview?gene=NP_001129613.1) | trehalase |
|  | [ENSRNOG00000013919](http://www.ensembl.org/Rattus_norvegicus/Gene/Summary?db=core;g=ENSRNOG00000013919) | [8](http://www.ensembl.org/Rattus_norvegicus/mapview?chr=8) | [47729931](http://www.ensembl.org/Rattus_norvegicus/contigview?chr=8&vc_start=47729931&vc_end=47746455) | [47746455](http://www.ensembl.org/Rattus_norvegicus/contigview?chr=8&vc_start=47729931&vc_end=47746455) | [CK060_RAT](http://www.ensembl.org/Rattus_norvegicus/geneview?gene=CK060_RAT) | UPF0360 protein C11orf60 homolog |
|  | [ENSRNOG00000034989](http://www.ensembl.org/Rattus_norvegicus/Gene/Summary?db=core;g=ENSRNOG00000034989) | [8](http://www.ensembl.org/Rattus_norvegicus/mapview?chr=8) | [47811740](http://www.ensembl.org/Rattus_norvegicus/contigview?chr=8&vc_start=47811740&vc_end=47811930) | [47811930](http://www.ensembl.org/Rattus_norvegicus/contigview?chr=8&vc_start=47811740&vc_end=47811930) | [U2](http://www.ensembl.org/Rattus_norvegicus/geneview?gene=U2) | U2 spliceosomal RNA |
|  | [ENSRNOG00000015994](http://www.ensembl.org/Rattus_norvegicus/Gene/Summary?db=core;g=ENSRNOG00000015994) | [8](http://www.ensembl.org/Rattus_norvegicus/mapview?chr=8) | [47932151](http://www.ensembl.org/Rattus_norvegicus/contigview?chr=8&vc_start=47932151&vc_end=47936744) | [47936744](http://www.ensembl.org/Rattus_norvegicus/contigview?chr=8&vc_start=47932151&vc_end=47936744) | [CD3D_RAT](http://www.ensembl.org/Rattus_norvegicus/geneview?gene=CD3D_RAT) | T-cell surface glycoprotein CD3 delta chain Precursor |
|  | [ENSRNOG00000016085](http://www.ensembl.org/Rattus_norvegicus/Gene/Summary?db=core;g=ENSRNOG00000016085) | [8](http://www.ensembl.org/Rattus_norvegicus/mapview?chr=8) | [47991599](http://www.ensembl.org/Rattus_norvegicus/contigview?chr=8&vc_start=47991599&vc_end=48003100) | [48003100](http://www.ensembl.org/Rattus_norvegicus/contigview?chr=8&vc_start=47991599&vc_end=48003100) | [NP_001100288.1](http://www.ensembl.org/Rattus_norvegicus/geneview?gene=NP_001100288.1) | myelin protein zero-like 2 |
|  | [ENSRNOG00000026753](http://www.ensembl.org/Rattus_norvegicus/Gene/Summary?db=core;g=ENSRNOG00000026753) | [8](http://www.ensembl.org/Rattus_norvegicus/mapview?chr=8) | [48004277](http://www.ensembl.org/Rattus_norvegicus/contigview?chr=8&vc_start=48004277&vc_end=48024468) | [48024468](http://www.ensembl.org/Rattus_norvegicus/contigview?chr=8&vc_start=48004277&vc_end=48024468) | [NP_001102230.1](http://www.ensembl.org/Rattus_norvegicus/geneview?gene=NP_001102230.1) | myelin protein zero-like 3 |
|  | [ENSRNOG00000026702](http://www.ensembl.org/Rattus_norvegicus/Gene/Summary?db=core;g=ENSRNOG00000026702) | [8](http://www.ensembl.org/Rattus_norvegicus/mapview?chr=8) | [48037204](http://www.ensembl.org/Rattus_norvegicus/contigview?chr=8&vc_start=48037204&vc_end=48058689) | [48058689](http://www.ensembl.org/Rattus_norvegicus/contigview?chr=8&vc_start=48037204&vc_end=48058689) | [Amica1](http://www.ensembl.org/Rattus_norvegicus/geneview?gene=Amica1) | Junctional adhesion molecule-like Precursor (mCrea7) |
|  | [ENSRNOG00000034503](http://www.ensembl.org/Rattus_norvegicus/Gene/Summary?db=core;g=ENSRNOG00000034503) | [8](http://www.ensembl.org/Rattus_norvegicus/mapview?chr=8) | [48053266](http://www.ensembl.org/Rattus_norvegicus/contigview?chr=8&vc_start=48053266&vc_end=48053371) | [48053371](http://www.ensembl.org/Rattus_norvegicus/contigview?chr=8&vc_start=48053266&vc_end=48053371) | [5S_rRNA](http://www.ensembl.org/Rattus_norvegicus/geneview?gene=5S_rRNA) | 5S ribosomal RNA |
|  | [ENSRNOG00000016221](http://www.ensembl.org/Rattus_norvegicus/Gene/Summary?db=core;g=ENSRNOG00000016221) | [8](http://www.ensembl.org/Rattus_norvegicus/mapview?chr=8) | [48068839](http://www.ensembl.org/Rattus_norvegicus/contigview?chr=8&vc_start=48068839&vc_end=48078632) | [48078632](http://www.ensembl.org/Rattus_norvegicus/contigview?chr=8&vc_start=48068839&vc_end=48078632) | [SCN2B_RAT](http://www.ensembl.org/Rattus_norvegicus/geneview?gene=SCN2B_RAT) | Sodium channel subunit beta-2 Precursor |
|  | [ENSRNOG00000026679](http://www.ensembl.org/Rattus_norvegicus/Gene/Summary?db=core;g=ENSRNOG00000026679) | [8](http://www.ensembl.org/Rattus_norvegicus/mapview?chr=8) | [48091561](http://www.ensembl.org/Rattus_norvegicus/contigview?chr=8&vc_start=48091561&vc_end=48106735) | [48106735](http://www.ensembl.org/Rattus_norvegicus/contigview?chr=8&vc_start=48091561&vc_end=48106735) | [SCN4B_RAT](http://www.ensembl.org/Rattus_norvegicus/geneview?gene=SCN4B_RAT) | Sodium channel subunit beta-4 Precursor |
|  | [ENSRNOG00000016397](http://www.ensembl.org/Rattus_norvegicus/Gene/Summary?db=core;g=ENSRNOG00000016397) | [8](http://www.ensembl.org/Rattus_norvegicus/mapview?chr=8) | [48271470](http://www.ensembl.org/Rattus_norvegicus/contigview?chr=8&vc_start=48271470&vc_end=48299406) | [48299406](http://www.ensembl.org/Rattus_norvegicus/contigview?chr=8&vc_start=48271470&vc_end=48299406) | [NP_001121000.1](http://www.ensembl.org/Rattus_norvegicus/geneview?gene=NP_001121000.1) | transmembrane protease, serine 13 |
|  | [ENSRNOG00000016412](http://www.ensembl.org/Rattus_norvegicus/Gene/Summary?db=core;g=ENSRNOG00000016412) | [8](http://www.ensembl.org/Rattus_norvegicus/mapview?chr=8) | [48323870](http://www.ensembl.org/Rattus_norvegicus/contigview?chr=8&vc_start=48323870&vc_end=48347034) | [48347034](http://www.ensembl.org/Rattus_norvegicus/contigview?chr=8&vc_start=48323870&vc_end=48347034) | [FXYD6_RAT](http://www.ensembl.org/Rattus_norvegicus/geneview?gene=FXYD6_RAT) | FXYD domain-containing ion transport regulator 6 Precursor |
|  | [ENSRNOG00000016469](http://www.ensembl.org/Rattus_norvegicus/Gene/Summary?db=core;g=ENSRNOG00000016469) | [8](http://www.ensembl.org/Rattus_norvegicus/mapview?chr=8) | [48379075](http://www.ensembl.org/Rattus_norvegicus/contigview?chr=8&vc_start=48379075&vc_end=48386259) | [48386259](http://www.ensembl.org/Rattus_norvegicus/contigview?chr=8&vc_start=48379075&vc_end=48386259) | [ATNG_RAT](http://www.ensembl.org/Rattus_norvegicus/geneview?gene=ATNG_RAT) | Sodium/potassium-transporting ATPase gamma chain |
|  | [ENSRNOG00000016502](http://www.ensembl.org/Rattus_norvegicus/Gene/Summary?db=core;g=ENSRNOG00000016502) | [8](http://www.ensembl.org/Rattus_norvegicus/mapview?chr=8) | [48406413](http://www.ensembl.org/Rattus_norvegicus/contigview?chr=8&vc_start=48406413&vc_end=48723222) | [48723222](http://www.ensembl.org/Rattus_norvegicus/contigview?chr=8&vc_start=48406413&vc_end=48723222) | [NP_001101611.1](http://www.ensembl.org/Rattus_norvegicus/geneview?gene=NP_001101611.1) | Down syndrome cell adhesion molecule-like 1 |
|  | [ENSRNOG00000016847](http://www.ensembl.org/Rattus_norvegicus/Gene/Summary?db=core;g=ENSRNOG00000016847) | [8](http://www.ensembl.org/Rattus_norvegicus/mapview?chr=8) | [48655253](http://www.ensembl.org/Rattus_norvegicus/contigview?chr=8&vc_start=48655253&vc_end=48839681) | [48839681](http://www.ensembl.org/Rattus_norvegicus/contigview?chr=8&vc_start=48655253&vc_end=48839681) | [BACE1_RAT](http://www.ensembl.org/Rattus_norvegicus/geneview?gene=BACE1_RAT) | **Beta-secretase 1 Precursor** |
|  | [ENSRNOG00000017478](http://www.ensembl.org/Rattus_norvegicus/Gene/Summary?db=core;g=ENSRNOG00000017478) | [8](http://www.ensembl.org/Rattus_norvegicus/mapview?chr=8) | [48879382](http://www.ensembl.org/Rattus_norvegicus/contigview?chr=8&vc_start=48879382&vc_end=48901943) | [48901943](http://www.ensembl.org/Rattus_norvegicus/contigview?chr=8&vc_start=48879382&vc_end=48901943) | [PCSK7_RAT](http://www.ensembl.org/Rattus_norvegicus/geneview?gene=PCSK7_RAT) | Proprotein convertase subtilisin/kexin type 7 Precursor |
|  | [ENSRNOG00000025841](http://www.ensembl.org/Rattus_norvegicus/Gene/Summary?db=core;g=ENSRNOG00000025841) | [8](http://www.ensembl.org/Rattus_norvegicus/mapview?chr=8) | [48965270](http://www.ensembl.org/Rattus_norvegicus/contigview?chr=8&vc_start=48965270&vc_end=48965609) | [48965609](http://www.ensembl.org/Rattus_norvegicus/contigview?chr=8&vc_start=48965270&vc_end=48965609) |  |  |
|  | [ENSRNOG00000043003](http://www.ensembl.org/Rattus_norvegicus/Gene/Summary?db=core;g=ENSRNOG00000043003) | [8](http://www.ensembl.org/Rattus_norvegicus/mapview?chr=8) | [48989590](http://www.ensembl.org/Rattus_norvegicus/contigview?chr=8&vc_start=48989590&vc_end=49127667) | [49127667](http://www.ensembl.org/Rattus_norvegicus/contigview?chr=8&vc_start=48989590&vc_end=49127667) | [LOC689939](http://www.ensembl.org/Rattus_norvegicus/geneview?gene=LOC689939) |  |
|  | [ENSRNOG00000038880](http://www.ensembl.org/Rattus_norvegicus/Gene/Summary?db=core;g=ENSRNOG00000038880) | [8](http://www.ensembl.org/Rattus_norvegicus/mapview?chr=8) | [49233140](http://www.ensembl.org/Rattus_norvegicus/contigview?chr=8&vc_start=49233140&vc_end=49233436) | [49233436](http://www.ensembl.org/Rattus_norvegicus/contigview?chr=8&vc_start=49233140&vc_end=49233436) | [APOA4_RAT](http://www.ensembl.org/Rattus_norvegicus/geneview?gene=APOA4_RAT) | Apolipoprotein A-IV Precursor |
|  | [ENSRNOG00000018436](http://www.ensembl.org/Rattus_norvegicus/Gene/Summary?db=core;g=ENSRNOG00000018436) | [8](http://www.ensembl.org/Rattus_norvegicus/mapview?chr=8) | [49253538](http://www.ensembl.org/Rattus_norvegicus/contigview?chr=8&vc_start=49253538&vc_end=49255591) | [49255591](http://www.ensembl.org/Rattus_norvegicus/contigview?chr=8&vc_start=49253538&vc_end=49255591) | [APOA5_RAT](http://www.ensembl.org/Rattus_norvegicus/geneview?gene=APOA5_RAT) | Apolipoprotein A-V Precursor |
|  | [ENSRNOG00000018481](http://www.ensembl.org/Rattus_norvegicus/Gene/Summary?db=core;g=ENSRNOG00000018481) | [8](http://www.ensembl.org/Rattus_norvegicus/mapview?chr=8) | [49257107](http://www.ensembl.org/Rattus_norvegicus/contigview?chr=8&vc_start=49257107&vc_end=49266681) | [49266681](http://www.ensembl.org/Rattus_norvegicus/contigview?chr=8&vc_start=49257107&vc_end=49266681) | [NP_001131118.1](http://www.ensembl.org/Rattus_norvegicus/geneview?gene=NP_001131118.1) | zinc finger protein 259 |
|  | [ENSRNOG00000018665](http://www.ensembl.org/Rattus_norvegicus/Gene/Summary?db=core;g=ENSRNOG00000018665) | [8](http://www.ensembl.org/Rattus_norvegicus/mapview?chr=8) | [49267086](http://www.ensembl.org/Rattus_norvegicus/contigview?chr=8&vc_start=49267086&vc_end=49282918) | [49282918](http://www.ensembl.org/Rattus_norvegicus/contigview?chr=8&vc_start=49267086&vc_end=49282918) | [BUD13_RAT](http://www.ensembl.org/Rattus_norvegicus/geneview?gene=BUD13_RAT) | BUD13 homolog |
|  | [ENSRNOG00000038864](http://www.ensembl.org/Rattus_norvegicus/Gene/Summary?db=core;g=ENSRNOG00000038864) | [8](http://www.ensembl.org/Rattus_norvegicus/mapview?chr=8) | [49298828](http://www.ensembl.org/Rattus_norvegicus/contigview?chr=8&vc_start=49298828&vc_end=49299113) | [49299113](http://www.ensembl.org/Rattus_norvegicus/contigview?chr=8&vc_start=49298828&vc_end=49299113) |  |  |
|  | [ENSRNOG00000018778](http://www.ensembl.org/Rattus_norvegicus/Gene/Summary?db=core;g=ENSRNOG00000018778) | [8](http://www.ensembl.org/Rattus_norvegicus/mapview?chr=8) | [50765808](http://www.ensembl.org/Rattus_norvegicus/contigview?chr=8&vc_start=50765808&vc_end=51109395) | [51109395](http://www.ensembl.org/Rattus_norvegicus/contigview?chr=8&vc_start=50765808&vc_end=51109395) | [NP_001012201.1](http://www.ensembl.org/Rattus_norvegicus/geneview?gene=NP_001012201.1) | **cell adhesion molecule 1** |
|  | [ENSRNOG00000027730](http://www.ensembl.org/Rattus_norvegicus/Gene/Summary?db=core;g=ENSRNOG00000027730) | [8](http://www.ensembl.org/Rattus_norvegicus/mapview?chr=8) | [51707474](http://www.ensembl.org/Rattus_norvegicus/contigview?chr=8&vc_start=51707474&vc_end=51708458) | [51708458](http://www.ensembl.org/Rattus_norvegicus/contigview?chr=8&vc_start=51707474&vc_end=51708458) | [NP_001128107.1](http://www.ensembl.org/Rattus_norvegicus/geneview?gene=NP_001128107.1) | hypothetical protein LOC500992 |
|  | [ENSRNOG00000005918](http://www.ensembl.org/Rattus_norvegicus/Gene/Summary?db=core;g=ENSRNOG00000005918) | [8](http://www.ensembl.org/Rattus_norvegicus/mapview?chr=8) | [51775509](http://www.ensembl.org/Rattus_norvegicus/contigview?chr=8&vc_start=51775509&vc_end=51775923) | [51775923](http://www.ensembl.org/Rattus_norvegicus/contigview?chr=8&vc_start=51775509&vc_end=51775923) | [NP_001102763.1](http://www.ensembl.org/Rattus_norvegicus/geneview?gene=NP_001102763.1) | hypothetical protein LOC500993 |
|  | [ENSRNOG00000041766](http://www.ensembl.org/Rattus_norvegicus/Gene/Summary?db=core;g=ENSRNOG00000041766) | [8](http://www.ensembl.org/Rattus_norvegicus/mapview?chr=8) | [51857526](http://www.ensembl.org/Rattus_norvegicus/contigview?chr=8&vc_start=51857526&vc_end=51857848) | [51857848](http://www.ensembl.org/Rattus_norvegicus/contigview?chr=8&vc_start=51857526&vc_end=51857848) | [7SK](http://www.ensembl.org/Rattus_norvegicus/geneview?gene=7SK) | 7SK RNA |
|  | [ENSRNOG00000007325](http://www.ensembl.org/Rattus_norvegicus/Gene/Summary?db=core;g=ENSRNOG00000007325) | [8](http://www.ensembl.org/Rattus_norvegicus/mapview?chr=8) | [52245645](http://www.ensembl.org/Rattus_norvegicus/contigview?chr=8&vc_start=52245645&vc_end=52305922) | [52305922](http://www.ensembl.org/Rattus_norvegicus/contigview?chr=8&vc_start=52245645&vc_end=52305922) | [NP_001101614.1](http://www.ensembl.org/Rattus_norvegicus/geneview?gene=NP_001101614.1) | ubiquitin specific peptidase 28 |
|  | [ENSRNOG00000007661](http://www.ensembl.org/Rattus_norvegicus/Gene/Summary?db=core;g=ENSRNOG00000007661) | [8](http://www.ensembl.org/Rattus_norvegicus/mapview?chr=8) | [52316233](http://www.ensembl.org/Rattus_norvegicus/contigview?chr=8&vc_start=52316233&vc_end=52341095) | [52341095](http://www.ensembl.org/Rattus_norvegicus/contigview?chr=8&vc_start=52316233&vc_end=52341095) | [ZW10_RAT](http://www.ensembl.org/Rattus_norvegicus/geneview?gene=ZW10_RAT) | Centromere/kinetochore protein zw10 homolog |
|  | [ENSRNOG00000008058](http://www.ensembl.org/Rattus_norvegicus/Gene/Summary?db=core;g=ENSRNOG00000008058) | [8](http://www.ensembl.org/Rattus_norvegicus/mapview?chr=8) | [52361394](http://www.ensembl.org/Rattus_norvegicus/contigview?chr=8&vc_start=52361394&vc_end=52379599) | [52379599](http://www.ensembl.org/Rattus_norvegicus/contigview?chr=8&vc_start=52361394&vc_end=52379599) | [NP_695223.1](http://www.ensembl.org/Rattus_norvegicus/geneview?gene=NP_695223.1) | transmembrane protease, serine 5 |
|  | [ENSRNOG00000041978](http://www.ensembl.org/Rattus_norvegicus/Gene/Summary?db=core;g=ENSRNOG00000041978) | [8](http://www.ensembl.org/Rattus_norvegicus/mapview?chr=8) | [52495667](http://www.ensembl.org/Rattus_norvegicus/contigview?chr=8&vc_start=52495667&vc_end=52495801) | [52495801](http://www.ensembl.org/Rattus_norvegicus/contigview?chr=8&vc_start=52495667&vc_end=52495801) | [U4](http://www.ensembl.org/Rattus_norvegicus/geneview?gene=U4) | U4 spliceosomal RNA |
|  | [ENSRNOG00000008428](http://www.ensembl.org/Rattus_norvegicus/Gene/Summary?db=core;g=ENSRNOG00000008428) | [8](http://www.ensembl.org/Rattus_norvegicus/mapview?chr=8) | [52641169](http://www.ensembl.org/Rattus_norvegicus/contigview?chr=8&vc_start=52641169&vc_end=52707749) | [52707749](http://www.ensembl.org/Rattus_norvegicus/contigview?chr=8&vc_start=52641169&vc_end=52707749) | [DRD2_RAT](http://www.ensembl.org/Rattus_norvegicus/geneview?gene=DRD2_RAT) | **D(2) dopamine receptor** |
|  | [ENSRNOG00000042408](http://www.ensembl.org/Rattus_norvegicus/Gene/Summary?db=core;g=ENSRNOG00000042408) | [8](http://www.ensembl.org/Rattus_norvegicus/mapview?chr=8) | [52837225](http://www.ensembl.org/Rattus_norvegicus/contigview?chr=8&vc_start=52837225&vc_end=52863702) | [52863702](http://www.ensembl.org/Rattus_norvegicus/contigview?chr=8&vc_start=52837225&vc_end=52863702) |  |  |
|  | [ENSRNOG00000032069](http://www.ensembl.org/Rattus_norvegicus/Gene/Summary?db=core;g=ENSRNOG00000032069) | [8](http://www.ensembl.org/Rattus_norvegicus/mapview?chr=8) | [53248315](http://www.ensembl.org/Rattus_norvegicus/contigview?chr=8&vc_start=53248315&vc_end=53249269) | [53249269](http://www.ensembl.org/Rattus_norvegicus/contigview?chr=8&vc_start=53248315&vc_end=53249269) | [IPI00559057.2](http://www.ensembl.org/Rattus_norvegicus/geneview?gene=IPI00559057.2) |  |
|  | [ENSRNOG00000024346](http://www.ensembl.org/Rattus_norvegicus/Gene/Summary?db=core;g=ENSRNOG00000024346) | [8](http://www.ensembl.org/Rattus_norvegicus/mapview?chr=8) | [53852103](http://www.ensembl.org/Rattus_norvegicus/contigview?chr=8&vc_start=53852103&vc_end=53863440) | [53863440](http://www.ensembl.org/Rattus_norvegicus/contigview?chr=8&vc_start=53852103&vc_end=53863440) | [PLET1_RAT](http://www.ensembl.org/Rattus_norvegicus/geneview?gene=PLET1_RAT) | Placenta-expressed transcript 1 protein Precursor |
|  | [ENSRNOG00000009848](http://www.ensembl.org/Rattus_norvegicus/Gene/Summary?db=core;g=ENSRNOG00000009848) | [8](http://www.ensembl.org/Rattus_norvegicus/mapview?chr=8) | [53936584](http://www.ensembl.org/Rattus_norvegicus/contigview?chr=8&vc_start=53936584&vc_end=53943230) | [53943230](http://www.ensembl.org/Rattus_norvegicus/contigview?chr=8&vc_start=53936584&vc_end=53943230) | [IL18_RAT](http://www.ensembl.org/Rattus_norvegicus/geneview?gene=IL18_RAT) | Interleukin-18 Precursor (IL-18)(Interferon-gamma-inducing factor) |
|  | [ENSRNOG00000009888](http://www.ensembl.org/Rattus_norvegicus/Gene/Summary?db=core;g=ENSRNOG00000009888) | [8](http://www.ensembl.org/Rattus_norvegicus/mapview?chr=8) | [53964693](http://www.ensembl.org/Rattus_norvegicus/contigview?chr=8&vc_start=53964693&vc_end=53966071) | [53966071](http://www.ensembl.org/Rattus_norvegicus/contigview?chr=8&vc_start=53964693&vc_end=53966071) | [TIM8B_RAT](http://www.ensembl.org/Rattus_norvegicus/geneview?gene=TIM8B_RAT) | Mitochondrial import inner membrane translocase subunit Tim8 B |
|  | [ENSRNOG00000009965](http://www.ensembl.org/Rattus_norvegicus/Gene/Summary?db=core;g=ENSRNOG00000009965) | [8](http://www.ensembl.org/Rattus_norvegicus/mapview?chr=8) | [53977251](http://www.ensembl.org/Rattus_norvegicus/contigview?chr=8&vc_start=53977251&vc_end=53985416) | [53985416](http://www.ensembl.org/Rattus_norvegicus/contigview?chr=8&vc_start=53977251&vc_end=53985416) | [Pih1d2](http://www.ensembl.org/Rattus_norvegicus/geneview?gene=Pih1d2) |  |
|  | [ENSRNOG00000010524](http://www.ensembl.org/Rattus_norvegicus/Gene/Summary?db=core;g=ENSRNOG00000010524) | [8](http://www.ensembl.org/Rattus_norvegicus/mapview?chr=8) | [54107832](http://www.ensembl.org/Rattus_norvegicus/contigview?chr=8&vc_start=54107832&vc_end=54111368) | [54111368](http://www.ensembl.org/Rattus_norvegicus/contigview?chr=8&vc_start=54107832&vc_end=54111368) | [CRYAB_RAT](http://www.ensembl.org/Rattus_norvegicus/geneview?gene=CRYAB_RAT) | Alpha-crystallin B chain |
|  | [ENSRNOG00000010743](http://www.ensembl.org/Rattus_norvegicus/Gene/Summary?db=core;g=ENSRNOG00000010743) | [8](http://www.ensembl.org/Rattus_norvegicus/mapview?chr=8) | [54125767](http://www.ensembl.org/Rattus_norvegicus/contigview?chr=8&vc_start=54125767&vc_end=54130196) | [54130196](http://www.ensembl.org/Rattus_norvegicus/contigview?chr=8&vc_start=54125767&vc_end=54130196) | [NP_001101615.1](http://www.ensembl.org/Rattus_norvegicus/geneview?gene=NP_001101615.1) | ferredoxin-fold anticodon binding domain containing 1 |
|  | [ENSRNOG00000010877](http://www.ensembl.org/Rattus_norvegicus/Gene/Summary?db=core;g=ENSRNOG00000010877) | [8](http://www.ensembl.org/Rattus_norvegicus/mapview?chr=8) | [54131722](http://www.ensembl.org/Rattus_norvegicus/contigview?chr=8&vc_start=54131722&vc_end=54194200) | [54194200](http://www.ensembl.org/Rattus_norvegicus/contigview?chr=8&vc_start=54131722&vc_end=54194200) | [NP_001102470.1](http://www.ensembl.org/Rattus_norvegicus/geneview?gene=NP_001102470.1) | asparagine-linked glycosylation 9 protein |
|  | [ENSRNOG00000010922](http://www.ensembl.org/Rattus_norvegicus/Gene/Summary?db=core;g=ENSRNOG00000010922) | [8](http://www.ensembl.org/Rattus_norvegicus/mapview?chr=8) | [54208126](http://www.ensembl.org/Rattus_norvegicus/contigview?chr=8&vc_start=54208126&vc_end=54240250) | [54240250](http://www.ensembl.org/Rattus_norvegicus/contigview?chr=8&vc_start=54208126&vc_end=54240250) | [2AAB_RAT](http://www.ensembl.org/Rattus_norvegicus/geneview?gene=2AAB_RAT) | **Serine/threonine-protein phosphatase 2A** |
|  | [ENSRNOG00000011277](http://www.ensembl.org/Rattus_norvegicus/Gene/Summary?db=core;g=ENSRNOG00000011277) | [8](http://www.ensembl.org/Rattus_norvegicus/mapview?chr=8) | [54434387](http://www.ensembl.org/Rattus_norvegicus/contigview?chr=8&vc_start=54434387&vc_end=54438122) | [54438122](http://www.ensembl.org/Rattus_norvegicus/contigview?chr=8&vc_start=54434387&vc_end=54438122) | [NP_001013194.1](http://www.ensembl.org/Rattus_norvegicus/geneview?gene=NP_001013194.1) | B-cell translocation gene 4 |
|  | [ENSRNOG00000011500](http://www.ensembl.org/Rattus_norvegicus/Gene/Summary?db=core;g=ENSRNOG00000011500) | [8](http://www.ensembl.org/Rattus_norvegicus/mapview?chr=8) | [54534417](http://www.ensembl.org/Rattus_norvegicus/contigview?chr=8&vc_start=54534417&vc_end=54561348) | [54561348](http://www.ensembl.org/Rattus_norvegicus/contigview?chr=8&vc_start=54534417&vc_end=54561348) | [NP_001103069.1](http://www.ensembl.org/Rattus_norvegicus/geneview?gene=NP_001103069.1) | POU class 2 associating factor 1 |
|  | [ENSRNOG00000025624](http://www.ensembl.org/Rattus_norvegicus/Gene/Summary?db=core;g=ENSRNOG00000025624) | [8](http://www.ensembl.org/Rattus_norvegicus/mapview?chr=8) | [55085195](http://www.ensembl.org/Rattus_norvegicus/contigview?chr=8&vc_start=55085195&vc_end=55167154) | [55167154](http://www.ensembl.org/Rattus_norvegicus/contigview?chr=8&vc_start=55085195&vc_end=55167154) | [RHG20_RAT](http://www.ensembl.org/Rattus_norvegicus/geneview?gene=RHG20_RAT) | Rho GTPase-activating protein 20 |
|  | [ENSRNOG00000012237](http://www.ensembl.org/Rattus_norvegicus/Gene/Summary?db=core;g=ENSRNOG00000012237) | [8](http://www.ensembl.org/Rattus_norvegicus/mapview?chr=8) | [55404030](http://www.ensembl.org/Rattus_norvegicus/contigview?chr=8&vc_start=55404030&vc_end=55450182) | [55450182](http://www.ensembl.org/Rattus_norvegicus/contigview?chr=8&vc_start=55404030&vc_end=55450182) | [NP_001005889.2](http://www.ensembl.org/Rattus_norvegicus/geneview?gene=NP_001005889.2) | radixin |
|  | [ENSRNOG00000040738](http://www.ensembl.org/Rattus_norvegicus/Gene/Summary?db=core;g=ENSRNOG00000040738) | [8](http://www.ensembl.org/Rattus_norvegicus/mapview?chr=8) | [55677687](http://www.ensembl.org/Rattus_norvegicus/contigview?chr=8&vc_start=55677687&vc_end=55677816) | [55677816](http://www.ensembl.org/Rattus_norvegicus/contigview?chr=8&vc_start=55677687&vc_end=55677816) | [SNORA17](http://www.ensembl.org/Rattus_norvegicus/geneview?gene=SNORA17) | Small nucleolar RNA SNORA17 |
|  | [ENSRNOG00000034955](http://www.ensembl.org/Rattus_norvegicus/Gene/Summary?db=core;g=ENSRNOG00000034955) | [8](http://www.ensembl.org/Rattus_norvegicus/mapview?chr=8) | [56177038](http://www.ensembl.org/Rattus_norvegicus/contigview?chr=8&vc_start=56177038&vc_end=56177145) | [56177145](http://www.ensembl.org/Rattus_norvegicus/contigview?chr=8&vc_start=56177038&vc_end=56177145) | [U6](http://www.ensembl.org/Rattus_norvegicus/geneview?gene=U6) | U6 spliceosomal RNA |
|  | [ENSRNOG00000008216](http://www.ensembl.org/Rattus_norvegicus/Gene/Summary?db=core;g=ENSRNOG00000008216) | [8](http://www.ensembl.org/Rattus_norvegicus/mapview?chr=8) | [56615132](http://www.ensembl.org/Rattus_norvegicus/contigview?chr=8&vc_start=56615132&vc_end=56615488) | [56615488](http://www.ensembl.org/Rattus_norvegicus/contigview?chr=8&vc_start=56615132&vc_end=56615488) |  |  |
|  | [ENSRNOG00000025115](http://www.ensembl.org/Rattus_norvegicus/Gene/Summary?db=core;g=ENSRNOG00000025115) | [8](http://www.ensembl.org/Rattus_norvegicus/mapview?chr=8) | [56800811](http://www.ensembl.org/Rattus_norvegicus/contigview?chr=8&vc_start=56800811&vc_end=56835516) | [56835516](http://www.ensembl.org/Rattus_norvegicus/contigview?chr=8&vc_start=56800811&vc_end=56835516) | [Exph5](http://www.ensembl.org/Rattus_norvegicus/geneview?gene=Exph5) |  |
|  | [ENSRNOG00000007177](http://www.ensembl.org/Rattus_norvegicus/Gene/Summary?db=core;g=ENSRNOG00000007177) | [8](http://www.ensembl.org/Rattus_norvegicus/mapview?chr=8) | [56841444](http://www.ensembl.org/Rattus_norvegicus/contigview?chr=8&vc_start=56841444&vc_end=56859056) | [56859056](http://www.ensembl.org/Rattus_norvegicus/contigview?chr=8&vc_start=56841444&vc_end=56859056) | [KDEL2_RAT](http://www.ensembl.org/Rattus_norvegicus/geneview?gene=KDEL2_RAT) | KDEL motif-containing protein 2 Precursor |
|  | [ENSRNOG00000007266](http://www.ensembl.org/Rattus_norvegicus/Gene/Summary?db=core;g=ENSRNOG00000007266) | [8](http://www.ensembl.org/Rattus_norvegicus/mapview?chr=8) | [56860023](http://www.ensembl.org/Rattus_norvegicus/contigview?chr=8&vc_start=56860023&vc_end=56888401) | [56888401](http://www.ensembl.org/Rattus_norvegicus/contigview?chr=8&vc_start=56860023&vc_end=56888401) | [CK065_RAT](http://www.ensembl.org/Rattus_norvegicus/geneview?gene=CK065_RAT) | Uncharacterized protein C11orf65 homolog |
|  | [ENSRNOG00000024934](http://www.ensembl.org/Rattus_norvegicus/Gene/Summary?db=core;g=ENSRNOG00000024934) | [8](http://www.ensembl.org/Rattus_norvegicus/mapview?chr=8) | [56997121](http://www.ensembl.org/Rattus_norvegicus/contigview?chr=8&vc_start=56997121&vc_end=57034888) | [57034888](http://www.ensembl.org/Rattus_norvegicus/contigview?chr=8&vc_start=56997121&vc_end=57034888) | [NP_001101617.1](http://www.ensembl.org/Rattus_norvegicus/geneview?gene=NP_001101617.1) | nuclear protein, ataxia-telangiectasia locus |
|  | [ENSRNOG00000035091](http://www.ensembl.org/Rattus_norvegicus/Gene/Summary?db=core;g=ENSRNOG00000035091) | [8](http://www.ensembl.org/Rattus_norvegicus/mapview?chr=8) | [57107996](http://www.ensembl.org/Rattus_norvegicus/contigview?chr=8&vc_start=57107996&vc_end=57108124) | [57108124](http://www.ensembl.org/Rattus_norvegicus/contigview?chr=8&vc_start=57107996&vc_end=57108124) | [SNORA61](http://www.ensembl.org/Rattus_norvegicus/geneview?gene=SNORA61) | Small nucleolar RNA SNORA61 |
|  | [ENSRNOG00000009014](http://www.ensembl.org/Rattus_norvegicus/Gene/Summary?db=core;g=ENSRNOG00000009014) | [8](http://www.ensembl.org/Rattus_norvegicus/mapview?chr=8) | [57230412](http://www.ensembl.org/Rattus_norvegicus/contigview?chr=8&vc_start=57230412&vc_end=57274424) | [57274424](http://www.ensembl.org/Rattus_norvegicus/contigview?chr=8&vc_start=57230412&vc_end=57274424) | [NP_001100292.1](http://www.ensembl.org/Rattus_norvegicus/geneview?gene=NP_001100292.1) | solute carrier family 35, member F2 |
|  | [ENSRNOG00000009047](http://www.ensembl.org/Rattus_norvegicus/Gene/Summary?db=core;g=ENSRNOG00000009047) | [8](http://www.ensembl.org/Rattus_norvegicus/mapview?chr=8) | [57314470](http://www.ensembl.org/Rattus_norvegicus/contigview?chr=8&vc_start=57314470&vc_end=57318717) | [57318717](http://www.ensembl.org/Rattus_norvegicus/contigview?chr=8&vc_start=57314470&vc_end=57318717) | [SARCO_RAT](http://www.ensembl.org/Rattus_norvegicus/geneview?gene=SARCO_RAT) | Sarcolipin |
| **Parameters - SERT-/-** | **Ensembl Gene ID** | **Chr.** | **Gene Start** | **Gene End** | **Gene Name** | **Description** |
| Cocaine-induced locomotor | [ENSRNOG00000024082](http://www.ensembl.org/Rattus_norvegicus/Gene/Summary?db=core;g=ENSRNOG00000024082) | [8](http://www.ensembl.org/Rattus_norvegicus/mapview?chr=8) | [57793743](http://www.ensembl.org/Rattus_norvegicus/contigview?chr=8&vc_start=57793743&vc_end=57839272) | [57839272](http://www.ensembl.org/Rattus_norvegicus/contigview?chr=8&vc_start=57793743&vc_end=57839272) | [GLDN_RAT](http://www.ensembl.org/Rattus_norvegicus/geneview?gene=GLDN_RAT) | Gliomedin |
| activity 3-120 min | [ENSRNOG00000009844](http://www.ensembl.org/Rattus_norvegicus/Gene/Summary?db=core;g=ENSRNOG00000009844) | [8](http://www.ensembl.org/Rattus_norvegicus/mapview?chr=8) | [57982743](http://www.ensembl.org/Rattus_norvegicus/contigview?chr=8&vc_start=57982743&vc_end=57984115) | [57984115](http://www.ensembl.org/Rattus_norvegicus/contigview?chr=8&vc_start=57982743&vc_end=57984115) | [IPI00370520.2](http://www.ensembl.org/Rattus_norvegicus/geneview?gene=IPI00370520.2) |  |
|  | [ENSRNOG00000023870](http://www.ensembl.org/Rattus_norvegicus/Gene/Summary?db=core;g=ENSRNOG00000023870) | [8](http://www.ensembl.org/Rattus_norvegicus/mapview?chr=8) | [58055341](http://www.ensembl.org/Rattus_norvegicus/contigview?chr=8&vc_start=58055341&vc_end=58092402) | [58092402](http://www.ensembl.org/Rattus_norvegicus/contigview?chr=8&vc_start=58055341&vc_end=58092402) | [Sh2d7](http://www.ensembl.org/Rattus_norvegicus/geneview?gene=Sh2d7) |  |
|  | [ENSRNOG00000010277](http://www.ensembl.org/Rattus_norvegicus/Gene/Summary?db=core;g=ENSRNOG00000010277) | [8](http://www.ensembl.org/Rattus_norvegicus/mapview?chr=8) | [58135412](http://www.ensembl.org/Rattus_norvegicus/contigview?chr=8&vc_start=58135412&vc_end=58155798) | [58155798](http://www.ensembl.org/Rattus_norvegicus/contigview?chr=8&vc_start=58135412&vc_end=58155798) | [IDH3A_RAT](http://www.ensembl.org/Rattus_norvegicus/geneview?gene=IDH3A_RAT) | Isocitrate dehydrogenase subunit alpha, mitochondrial Precursor |
|  | [ENSRNOG00000012106](http://www.ensembl.org/Rattus_norvegicus/Gene/Summary?db=core;g=ENSRNOG00000012106) | [8](http://www.ensembl.org/Rattus_norvegicus/mapview?chr=8) | [58250793](http://www.ensembl.org/Rattus_norvegicus/contigview?chr=8&vc_start=58250793&vc_end=58266534) | [58266534](http://www.ensembl.org/Rattus_norvegicus/contigview?chr=8&vc_start=58250793&vc_end=58266534) | [NP_001020582.1](http://www.ensembl.org/Rattus_norvegicus/geneview?gene=NP_001020582.1) | DnaJ (Hsp40) homolog, subfamily A, member 4 |
|  | [ENSRNOG00000023633](http://www.ensembl.org/Rattus_norvegicus/Gene/Summary?db=core;g=ENSRNOG00000023633) | [8](http://www.ensembl.org/Rattus_norvegicus/mapview?chr=8) | [58317337](http://www.ensembl.org/Rattus_norvegicus/contigview?chr=8&vc_start=58317337&vc_end=58325375) | [58325375](http://www.ensembl.org/Rattus_norvegicus/contigview?chr=8&vc_start=58317337&vc_end=58325375) | [RABP1_RAT](http://www.ensembl.org/Rattus_norvegicus/geneview?gene=RABP1_RAT) | Cellular retinoic acid-binding protein 1 |
|  | [ENSRNOG00000013271](http://www.ensembl.org/Rattus_norvegicus/Gene/Summary?db=core;g=ENSRNOG00000013271) | [8](http://www.ensembl.org/Rattus_norvegicus/mapview?chr=8) | [58433218](http://www.ensembl.org/Rattus_norvegicus/contigview?chr=8&vc_start=58433218&vc_end=58477305) | [58477305](http://www.ensembl.org/Rattus_norvegicus/contigview?chr=8&vc_start=58433218&vc_end=58477305) | [IREB2_RAT](http://www.ensembl.org/Rattus_norvegicus/geneview?gene=IREB2_RAT) | Iron-responsive element-binding protein 2 |
|  | [ENSRNOG00000013419](http://www.ensembl.org/Rattus_norvegicus/Gene/Summary?db=core;g=ENSRNOG00000013419) | [8](http://www.ensembl.org/Rattus_norvegicus/mapview?chr=8) | [58484177](http://www.ensembl.org/Rattus_norvegicus/contigview?chr=8&vc_start=58484177&vc_end=58505553) | [58505553](http://www.ensembl.org/Rattus_norvegicus/contigview?chr=8&vc_start=58484177&vc_end=58505553) | [NP_001100293.1](http://www.ensembl.org/Rattus_norvegicus/geneview?gene=NP_001100293.1) | aminoglycoside phosphotransferase domain containing 1 |
|  | [ENSRNOG00000013493](http://www.ensembl.org/Rattus_norvegicus/Gene/Summary?db=core;g=ENSRNOG00000013493) | [8](http://www.ensembl.org/Rattus_norvegicus/mapview?chr=8) | [58508627](http://www.ensembl.org/Rattus_norvegicus/contigview?chr=8&vc_start=58508627&vc_end=58516104) | [58516104](http://www.ensembl.org/Rattus_norvegicus/contigview?chr=8&vc_start=58508627&vc_end=58516104) | [PSA4_RAT](http://www.ensembl.org/Rattus_norvegicus/geneview?gene=PSA4_RAT) | Proteasome subunit alpha type-4 |
|  | [ENSRNOG00000013610](http://www.ensembl.org/Rattus_norvegicus/Gene/Summary?db=core;g=ENSRNOG00000013610) | [8](http://www.ensembl.org/Rattus_norvegicus/mapview?chr=8) | [58538002](http://www.ensembl.org/Rattus_norvegicus/contigview?chr=8&vc_start=58538002&vc_end=58566388) | [58566388](http://www.ensembl.org/Rattus_norvegicus/contigview?chr=8&vc_start=58538002&vc_end=58566388) | [ACHA5_RAT](http://www.ensembl.org/Rattus_norvegicus/geneview?gene=ACHA5_RAT) | **Neuronal acetylcholine receptor subunit alpha-5 Precursor** |
|  | [ENSRNOG00000014529](http://www.ensembl.org/Rattus_norvegicus/Gene/Summary?db=core;g=ENSRNOG00000014529) | [8](http://www.ensembl.org/Rattus_norvegicus/mapview?chr=8) | [58688257](http://www.ensembl.org/Rattus_norvegicus/contigview?chr=8&vc_start=58688257&vc_end=58748980) | [58748980](http://www.ensembl.org/Rattus_norvegicus/contigview?chr=8&vc_start=58688257&vc_end=58748980) | [Ube2q2](http://www.ensembl.org/Rattus_norvegicus/geneview?gene=Ube2q2) |  |
|  | [ENSRNOG00000022702](http://www.ensembl.org/Rattus_norvegicus/Gene/Summary?db=core;g=ENSRNOG00000022702) | [8](http://www.ensembl.org/Rattus_norvegicus/mapview?chr=8) | [58751584](http://www.ensembl.org/Rattus_norvegicus/contigview?chr=8&vc_start=58751584&vc_end=58767612) | [58767612](http://www.ensembl.org/Rattus_norvegicus/contigview?chr=8&vc_start=58751584&vc_end=58767612) | [NP_001032859.1](http://www.ensembl.org/Rattus_norvegicus/geneview?gene=NP_001032859.1) | F-box protein 22 |
|  | [ENSRNOG00000015166](http://www.ensembl.org/Rattus_norvegicus/Gene/Summary?db=core;g=ENSRNOG00000015166) | [8](http://www.ensembl.org/Rattus_norvegicus/mapview?chr=8) | [58868256](http://www.ensembl.org/Rattus_norvegicus/contigview?chr=8&vc_start=58868256&vc_end=58869409) | [58869409](http://www.ensembl.org/Rattus_norvegicus/contigview?chr=8&vc_start=58868256&vc_end=58869409) | [IPI00203534.3](http://www.ensembl.org/Rattus_norvegicus/geneview?gene=IPI00203534.3) |  |
|  | [ENSRNOG00000015201](http://www.ensembl.org/Rattus_norvegicus/Gene/Summary?db=core;g=ENSRNOG00000015201) | [8](http://www.ensembl.org/Rattus_norvegicus/mapview?chr=8) | [58935950](http://www.ensembl.org/Rattus_norvegicus/contigview?chr=8&vc_start=58935950&vc_end=58998905) | [58998905](http://www.ensembl.org/Rattus_norvegicus/contigview?chr=8&vc_start=58935950&vc_end=58998905) | [NP_001102764.1](http://www.ensembl.org/Rattus_norvegicus/geneview?gene=NP_001102764.1) | hypothetical protein LOC501002 |
|  | [ENSRNOG00000015336](http://www.ensembl.org/Rattus_norvegicus/Gene/Summary?db=core;g=ENSRNOG00000015336) | [8](http://www.ensembl.org/Rattus_norvegicus/mapview?chr=8) | [59105622](http://www.ensembl.org/Rattus_norvegicus/contigview?chr=8&vc_start=59105622&vc_end=59110708) | [59110708](http://www.ensembl.org/Rattus_norvegicus/contigview?chr=8&vc_start=59105622&vc_end=59110708) | [ISL2_RAT](http://www.ensembl.org/Rattus_norvegicus/geneview?gene=ISL2_RAT) | Insulin gene enhancer protein ISL-2 (Islet-2) |
|  | [ENSRNOG00000029840](http://www.ensembl.org/Rattus_norvegicus/Gene/Summary?db=core;g=ENSRNOG00000029840) | [8](http://www.ensembl.org/Rattus_norvegicus/mapview?chr=8) | [59357135](http://www.ensembl.org/Rattus_norvegicus/contigview?chr=8&vc_start=59357135&vc_end=59357590) | [59357590](http://www.ensembl.org/Rattus_norvegicus/contigview?chr=8&vc_start=59357135&vc_end=59357590) | [IPI00562174.1](http://www.ensembl.org/Rattus_norvegicus/geneview?gene=IPI00562174.1) |  |
|  | [ENSRNOG00000022382](http://www.ensembl.org/Rattus_norvegicus/Gene/Summary?db=core;g=ENSRNOG00000022382) | [8](http://www.ensembl.org/Rattus_norvegicus/mapview?chr=8) | [59551021](http://www.ensembl.org/Rattus_norvegicus/contigview?chr=8&vc_start=59551021&vc_end=59551880) | [59551880](http://www.ensembl.org/Rattus_norvegicus/contigview?chr=8&vc_start=59551021&vc_end=59551880) |  |  |
|  | [ENSRNOG00000015780](http://www.ensembl.org/Rattus_norvegicus/Gene/Summary?db=core;g=ENSRNOG00000015780) | [8](http://www.ensembl.org/Rattus_norvegicus/mapview?chr=8) | [59642838](http://www.ensembl.org/Rattus_norvegicus/contigview?chr=8&vc_start=59642838&vc_end=59659854) | [59659854](http://www.ensembl.org/Rattus_norvegicus/contigview?chr=8&vc_start=59642838&vc_end=59659854) | [RCN2_RAT](http://www.ensembl.org/Rattus_norvegicus/geneview?gene=RCN2_RAT) | Reticulocalbin-2 Precursor (Calcium-binding protein ERC-55) |
|  | [ENSRNOG00000016413](http://www.ensembl.org/Rattus_norvegicus/Gene/Summary?db=core;g=ENSRNOG00000016413) | [8](http://www.ensembl.org/Rattus_norvegicus/mapview?chr=8) | [59693306](http://www.ensembl.org/Rattus_norvegicus/contigview?chr=8&vc_start=59693306&vc_end=59732474) | [59732474](http://www.ensembl.org/Rattus_norvegicus/contigview?chr=8&vc_start=59693306&vc_end=59732474) | [NP_001100294.2](http://www.ensembl.org/Rattus_norvegicus/geneview?gene=NP_001100294.2) | proline-serine-threonine phosphatase-interacting protein 1 |
|  | [ENSRNOG00000038531](http://www.ensembl.org/Rattus_norvegicus/Gene/Summary?db=core;g=ENSRNOG00000038531) | [8](http://www.ensembl.org/Rattus_norvegicus/mapview?chr=8) | [59773245](http://www.ensembl.org/Rattus_norvegicus/contigview?chr=8&vc_start=59773245&vc_end=59773799) | [59773799](http://www.ensembl.org/Rattus_norvegicus/contigview?chr=8&vc_start=59773245&vc_end=59773799) |  |  |
|  | [ENSRNOG00000016905](http://www.ensembl.org/Rattus_norvegicus/Gene/Summary?db=core;g=ENSRNOG00000016905) | [8](http://www.ensembl.org/Rattus_norvegicus/mapview?chr=8) | [60014815](http://www.ensembl.org/Rattus_norvegicus/contigview?chr=8&vc_start=60014815&vc_end=60091988) | [60091988](http://www.ensembl.org/Rattus_norvegicus/contigview?chr=8&vc_start=60014815&vc_end=60091988) | [NP_001101620.1](http://www.ensembl.org/Rattus_norvegicus/geneview?gene=NP_001101620.1) | high mobility group 20A |
|  | [ENSRNOG00000027113](http://www.ensembl.org/Rattus_norvegicus/Gene/Summary?db=core;g=ENSRNOG00000027113) | [8](http://www.ensembl.org/Rattus_norvegicus/mapview?chr=8) | [60506308](http://www.ensembl.org/Rattus_norvegicus/contigview?chr=8&vc_start=60506308&vc_end=60506607) | [60506607](http://www.ensembl.org/Rattus_norvegicus/contigview?chr=8&vc_start=60506308&vc_end=60506607) | [IPI00779885.1](http://www.ensembl.org/Rattus_norvegicus/geneview?gene=IPI00779885.1) |  |
|  | [ENSRNOG00000017208](http://www.ensembl.org/Rattus_norvegicus/Gene/Summary?db=core;g=ENSRNOG00000017208) | [8](http://www.ensembl.org/Rattus_norvegicus/mapview?chr=8) | [60610835](http://www.ensembl.org/Rattus_norvegicus/contigview?chr=8&vc_start=60610835&vc_end=60645877) | [60645877](http://www.ensembl.org/Rattus_norvegicus/contigview?chr=8&vc_start=60610835&vc_end=60645877) | [CSPG4_RAT](http://www.ensembl.org/Rattus_norvegicus/geneview?gene=CSPG4_RAT) | Chondroitin sulfate proteoglycan 4 Precursor |
|  | [ENSRNOG00000017460](http://www.ensembl.org/Rattus_norvegicus/Gene/Summary?db=core;g=ENSRNOG00000017460) | [8](http://www.ensembl.org/Rattus_norvegicus/mapview?chr=8) | [60685389](http://www.ensembl.org/Rattus_norvegicus/contigview?chr=8&vc_start=60685389&vc_end=60686277) | [60686277](http://www.ensembl.org/Rattus_norvegicus/contigview?chr=8&vc_start=60685389&vc_end=60686277) | [NP_001101622.1](http://www.ensembl.org/Rattus_norvegicus/geneview?gene=NP_001101622.1) | IMP3, U3 small nucleolar ribonucleoprotein, homolog |
|  | [ENSRNOG00000005426](http://www.ensembl.org/Rattus_norvegicus/Gene/Summary?db=core;g=ENSRNOG00000005426) | [8](http://www.ensembl.org/Rattus_norvegicus/mapview?chr=8) | [60694018](http://www.ensembl.org/Rattus_norvegicus/contigview?chr=8&vc_start=60694018&vc_end=60726686) | [60726686](http://www.ensembl.org/Rattus_norvegicus/contigview?chr=8&vc_start=60694018&vc_end=60726686) | [SPN1_RAT](http://www.ensembl.org/Rattus_norvegicus/geneview?gene=SPN1_RAT) | Snurportin-1 (RNA U transporter 1) |
|  | [ENSRNOG00000017600](http://www.ensembl.org/Rattus_norvegicus/Gene/Summary?db=core;g=ENSRNOG00000017600) | [8](http://www.ensembl.org/Rattus_norvegicus/mapview?chr=8) | [60737741](http://www.ensembl.org/Rattus_norvegicus/contigview?chr=8&vc_start=60737741&vc_end=60817945) | [60817945](http://www.ensembl.org/Rattus_norvegicus/contigview?chr=8&vc_start=60737741&vc_end=60817945) | [PTN9_RAT](http://www.ensembl.org/Rattus_norvegicus/geneview?gene=PTN9_RAT) | **Tyrosine-protein phosphatase non-receptor type 9** |
|  | [ENSRNOG00000032254](http://www.ensembl.org/Rattus_norvegicus/Gene/Summary?db=core;g=ENSRNOG00000032254) | [8](http://www.ensembl.org/Rattus_norvegicus/mapview?chr=8) | [60843640](http://www.ensembl.org/Rattus_norvegicus/contigview?chr=8&vc_start=60843640&vc_end=60884764) | [60884764](http://www.ensembl.org/Rattus_norvegicus/contigview?chr=8&vc_start=60843640&vc_end=60884764) | [NP_001102231.1](http://www.ensembl.org/Rattus_norvegicus/geneview?gene=NP_001102231.1) | SIN3 homolog A, transcription regulator |
|  | [ENSRNOG00000030654](http://www.ensembl.org/Rattus_norvegicus/Gene/Summary?db=core;g=ENSRNOG00000030654) | [8](http://www.ensembl.org/Rattus_norvegicus/mapview?chr=8) | [60887342](http://www.ensembl.org/Rattus_norvegicus/contigview?chr=8&vc_start=60887342&vc_end=60898465) | [60898465](http://www.ensembl.org/Rattus_norvegicus/contigview?chr=8&vc_start=60887342&vc_end=60898465) | [MA2C1_RAT](http://www.ensembl.org/Rattus_norvegicus/geneview?gene=MA2C1_RAT) | Alpha-mannosidase 2C1 |
|  | [ENSRNOG00000038477](http://www.ensembl.org/Rattus_norvegicus/Gene/Summary?db=core;g=ENSRNOG00000038477) | [8](http://www.ensembl.org/Rattus_norvegicus/mapview?chr=8) | [60983145](http://www.ensembl.org/Rattus_norvegicus/contigview?chr=8&vc_start=60983145&vc_end=60992231) | [60992231](http://www.ensembl.org/Rattus_norvegicus/contigview?chr=8&vc_start=60983145&vc_end=60992231) | [IPI00390070.3](http://www.ensembl.org/Rattus_norvegicus/geneview?gene=IPI00390070.3) |  |
|  | [ENSRNOG00000018812](http://www.ensembl.org/Rattus_norvegicus/Gene/Summary?db=core;g=ENSRNOG00000018812) | [8](http://www.ensembl.org/Rattus_norvegicus/mapview?chr=8) | [61248740](http://www.ensembl.org/Rattus_norvegicus/contigview?chr=8&vc_start=61248740&vc_end=61250120) | [61250120](http://www.ensembl.org/Rattus_norvegicus/contigview?chr=8&vc_start=61248740&vc_end=61250120) | [RPP25_RAT](http://www.ensembl.org/Rattus_norvegicus/geneview?gene=RPP25_RAT) | Ribonuclease P protein subunit p25 (RNase P protein subunit p25) |
|  | [ENSRNOG00000018816](http://www.ensembl.org/Rattus_norvegicus/Gene/Summary?db=core;g=ENSRNOG00000018816) | [8](http://www.ensembl.org/Rattus_norvegicus/mapview?chr=8) | [61264971](http://www.ensembl.org/Rattus_norvegicus/contigview?chr=8&vc_start=61264971&vc_end=61276380) | [61276380](http://www.ensembl.org/Rattus_norvegicus/contigview?chr=8&vc_start=61264971&vc_end=61276380) | [COX5A_RAT](http://www.ensembl.org/Rattus_norvegicus/geneview?gene=COX5A_RAT) | Cytochrome c oxidase subunit 5A, mitochondrial Precursor |
|  | [ENSRNOG00000026622](http://www.ensembl.org/Rattus_norvegicus/Gene/Summary?db=core;g=ENSRNOG00000026622) | [8](http://www.ensembl.org/Rattus_norvegicus/mapview?chr=8) | [61286029](http://www.ensembl.org/Rattus_norvegicus/contigview?chr=8&vc_start=61286029&vc_end=61287289) | [61287289](http://www.ensembl.org/Rattus_norvegicus/contigview?chr=8&vc_start=61286029&vc_end=61287289) | [NP_001102234.1](http://www.ensembl.org/Rattus_norvegicus/geneview?gene=NP_001102234.1) | hypothetical protein LOC363070 |
|  | [ENSRNOG00000019136](http://www.ensembl.org/Rattus_norvegicus/Gene/Summary?db=core;g=ENSRNOG00000019136) | [8](http://www.ensembl.org/Rattus_norvegicus/mapview?chr=8) | [61316260](http://www.ensembl.org/Rattus_norvegicus/contigview?chr=8&vc_start=61316260&vc_end=61342885) | [61342885](http://www.ensembl.org/Rattus_norvegicus/contigview?chr=8&vc_start=61316260&vc_end=61342885) | [NP_076445.1](http://www.ensembl.org/Rattus_norvegicus/geneview?gene=NP_076445.1) | secretory carrier membrane protein 2 |
|  | [ENSRNOG00000038459](http://www.ensembl.org/Rattus_norvegicus/Gene/Summary?db=core;g=ENSRNOG00000038459) | [8](http://www.ensembl.org/Rattus_norvegicus/mapview?chr=8) | [61343722](http://www.ensembl.org/Rattus_norvegicus/contigview?chr=8&vc_start=61343722&vc_end=61348918) | [61348918](http://www.ensembl.org/Rattus_norvegicus/contigview?chr=8&vc_start=61343722&vc_end=61348918) | [Ulk3](http://www.ensembl.org/Rattus_norvegicus/geneview?gene=Ulk3) |  |
|  | [ENSRNOG00000019500](http://www.ensembl.org/Rattus_norvegicus/Gene/Summary?db=core;g=ENSRNOG00000019500) | [8](http://www.ensembl.org/Rattus_norvegicus/mapview?chr=8) | [61462207](http://www.ensembl.org/Rattus_norvegicus/contigview?chr=8&vc_start=61462207&vc_end=61468237) | [61468237](http://www.ensembl.org/Rattus_norvegicus/contigview?chr=8&vc_start=61462207&vc_end=61468237) | [CP1A1_RAT](http://www.ensembl.org/Rattus_norvegicus/geneview?gene=CP1A1_RAT) | Cytochrome P450 1A1 (CYP1A1) |
|  | [ENSRNOG00000019579](http://www.ensembl.org/Rattus_norvegicus/Gene/Summary?db=core;g=ENSRNOG00000019579) | [8](http://www.ensembl.org/Rattus_norvegicus/mapview?chr=8) | [61472272](http://www.ensembl.org/Rattus_norvegicus/contigview?chr=8&vc_start=61472272&vc_end=61516975) | [61516975](http://www.ensembl.org/Rattus_norvegicus/contigview?chr=8&vc_start=61472272&vc_end=61516975) | [Edc3](http://www.ensembl.org/Rattus_norvegicus/geneview?gene=Edc3) | Enhancer of mRNA-decapping protein 3 |
|  | [ENSRNOG00000007442](http://www.ensembl.org/Rattus_norvegicus/Gene/Summary?db=core;g=ENSRNOG00000007442) | [8](http://www.ensembl.org/Rattus_norvegicus/mapview?chr=8) | [61690384](http://www.ensembl.org/Rattus_norvegicus/contigview?chr=8&vc_start=61690384&vc_end=61707678) | [61707678](http://www.ensembl.org/Rattus_norvegicus/contigview?chr=8&vc_start=61690384&vc_end=61707678) | [NP_001004247.1](http://www.ensembl.org/Rattus_norvegicus/geneview?gene=NP_001004247.1) | ubiquitin-like 7 (bone marrow stromal cell-derived) |
|  | [ENSRNOG00000007687](http://www.ensembl.org/Rattus_norvegicus/Gene/Summary?db=core;g=ENSRNOG00000007687) | [8](http://www.ensembl.org/Rattus_norvegicus/mapview?chr=8) | [61717449](http://www.ensembl.org/Rattus_norvegicus/contigview?chr=8&vc_start=61717449&vc_end=61739529) | [61739529](http://www.ensembl.org/Rattus_norvegicus/contigview?chr=8&vc_start=61717449&vc_end=61739529) | [NP_001101623.1](http://www.ensembl.org/Rattus_norvegicus/geneview?gene=NP_001101623.1) | sema domain, GPI membrane anchor, (semaphorin) 7A |
|  | [ENSRNOG00000008074](http://www.ensembl.org/Rattus_norvegicus/Gene/Summary?db=core;g=ENSRNOG00000008074) | [8](http://www.ensembl.org/Rattus_norvegicus/mapview?chr=8) | [61793951](http://www.ensembl.org/Rattus_norvegicus/contigview?chr=8&vc_start=61793951&vc_end=61805482) | [61805482](http://www.ensembl.org/Rattus_norvegicus/contigview?chr=8&vc_start=61793951&vc_end=61805482) | [CP11A_RAT](http://www.ensembl.org/Rattus_norvegicus/geneview?gene=CP11A_RAT) | Cholesterol side-chain cleavage enzyme, mitochondrial Precursor |
|  | [ENSRNOG00000041609](http://www.ensembl.org/Rattus_norvegicus/Gene/Summary?db=core;g=ENSRNOG00000041609) | [8](http://www.ensembl.org/Rattus_norvegicus/mapview?chr=8) | [61849371](http://www.ensembl.org/Rattus_norvegicus/contigview?chr=8&vc_start=61849371&vc_end=61849481) | [61849481](http://www.ensembl.org/Rattus_norvegicus/contigview?chr=8&vc_start=61849371&vc_end=61849481) |  |  |
|  | [ENSRNOG00000008312](http://www.ensembl.org/Rattus_norvegicus/Gene/Summary?db=core;g=ENSRNOG00000008312) | [8](http://www.ensembl.org/Rattus_norvegicus/mapview?chr=8) | [61920765](http://www.ensembl.org/Rattus_norvegicus/contigview?chr=8&vc_start=61920765&vc_end=61939790) | [61939790](http://www.ensembl.org/Rattus_norvegicus/contigview?chr=8&vc_start=61920765&vc_end=61939790) | [STRA6_RAT](http://www.ensembl.org/Rattus_norvegicus/geneview?gene=STRA6_RAT) | Stimulated by retinoic acid gene 6 protein homolog |
|  | [ENSRNOG00000008530](http://www.ensembl.org/Rattus_norvegicus/Gene/Summary?db=core;g=ENSRNOG00000008530) | [8](http://www.ensembl.org/Rattus_norvegicus/mapview?chr=8) | [62052522](http://www.ensembl.org/Rattus_norvegicus/contigview?chr=8&vc_start=62052522&vc_end=62059610) | [62059610](http://www.ensembl.org/Rattus_norvegicus/contigview?chr=8&vc_start=62052522&vc_end=62059610) | [Stoml1](http://www.ensembl.org/Rattus_norvegicus/geneview?gene=Stoml1) |  |
|  | [ENSRNOG00000029377](http://www.ensembl.org/Rattus_norvegicus/Gene/Summary?db=core;g=ENSRNOG00000029377) | [8](http://www.ensembl.org/Rattus_norvegicus/mapview?chr=8) | [62132205](http://www.ensembl.org/Rattus_norvegicus/contigview?chr=8&vc_start=62132205&vc_end=62132465) | [62132465](http://www.ensembl.org/Rattus_norvegicus/contigview?chr=8&vc_start=62132205&vc_end=62132465) | [RL38_RAT](http://www.ensembl.org/Rattus_norvegicus/geneview?gene=RL38_RAT) | 60S ribosomal protein L38 |
|  | [ENSRNOG00000041887](http://www.ensembl.org/Rattus_norvegicus/Gene/Summary?db=core;g=ENSRNOG00000041887) | [8](http://www.ensembl.org/Rattus_norvegicus/mapview?chr=8) | [62243586](http://www.ensembl.org/Rattus_norvegicus/contigview?chr=8&vc_start=62243586&vc_end=62243884) | [62243884](http://www.ensembl.org/Rattus_norvegicus/contigview?chr=8&vc_start=62243586&vc_end=62243884) | [SRP_euk_arch](http://www.ensembl.org/Rattus_norvegicus/geneview?gene=SRP_euk_arch) | Eukaryotic type signal recognition particle RNA |
|  | [ENSRNOG00000026238](http://www.ensembl.org/Rattus_norvegicus/Gene/Summary?db=core;g=ENSRNOG00000026238) | [8](http://www.ensembl.org/Rattus_norvegicus/mapview?chr=8) | [62297849](http://www.ensembl.org/Rattus_norvegicus/contigview?chr=8&vc_start=62297849&vc_end=62308106) | [62308106](http://www.ensembl.org/Rattus_norvegicus/contigview?chr=8&vc_start=62297849&vc_end=62308106) | [CO059_RAT](http://www.ensembl.org/Rattus_norvegicus/geneview?gene=CO059_RAT) | UPF0583 protein C15orf59 homolog |
|  | [ENSRNOG00000009029](http://www.ensembl.org/Rattus_norvegicus/Gene/Summary?db=core;g=ENSRNOG00000009029) | [8](http://www.ensembl.org/Rattus_norvegicus/mapview?chr=8) | [62389893](http://www.ensembl.org/Rattus_norvegicus/contigview?chr=8&vc_start=62389893&vc_end=62466080) | [62466080](http://www.ensembl.org/Rattus_norvegicus/contigview?chr=8&vc_start=62389893&vc_end=62466080) | [NPTN_RAT](http://www.ensembl.org/Rattus_norvegicus/geneview?gene=NPTN_RAT) | Neuroplastin Precursor (Stromal cell-derived receptor 1) |
|  | [ENSRNOG00000009450](http://www.ensembl.org/Rattus_norvegicus/Gene/Summary?db=core;g=ENSRNOG00000009450) | [8](http://www.ensembl.org/Rattus_norvegicus/mapview?chr=8) | [62629828](http://www.ensembl.org/Rattus_norvegicus/contigview?chr=8&vc_start=62629828&vc_end=62667261) | [62667261](http://www.ensembl.org/Rattus_norvegicus/contigview?chr=8&vc_start=62629828&vc_end=62667261) | [HCN4_RAT](http://www.ensembl.org/Rattus_norvegicus/geneview?gene=HCN4_RAT) | Potassium/sodium hyperpolarization-activated channel 4 |
|  | [ENSRNOG00000026178](http://www.ensembl.org/Rattus_norvegicus/Gene/Summary?db=core;g=ENSRNOG00000026178) | [8](http://www.ensembl.org/Rattus_norvegicus/mapview?chr=8) | [63120989](http://www.ensembl.org/Rattus_norvegicus/contigview?chr=8&vc_start=63120989&vc_end=63150590) | [63150590](http://www.ensembl.org/Rattus_norvegicus/contigview?chr=8&vc_start=63120989&vc_end=63150590) | [NP_001094193.1](http://www.ensembl.org/Rattus_norvegicus/geneview?gene=NP_001094193.1) | ADP-dependent glucokinase |
|  | [ENSRNOG00000040537](http://www.ensembl.org/Rattus_norvegicus/Gene/Summary?db=core;g=ENSRNOG00000040537) | [8](http://www.ensembl.org/Rattus_norvegicus/mapview?chr=8) | [63349455](http://www.ensembl.org/Rattus_norvegicus/contigview?chr=8&vc_start=63349455&vc_end=63349582) | [63349582](http://www.ensembl.org/Rattus_norvegicus/contigview?chr=8&vc_start=63349455&vc_end=63349582) | [SNORA40](http://www.ensembl.org/Rattus_norvegicus/geneview?gene=SNORA40) | Small nucleolar RNA SNORA40 |
|  | [ENSRNOG00000010252](http://www.ensembl.org/Rattus_norvegicus/Gene/Summary?db=core;g=ENSRNOG00000010252) | [8](http://www.ensembl.org/Rattus_norvegicus/mapview?chr=8) | [63363219](http://www.ensembl.org/Rattus_norvegicus/contigview?chr=8&vc_start=63363219&vc_end=63388155) | [63388155](http://www.ensembl.org/Rattus_norvegicus/contigview?chr=8&vc_start=63363219&vc_end=63388155) | [HEXA_RAT](http://www.ensembl.org/Rattus_norvegicus/geneview?gene=HEXA_RAT) | Beta-hexosaminidase subunit alpha Precursor |
|  | [ENSRNOG00000010817](http://www.ensembl.org/Rattus_norvegicus/Gene/Summary?db=core;g=ENSRNOG00000010817) | [8](http://www.ensembl.org/Rattus_norvegicus/mapview?chr=8) | [63401862](http://www.ensembl.org/Rattus_norvegicus/contigview?chr=8&vc_start=63401862&vc_end=63433540) | [63433540](http://www.ensembl.org/Rattus_norvegicus/contigview?chr=8&vc_start=63401862&vc_end=63433540) | [NP_001100297.1](http://www.ensembl.org/Rattus_norvegicus/geneview?gene=NP_001100297.1) | bruno-like 6, RNA binding protein |
|  | [ENSRNOG00000011199](http://www.ensembl.org/Rattus_norvegicus/Gene/Summary?db=core;g=ENSRNOG00000011199) | [8](http://www.ensembl.org/Rattus_norvegicus/mapview?chr=8) | [63446638](http://www.ensembl.org/Rattus_norvegicus/contigview?chr=8&vc_start=63446638&vc_end=63477760) | [63477760](http://www.ensembl.org/Rattus_norvegicus/contigview?chr=8&vc_start=63446638&vc_end=63477760) | [NP_001100298.1](http://www.ensembl.org/Rattus_norvegicus/geneview?gene=NP_001100298.1) | poly (ADP-ribose) polymerase family, member 6 |
|  | [ENSRNOG00000035019](http://www.ensembl.org/Rattus_norvegicus/Gene/Summary?db=core;g=ENSRNOG00000035019) | [8](http://www.ensembl.org/Rattus_norvegicus/mapview?chr=8) | [63482866](http://www.ensembl.org/Rattus_norvegicus/contigview?chr=8&vc_start=63482866&vc_end=63483027) | [63483027](http://www.ensembl.org/Rattus_norvegicus/contigview?chr=8&vc_start=63482866&vc_end=63483027) | [U4](http://www.ensembl.org/Rattus_norvegicus/geneview?gene=U4) | U4 spliceosomal RNA |
|  | [ENSRNOG00000011329](http://www.ensembl.org/Rattus_norvegicus/Gene/Summary?db=core;g=ENSRNOG00000011329) | [8](http://www.ensembl.org/Rattus_norvegicus/mapview?chr=8) | [63486490](http://www.ensembl.org/Rattus_norvegicus/contigview?chr=8&vc_start=63486490&vc_end=63508016) | [63508016](http://www.ensembl.org/Rattus_norvegicus/contigview?chr=8&vc_start=63486490&vc_end=63508016) | [KPYM_RAT](http://www.ensembl.org/Rattus_norvegicus/geneview?gene=KPYM_RAT) | Pyruvate kinase isozymes M1/M2 |
|  | [ENSRNOG00000025979](http://www.ensembl.org/Rattus_norvegicus/Gene/Summary?db=core;g=ENSRNOG00000025979) | [8](http://www.ensembl.org/Rattus_norvegicus/mapview?chr=8) | [63536610](http://www.ensembl.org/Rattus_norvegicus/contigview?chr=8&vc_start=63536610&vc_end=63544554) | [63544554](http://www.ensembl.org/Rattus_norvegicus/contigview?chr=8&vc_start=63536610&vc_end=63544554) | [Gramd2](http://www.ensembl.org/Rattus_norvegicus/geneview?gene=Gramd2) |  |
|  | [ENSRNOG00000011619](http://www.ensembl.org/Rattus_norvegicus/Gene/Summary?db=core;g=ENSRNOG00000011619) | [8](http://www.ensembl.org/Rattus_norvegicus/mapview?chr=8) | [63578001](http://www.ensembl.org/Rattus_norvegicus/contigview?chr=8&vc_start=63578001&vc_end=63783805) | [63783805](http://www.ensembl.org/Rattus_norvegicus/contigview?chr=8&vc_start=63578001&vc_end=63783805) | [MYO9A_RAT](http://www.ensembl.org/Rattus_norvegicus/geneview?gene=MYO9A_RAT) | Myosin-IXa (Unconventional myosin-9a)(Myr 7) |
|  | [ENSRNOG00000041384](http://www.ensembl.org/Rattus_norvegicus/Gene/Summary?db=core;g=ENSRNOG00000041384) | [8](http://www.ensembl.org/Rattus_norvegicus/mapview?chr=8) | [64243248](http://www.ensembl.org/Rattus_norvegicus/contigview?chr=8&vc_start=64243248&vc_end=64243334) | [64243334](http://www.ensembl.org/Rattus_norvegicus/contigview?chr=8&vc_start=64243248&vc_end=64243334) |  |  |
|  | [ENSRNOG00000041101](http://www.ensembl.org/Rattus_norvegicus/Gene/Summary?db=core;g=ENSRNOG00000041101) | [8](http://www.ensembl.org/Rattus_norvegicus/mapview?chr=8) | [64340257](http://www.ensembl.org/Rattus_norvegicus/contigview?chr=8&vc_start=64340257&vc_end=64340382) | [64340382](http://www.ensembl.org/Rattus_norvegicus/contigview?chr=8&vc_start=64340257&vc_end=64340382) | [SNORA17](http://www.ensembl.org/Rattus_norvegicus/geneview?gene=SNORA17) | Small nucleolar RNA SNORA17 |
|  | [ENSRNOG00000012438](http://www.ensembl.org/Rattus_norvegicus/Gene/Summary?db=core;g=ENSRNOG00000012438) | [8](http://www.ensembl.org/Rattus_norvegicus/mapview?chr=8) | [64655008](http://www.ensembl.org/Rattus_norvegicus/contigview?chr=8&vc_start=64655008&vc_end=64676258) | [64676258](http://www.ensembl.org/Rattus_norvegicus/contigview?chr=8&vc_start=64655008&vc_end=64676258) | [NP_001101624.1](http://www.ensembl.org/Rattus_norvegicus/geneview?gene=NP_001101624.1) | La ribonucleoprotein domain family, member 6 |
|  | [ENSRNOG00000031735](http://www.ensembl.org/Rattus_norvegicus/Gene/Summary?db=core;g=ENSRNOG00000031735) | [8](http://www.ensembl.org/Rattus_norvegicus/mapview?chr=8) | [64722696](http://www.ensembl.org/Rattus_norvegicus/contigview?chr=8&vc_start=64722696&vc_end=64723229) | [64723229](http://www.ensembl.org/Rattus_norvegicus/contigview?chr=8&vc_start=64722696&vc_end=64723229) | [IPI00763171.1](http://www.ensembl.org/Rattus_norvegicus/geneview?gene=IPI00763171.1) |  |
|  | [ENSRNOG00000012868](http://www.ensembl.org/Rattus_norvegicus/Gene/Summary?db=core;g=ENSRNOG00000012868) | [8](http://www.ensembl.org/Rattus_norvegicus/mapview?chr=8) | [64730636](http://www.ensembl.org/Rattus_norvegicus/contigview?chr=8&vc_start=64730636&vc_end=64813248) | [64813248](http://www.ensembl.org/Rattus_norvegicus/contigview?chr=8&vc_start=64730636&vc_end=64813248) | [RGD1560011](http://www.ensembl.org/Rattus_norvegicus/geneview?gene=RGD1560011) |  |
| **Parameters - SERT-/-** | **Ensembl Gene ID** | **Chr.** | **Gene Start** | **Gene End** | **Gene Name** | **Description** |
| Time in shelter | [ENSRNOG00000015365](http://www.ensembl.org/Rattus_norvegicus/Gene/Summary?db=core;g=ENSRNOG00000015365) | [9](http://www.ensembl.org/Rattus_norvegicus/mapview?chr=9) | [81910656](http://www.ensembl.org/Rattus_norvegicus/contigview?chr=9&vc_start=81910656&vc_end=82036998) | [82036998](http://www.ensembl.org/Rattus_norvegicus/contigview?chr=9&vc_start=81910656&vc_end=82036998) | [NP_001129231.1](http://www.ensembl.org/Rattus_norvegicus/geneview?gene=NP_001129231.1) | collagen, type IV, alpha 3 |
| Distance moved | [ENSRNOG00000015428](http://www.ensembl.org/Rattus_norvegicus/Gene/Summary?db=core;g=ENSRNOG00000015428) | [9](http://www.ensembl.org/Rattus_norvegicus/mapview?chr=9) | [82045236](http://www.ensembl.org/Rattus_norvegicus/contigview?chr=9&vc_start=82045236&vc_end=82071132) | [82071132](http://www.ensembl.org/Rattus_norvegicus/contigview?chr=9&vc_start=82045236&vc_end=82071132) | [MFF_RAT](http://www.ensembl.org/Rattus_norvegicus/geneview?gene=MFF_RAT) | Mitochondrial fission factor |
| Mobility | [ENSRNOG00000015619](http://www.ensembl.org/Rattus_norvegicus/Gene/Summary?db=core;g=ENSRNOG00000015619) | [9](http://www.ensembl.org/Rattus_norvegicus/mapview?chr=9) | [82162722](http://www.ensembl.org/Rattus_norvegicus/contigview?chr=9&vc_start=82162722&vc_end=82214975) | [82214975](http://www.ensembl.org/Rattus_norvegicus/contigview?chr=9&vc_start=82162722&vc_end=82214975) | [AGFG1_RAT](http://www.ensembl.org/Rattus_norvegicus/geneview?gene=AGFG1_RAT) | Arf-GAP domain and FG repeats-containing protein 1 |
|  | [ENSRNOG00000015992](http://www.ensembl.org/Rattus_norvegicus/Gene/Summary?db=core;g=ENSRNOG00000015992) | [9](http://www.ensembl.org/Rattus_norvegicus/mapview?chr=9) | [82440964](http://www.ensembl.org/Rattus_norvegicus/contigview?chr=9&vc_start=82440964&vc_end=82443542) | [82443542](http://www.ensembl.org/Rattus_norvegicus/contigview?chr=9&vc_start=82440964&vc_end=82443542) | [CCL20_RAT](http://www.ensembl.org/Rattus_norvegicus/geneview?gene=CCL20_RAT) | C-C motif chemokine 20 Precursor |
|  | [ENSRNOG00000016247](http://www.ensembl.org/Rattus_norvegicus/Gene/Summary?db=core;g=ENSRNOG00000016247) | [9](http://www.ensembl.org/Rattus_norvegicus/mapview?chr=9) | [82487802](http://www.ensembl.org/Rattus_norvegicus/contigview?chr=9&vc_start=82487802&vc_end=82536603) | [82536603](http://www.ensembl.org/Rattus_norvegicus/contigview?chr=9&vc_start=82487802&vc_end=82536603) | [WDR69_RAT](http://www.ensembl.org/Rattus_norvegicus/geneview?gene=WDR69_RAT) | WD repeat-containing protein 69 |
|  | [ENSRNOG00000017053](http://www.ensembl.org/Rattus_norvegicus/Gene/Summary?db=core;g=ENSRNOG00000017053) | [9](http://www.ensembl.org/Rattus_norvegicus/mapview?chr=9) | [84085586](http://www.ensembl.org/Rattus_norvegicus/contigview?chr=9&vc_start=84085586&vc_end=84164927) | [84164927](http://www.ensembl.org/Rattus_norvegicus/contigview?chr=9&vc_start=84085586&vc_end=84164927) | [NP_001102274.1](http://www.ensembl.org/Rattus_norvegicus/geneview?gene=NP_001102274.1) | F-box protein 36 |
|  | [ENSRNOG00000022800](http://www.ensembl.org/Rattus_norvegicus/Gene/Summary?db=core;g=ENSRNOG00000022800) | [9](http://www.ensembl.org/Rattus_norvegicus/mapview?chr=9) | [84281385](http://www.ensembl.org/Rattus_norvegicus/contigview?chr=9&vc_start=84281385&vc_end=84337120) | [84337120](http://www.ensembl.org/Rattus_norvegicus/contigview?chr=9&vc_start=84281385&vc_end=84337120) | [NP_001012133.1](http://www.ensembl.org/Rattus_norvegicus/geneview?gene=NP_001012133.1) | SP140 nuclear body protein |
|  | [ENSRNOG00000022769](http://www.ensembl.org/Rattus_norvegicus/Gene/Summary?db=core;g=ENSRNOG00000022769) | [9](http://www.ensembl.org/Rattus_norvegicus/mapview?chr=9) | [84374288](http://www.ensembl.org/Rattus_norvegicus/contigview?chr=9&vc_start=84374288&vc_end=84409970) | [84409970](http://www.ensembl.org/Rattus_norvegicus/contigview?chr=9&vc_start=84374288&vc_end=84409970) | [NP_001014242.1](http://www.ensembl.org/Rattus_norvegicus/geneview?gene=NP_001014242.1) | SP100 nuclear antigen |
|  | [ENSRNOG00000017297](http://www.ensembl.org/Rattus_norvegicus/Gene/Summary?db=core;g=ENSRNOG00000017297) | [9](http://www.ensembl.org/Rattus_norvegicus/mapview?chr=9) | [84528678](http://www.ensembl.org/Rattus_norvegicus/contigview?chr=9&vc_start=84528678&vc_end=84590375) | [84590375](http://www.ensembl.org/Rattus_norvegicus/contigview?chr=9&vc_start=84528678&vc_end=84590375) | [NP_001100394.1](http://www.ensembl.org/Rattus_norvegicus/geneview?gene=NP_001100394.1) | calcium binding protein 39 |
|  | [ENSRNOG00000017359](http://www.ensembl.org/Rattus_norvegicus/Gene/Summary?db=core;g=ENSRNOG00000017359) | [9](http://www.ensembl.org/Rattus_norvegicus/mapview?chr=9) | [84611756](http://www.ensembl.org/Rattus_norvegicus/contigview?chr=9&vc_start=84611756&vc_end=84625364) | [84625364](http://www.ensembl.org/Rattus_norvegicus/contigview?chr=9&vc_start=84611756&vc_end=84625364) | [ITM2C_RAT](http://www.ensembl.org/Rattus_norvegicus/geneview?gene=ITM2C_RAT) | Integral membrane protein 2C |
|  | [ENSRNOG00000017540](http://www.ensembl.org/Rattus_norvegicus/Gene/Summary?db=core;g=ENSRNOG00000017540) | [9](http://www.ensembl.org/Rattus_norvegicus/mapview?chr=9) | [84726151](http://www.ensembl.org/Rattus_norvegicus/contigview?chr=9&vc_start=84726151&vc_end=84738071) | [84738071](http://www.ensembl.org/Rattus_norvegicus/contigview?chr=9&vc_start=84726151&vc_end=84738071) | [NP_001102275.1](http://www.ensembl.org/Rattus_norvegicus/geneview?gene=NP_001102275.1) | spermatogenesis associated 3 |
|  | [ENSRNOG00000017557](http://www.ensembl.org/Rattus_norvegicus/Gene/Summary?db=core;g=ENSRNOG00000017557) | [9](http://www.ensembl.org/Rattus_norvegicus/mapview?chr=9) | [84789661](http://www.ensembl.org/Rattus_norvegicus/contigview?chr=9&vc_start=84789661&vc_end=84795335) | [84795335](http://www.ensembl.org/Rattus_norvegicus/contigview?chr=9&vc_start=84789661&vc_end=84795335) | [IPI00188569.1](http://www.ensembl.org/Rattus_norvegicus/geneview?gene=IPI00188569.1) |  |
|  | [ENSRNOG00000017730](http://www.ensembl.org/Rattus_norvegicus/Gene/Summary?db=core;g=ENSRNOG00000017730) | [9](http://www.ensembl.org/Rattus_norvegicus/mapview?chr=9) | [84807819](http://www.ensembl.org/Rattus_norvegicus/contigview?chr=9&vc_start=84807819&vc_end=84882936) | [84882936](http://www.ensembl.org/Rattus_norvegicus/contigview?chr=9&vc_start=84807819&vc_end=84882936) | [PSMD1_RAT](http://www.ensembl.org/Rattus_norvegicus/geneview?gene=PSMD1_RAT) | 26S proteasome non-ATPase regulatory subunit 1 |
|  | [ENSRNOG00000025418](http://www.ensembl.org/Rattus_norvegicus/Gene/Summary?db=core;g=ENSRNOG00000025418) | [9](http://www.ensembl.org/Rattus_norvegicus/mapview?chr=9) | [84949896](http://www.ensembl.org/Rattus_norvegicus/contigview?chr=9&vc_start=84949896&vc_end=85040301) | [85040301](http://www.ensembl.org/Rattus_norvegicus/contigview?chr=9&vc_start=84949896&vc_end=85040301) | [NP_001103133.1](http://www.ensembl.org/Rattus_norvegicus/geneview?gene=NP_001103133.1) | armadillo repeat containing 9 |
|  | [ENSRNOG00000018267](http://www.ensembl.org/Rattus_norvegicus/Gene/Summary?db=core;g=ENSRNOG00000018267) | [9](http://www.ensembl.org/Rattus_norvegicus/mapview?chr=9) | [85069241](http://www.ensembl.org/Rattus_norvegicus/contigview?chr=9&vc_start=85069241&vc_end=85073191) | [85073191](http://www.ensembl.org/Rattus_norvegicus/contigview?chr=9&vc_start=85069241&vc_end=85073191) | [B3GN7_RAT](http://www.ensembl.org/Rattus_norvegicus/geneview?gene=B3GN7_RAT) | UDP-GlcNAc:betaGal beta-1,3-N-acetylglucosaminyltransferase 7 |
|  | [ENSRNOG00000018584](http://www.ensembl.org/Rattus_norvegicus/Gene/Summary?db=core;g=ENSRNOG00000018584) | [9](http://www.ensembl.org/Rattus_norvegicus/mapview?chr=9) | [85291183](http://www.ensembl.org/Rattus_norvegicus/contigview?chr=9&vc_start=85291183&vc_end=85295244) | [85295244](http://www.ensembl.org/Rattus_norvegicus/contigview?chr=9&vc_start=85291183&vc_end=85295244) | [PTMA_RAT](http://www.ensembl.org/Rattus_norvegicus/geneview?gene=PTMA_RAT) | Prothymosin alpha |
|  | [ENSRNOG00000018723](http://www.ensembl.org/Rattus_norvegicus/Gene/Summary?db=core;g=ENSRNOG00000018723) | [9](http://www.ensembl.org/Rattus_norvegicus/mapview?chr=9) | [85353285](http://www.ensembl.org/Rattus_norvegicus/contigview?chr=9&vc_start=85353285&vc_end=85378872) | [85378872](http://www.ensembl.org/Rattus_norvegicus/contigview?chr=9&vc_start=85353285&vc_end=85378872) | [NP_001102277.1](http://www.ensembl.org/Rattus_norvegicus/geneview?gene=NP_001102277.1) | COP9 constitutive photomorphogenic homolog subunit 7B |
|  | [ENSRNOG00000018931](http://www.ensembl.org/Rattus_norvegicus/Gene/Summary?db=core;g=ENSRNOG00000018931) | [9](http://www.ensembl.org/Rattus_norvegicus/mapview?chr=9) | [85470810](http://www.ensembl.org/Rattus_norvegicus/contigview?chr=9&vc_start=85470810&vc_end=85856248) | [85856248](http://www.ensembl.org/Rattus_norvegicus/contigview?chr=9&vc_start=85470810&vc_end=85856248) | [NP_001102477.1](http://www.ensembl.org/Rattus_norvegicus/geneview?gene=NP_001102477.1) | DIS3 mitotic control homolog (S. cerevisiae)-like 2 |
|  | [ENSRNOG00000042889](http://www.ensembl.org/Rattus_norvegicus/Gene/Summary?db=core;g=ENSRNOG00000042889) | [9](http://www.ensembl.org/Rattus_norvegicus/mapview?chr=9) | [85873714](http://www.ensembl.org/Rattus_norvegicus/contigview?chr=9&vc_start=85873714&vc_end=85876375) | [85876375](http://www.ensembl.org/Rattus_norvegicus/contigview?chr=9&vc_start=85873714&vc_end=85876375) | [RGD1565100](http://www.ensembl.org/Rattus_norvegicus/geneview?gene=RGD1565100) |  |
|  | [ENSRNOG00000019375](http://www.ensembl.org/Rattus_norvegicus/Gene/Summary?db=core;g=ENSRNOG00000019375) | [9](http://www.ensembl.org/Rattus_norvegicus/mapview?chr=9) | [85924232](http://www.ensembl.org/Rattus_norvegicus/contigview?chr=9&vc_start=85924232&vc_end=85927327) | [85927327](http://www.ensembl.org/Rattus_norvegicus/contigview?chr=9&vc_start=85924232&vc_end=85927327) | [PPBJ_RAT](http://www.ensembl.org/Rattus_norvegicus/geneview?gene=PPBJ_RAT) | Intestinal alkaline phosphatase 2 Precursor |
|  | [ENSRNOG00000019634](http://www.ensembl.org/Rattus_norvegicus/Gene/Summary?db=core;g=ENSRNOG00000019634) | [9](http://www.ensembl.org/Rattus_norvegicus/mapview?chr=9) | [85944690](http://www.ensembl.org/Rattus_norvegicus/contigview?chr=9&vc_start=85944690&vc_end=86036396) | [86036396](http://www.ensembl.org/Rattus_norvegicus/contigview?chr=9&vc_start=85944690&vc_end=86036396) | [NP_001102278.1](http://www.ensembl.org/Rattus_norvegicus/geneview?gene=NP_001102278.1) | eukaryotic translation initiation factor 4E member 2 |
|  | [ENSRNOG00000029865](http://www.ensembl.org/Rattus_norvegicus/Gene/Summary?db=core;g=ENSRNOG00000029865) | [9](http://www.ensembl.org/Rattus_norvegicus/mapview?chr=9) | [85988860](http://www.ensembl.org/Rattus_norvegicus/contigview?chr=9&vc_start=85988860&vc_end=85993978) | [85993978](http://www.ensembl.org/Rattus_norvegicus/contigview?chr=9&vc_start=85988860&vc_end=85993978) | [NP_001128058.1](http://www.ensembl.org/Rattus_norvegicus/geneview?gene=NP_001128058.1) | hypothetical protein LOC363274 |
|  | [ENSRNOG00000019527](http://www.ensembl.org/Rattus_norvegicus/Gene/Summary?db=core;g=ENSRNOG00000019527) | [9](http://www.ensembl.org/Rattus_norvegicus/mapview?chr=9) | [85996421](http://www.ensembl.org/Rattus_norvegicus/contigview?chr=9&vc_start=85996421&vc_end=86004839) | [86004839](http://www.ensembl.org/Rattus_norvegicus/contigview?chr=9&vc_start=85996421&vc_end=86004839) | [ACHD_RAT](http://www.ensembl.org/Rattus_norvegicus/geneview?gene=ACHD_RAT) | **Acetylcholine receptor subunit delta Precursor** |
|  | [ENSRNOG00000019602](http://www.ensembl.org/Rattus_norvegicus/Gene/Summary?db=core;g=ENSRNOG00000019602) | [9](http://www.ensembl.org/Rattus_norvegicus/mapview?chr=9) | [86012089](http://www.ensembl.org/Rattus_norvegicus/contigview?chr=9&vc_start=86012089&vc_end=86018199) | [86018199](http://www.ensembl.org/Rattus_norvegicus/contigview?chr=9&vc_start=86012089&vc_end=86018199) | [ACHG_RAT](http://www.ensembl.org/Rattus_norvegicus/geneview?gene=ACHG_RAT) | **Acetylcholine receptor subunit gamma Precursor** |
|  | [ENSRNOG00000015596](http://www.ensembl.org/Rattus_norvegicus/Gene/Summary?db=core;g=ENSRNOG00000015596) | [9](http://www.ensembl.org/Rattus_norvegicus/mapview?chr=9) | [86076009](http://www.ensembl.org/Rattus_norvegicus/contigview?chr=9&vc_start=86076009&vc_end=86124552) | [86124552](http://www.ensembl.org/Rattus_norvegicus/contigview?chr=9&vc_start=86076009&vc_end=86124552) | [NP_001102780.1](http://www.ensembl.org/Rattus_norvegicus/geneview?gene=NP_001102780.1) | EF-hand domain family, member D1 |
|  | [ENSRNOG00000023577](http://www.ensembl.org/Rattus_norvegicus/Gene/Summary?db=core;g=ENSRNOG00000023577) | [9](http://www.ensembl.org/Rattus_norvegicus/mapview?chr=9) | [86143332](http://www.ensembl.org/Rattus_norvegicus/contigview?chr=9&vc_start=86143332&vc_end=86272080) | [86272080](http://www.ensembl.org/Rattus_norvegicus/contigview?chr=9&vc_start=86143332&vc_end=86272080) | [B2RYE6_RAT](http://www.ensembl.org/Rattus_norvegicus/geneview?gene=B2RYE6_RAT) | Putative uncharacterized protein |
|  | [ENSRNOG00000016606](http://www.ensembl.org/Rattus_norvegicus/Gene/Summary?db=core;g=ENSRNOG00000016606) | [9](http://www.ensembl.org/Rattus_norvegicus/mapview?chr=9) | [86287756](http://www.ensembl.org/Rattus_norvegicus/contigview?chr=9&vc_start=86287756&vc_end=86293229) | [86293229](http://www.ensembl.org/Rattus_norvegicus/contigview?chr=9&vc_start=86287756&vc_end=86293229) | [NP_001128059.1](http://www.ensembl.org/Rattus_norvegicus/geneview?gene=NP_001128059.1) | hypothetical protein LOC363276 |
|  | [ENSRNOG00000016962](http://www.ensembl.org/Rattus_norvegicus/Gene/Summary?db=core;g=ENSRNOG00000016962) | [9](http://www.ensembl.org/Rattus_norvegicus/mapview?chr=9) | [86553196](http://www.ensembl.org/Rattus_norvegicus/contigview?chr=9&vc_start=86553196&vc_end=86556411) | [86556411](http://www.ensembl.org/Rattus_norvegicus/contigview?chr=9&vc_start=86553196&vc_end=86556411) | [NEUR2_RAT](http://www.ensembl.org/Rattus_norvegicus/geneview?gene=NEUR2_RAT) | Sialidase-2 |
|  | [ENSRNOG00000017020](http://www.ensembl.org/Rattus_norvegicus/Gene/Summary?db=core;g=ENSRNOG00000017020) | [9](http://www.ensembl.org/Rattus_norvegicus/mapview?chr=9) | [86576739](http://www.ensembl.org/Rattus_norvegicus/contigview?chr=9&vc_start=86576739&vc_end=86682238) | [86682238](http://www.ensembl.org/Rattus_norvegicus/contigview?chr=9&vc_start=86576739&vc_end=86682238) | [SHIP1_RAT](http://www.ensembl.org/Rattus_norvegicus/geneview?gene=SHIP1_RAT) | **Phosphatidylinositol-3,4,5-trisphosphate 5-phosphatase 1** |
|  | [ENSRNOG00000017913](http://www.ensembl.org/Rattus_norvegicus/Gene/Summary?db=core;g=ENSRNOG00000017913) | [9](http://www.ensembl.org/Rattus_norvegicus/mapview?chr=9) | [86712122](http://www.ensembl.org/Rattus_norvegicus/contigview?chr=9&vc_start=86712122&vc_end=86747370) | [86747370](http://www.ensembl.org/Rattus_norvegicus/contigview?chr=9&vc_start=86712122&vc_end=86747370) | [NP_001102279.2](http://www.ensembl.org/Rattus_norvegicus/geneview?gene=NP_001102279.2) | ATG16 autophagy related 16-like 1 |
|  | [ENSRNOG00000018185](http://www.ensembl.org/Rattus_norvegicus/Gene/Summary?db=core;g=ENSRNOG00000018185) | [9](http://www.ensembl.org/Rattus_norvegicus/mapview?chr=9) | [86759933](http://www.ensembl.org/Rattus_norvegicus/contigview?chr=9&vc_start=86759933&vc_end=86799768) | [86799768](http://www.ensembl.org/Rattus_norvegicus/contigview?chr=9&vc_start=86759933&vc_end=86799768) | [ARRS_RAT](http://www.ensembl.org/Rattus_norvegicus/geneview?gene=ARRS_RAT) | S-arrestin (Retinal S-antigen) |
|  | [ENSRNOG00000023238](http://www.ensembl.org/Rattus_norvegicus/Gene/Summary?db=core;g=ENSRNOG00000023238) | [9](http://www.ensembl.org/Rattus_norvegicus/mapview?chr=9) | [86837294](http://www.ensembl.org/Rattus_norvegicus/contigview?chr=9&vc_start=86837294&vc_end=86895699) | [86895699](http://www.ensembl.org/Rattus_norvegicus/contigview?chr=9&vc_start=86837294&vc_end=86895699) | [RGD1563309](http://www.ensembl.org/Rattus_norvegicus/geneview?gene=RGD1563309) |  |
|  | [ENSRNOG00000018740](http://www.ensembl.org/Rattus_norvegicus/Gene/Summary?db=core;g=ENSRNOG00000018740) | [9](http://www.ensembl.org/Rattus_norvegicus/mapview?chr=9) | [86986915](http://www.ensembl.org/Rattus_norvegicus/contigview?chr=9&vc_start=86986915&vc_end=87098362) | [87098362](http://www.ensembl.org/Rattus_norvegicus/contigview?chr=9&vc_start=86986915&vc_end=87098362) | [UD11_RAT](http://www.ensembl.org/Rattus_norvegicus/geneview?gene=UD11_RAT) | UDP-glucuronosyltransferase 1-1 Precursor |
|  | [ENSRNOG00000042182](http://www.ensembl.org/Rattus_norvegicus/Gene/Summary?db=core;g=ENSRNOG00000042182) | [9](http://www.ensembl.org/Rattus_norvegicus/mapview?chr=9) | [87107463](http://www.ensembl.org/Rattus_norvegicus/contigview?chr=9&vc_start=87107463&vc_end=87128176) | [87128176](http://www.ensembl.org/Rattus_norvegicus/contigview?chr=9&vc_start=87107463&vc_end=87128176) | [IPI00363190.4](http://www.ensembl.org/Rattus_norvegicus/geneview?gene=IPI00363190.4) |  |
|  | [ENSRNOG00000022918](http://www.ensembl.org/Rattus_norvegicus/Gene/Summary?db=core;g=ENSRNOG00000022918) | [9](http://www.ensembl.org/Rattus_norvegicus/mapview?chr=9) | [87131195](http://www.ensembl.org/Rattus_norvegicus/contigview?chr=9&vc_start=87131195&vc_end=87141046) | [87141046](http://www.ensembl.org/Rattus_norvegicus/contigview?chr=9&vc_start=87131195&vc_end=87141046) | [IPI00558196.2](http://www.ensembl.org/Rattus_norvegicus/geneview?gene=IPI00558196.2) |  |
|  | [ENSRNOG00000019035](http://www.ensembl.org/Rattus_norvegicus/Gene/Summary?db=core;g=ENSRNOG00000019035) | [9](http://www.ensembl.org/Rattus_norvegicus/mapview?chr=9) | [87193777](http://www.ensembl.org/Rattus_norvegicus/contigview?chr=9&vc_start=87193777&vc_end=87278430) | [87278430](http://www.ensembl.org/Rattus_norvegicus/contigview?chr=9&vc_start=87193777&vc_end=87278430) | [TRPM8_RAT](http://www.ensembl.org/Rattus_norvegicus/geneview?gene=TRPM8_RAT) | Transient receptor potential cation channel subfamily M member 8 |
|  | [ENSRNOG00000019224](http://www.ensembl.org/Rattus_norvegicus/Gene/Summary?db=core;g=ENSRNOG00000019224) | [9](http://www.ensembl.org/Rattus_norvegicus/mapview?chr=9) | [87297052](http://www.ensembl.org/Rattus_norvegicus/contigview?chr=9&vc_start=87297052&vc_end=87316545) | [87316545](http://www.ensembl.org/Rattus_norvegicus/contigview?chr=9&vc_start=87297052&vc_end=87316545) | [SPP24_RAT](http://www.ensembl.org/Rattus_norvegicus/geneview?gene=SPP24_RAT) | Secreted phosphoprotein 24 Precursor |
|  | [ENSRNOG00000019316](http://www.ensembl.org/Rattus_norvegicus/Gene/Summary?db=core;g=ENSRNOG00000019316) | [9](http://www.ensembl.org/Rattus_norvegicus/mapview?chr=9) | [88227340](http://www.ensembl.org/Rattus_norvegicus/contigview?chr=9&vc_start=88227340&vc_end=88243399) | [88243399](http://www.ensembl.org/Rattus_norvegicus/contigview?chr=9&vc_start=88227340&vc_end=88243399) | [SH3B4_RAT](http://www.ensembl.org/Rattus_norvegicus/geneview?gene=SH3B4_RAT) | SH3 domain-binding protein 4 |
|  | [ENSRNOG00000019476](http://www.ensembl.org/Rattus_norvegicus/Gene/Summary?db=core;g=ENSRNOG00000019476) | [9](http://www.ensembl.org/Rattus_norvegicus/mapview?chr=9) | [88711407](http://www.ensembl.org/Rattus_norvegicus/contigview?chr=9&vc_start=88711407&vc_end=88994380) | [88994380](http://www.ensembl.org/Rattus_norvegicus/contigview?chr=9&vc_start=88711407&vc_end=88994380) | [NP_001101700.1](http://www.ensembl.org/Rattus_norvegicus/geneview?gene=NP_001101700.1) | centaurin, gamma 2 |
|  | [ENSRNOG00000019622](http://www.ensembl.org/Rattus_norvegicus/Gene/Summary?db=core;g=ENSRNOG00000019622) | [9](http://www.ensembl.org/Rattus_norvegicus/mapview?chr=9) | [89343528](http://www.ensembl.org/Rattus_norvegicus/contigview?chr=9&vc_start=89343528&vc_end=89355049) | [89355049](http://www.ensembl.org/Rattus_norvegicus/contigview?chr=9&vc_start=89343528&vc_end=89355049) | [CXCR7_RAT](http://www.ensembl.org/Rattus_norvegicus/geneview?gene=CXCR7_RAT) | C-X-C chemokine receptor type 7 |
|  | [ENSRNOG00000019635](http://www.ensembl.org/Rattus_norvegicus/Gene/Summary?db=core;g=ENSRNOG00000019635) | [9](http://www.ensembl.org/Rattus_norvegicus/mapview?chr=9) | [89802457](http://www.ensembl.org/Rattus_norvegicus/contigview?chr=9&vc_start=89802457&vc_end=89812293) | [89812293](http://www.ensembl.org/Rattus_norvegicus/contigview?chr=9&vc_start=89802457&vc_end=89812293) | [CSN8_RAT](http://www.ensembl.org/Rattus_norvegicus/geneview?gene=CSN8_RAT) | COP9 signalosome complex subunit 8 |
|  | [ENSRNOG00000019763](http://www.ensembl.org/Rattus_norvegicus/Gene/Summary?db=core;g=ENSRNOG00000019763) | [9](http://www.ensembl.org/Rattus_norvegicus/mapview?chr=9) | [90113308](http://www.ensembl.org/Rattus_norvegicus/contigview?chr=9&vc_start=90113308&vc_end=90149113) | [90149113](http://www.ensembl.org/Rattus_norvegicus/contigview?chr=9&vc_start=90113308&vc_end=90149113) | [NP_001012135.1](http://www.ensembl.org/Rattus_norvegicus/geneview?gene=NP_001012135.1) | melanophilin |
|  | [ENSRNOG00000019871](http://www.ensembl.org/Rattus_norvegicus/Gene/Summary?db=core;g=ENSRNOG00000019871) | [9](http://www.ensembl.org/Rattus_norvegicus/mapview?chr=9) | [90154031](http://www.ensembl.org/Rattus_norvegicus/contigview?chr=9&vc_start=90154031&vc_end=90154963) | [90154963](http://www.ensembl.org/Rattus_norvegicus/contigview?chr=9&vc_start=90154031&vc_end=90154963) | [PRRP_RAT](http://www.ensembl.org/Rattus_norvegicus/geneview?gene=PRRP_RAT) | Prolactin-releasing peptide Precursor |
|  | [ENSRNOG00000019892](http://www.ensembl.org/Rattus_norvegicus/Gene/Summary?db=core;g=ENSRNOG00000019892) | [9](http://www.ensembl.org/Rattus_norvegicus/mapview?chr=9) | [90278891](http://www.ensembl.org/Rattus_norvegicus/contigview?chr=9&vc_start=90278891&vc_end=90356660) | [90356660](http://www.ensembl.org/Rattus_norvegicus/contigview?chr=9&vc_start=90278891&vc_end=90356660) | [LRRF1_RAT](http://www.ensembl.org/Rattus_norvegicus/geneview?gene=LRRF1_RAT) | Leucine-rich repeat flightless-interacting protein 1 |
|  | [ENSRNOG00000024878](http://www.ensembl.org/Rattus_norvegicus/Gene/Summary?db=core;g=ENSRNOG00000024878) | [9](http://www.ensembl.org/Rattus_norvegicus/mapview?chr=9) | [90372335](http://www.ensembl.org/Rattus_norvegicus/contigview?chr=9&vc_start=90372335&vc_end=90393000) | [90393000](http://www.ensembl.org/Rattus_norvegicus/contigview?chr=9&vc_start=90372335&vc_end=90393000) | [Rbm44](http://www.ensembl.org/Rattus_norvegicus/geneview?gene=Rbm44) |  |
|  | [ENSRNOG00000019926](http://www.ensembl.org/Rattus_norvegicus/Gene/Summary?db=core;g=ENSRNOG00000019926) | [9](http://www.ensembl.org/Rattus_norvegicus/mapview?chr=9) | [90402493](http://www.ensembl.org/Rattus_norvegicus/contigview?chr=9&vc_start=90402493&vc_end=90450038) | [90450038](http://www.ensembl.org/Rattus_norvegicus/contigview?chr=9&vc_start=90402493&vc_end=90450038) | [RAMP1_RAT](http://www.ensembl.org/Rattus_norvegicus/geneview?gene=RAMP1_RAT) | Receptor activity-modifying protein 1 Precursor |
|  | [ENSRNOG00000019953](http://www.ensembl.org/Rattus_norvegicus/Gene/Summary?db=core;g=ENSRNOG00000019953) | [9](http://www.ensembl.org/Rattus_norvegicus/mapview?chr=9) | [90480334](http://www.ensembl.org/Rattus_norvegicus/contigview?chr=9&vc_start=90480334&vc_end=90515909) | [90515909](http://www.ensembl.org/Rattus_norvegicus/contigview?chr=9&vc_start=90480334&vc_end=90515909) | [UBE2F_RAT](http://www.ensembl.org/Rattus_norvegicus/geneview?gene=UBE2F_RAT) | NEDD8-conjugating enzyme UBE2F |
|  | [ENSRNOG00000020083](http://www.ensembl.org/Rattus_norvegicus/Gene/Summary?db=core;g=ENSRNOG00000020083) | [9](http://www.ensembl.org/Rattus_norvegicus/mapview?chr=9) | [90525575](http://www.ensembl.org/Rattus_norvegicus/contigview?chr=9&vc_start=90525575&vc_end=90546158) | [90546158](http://www.ensembl.org/Rattus_norvegicus/contigview?chr=9&vc_start=90525575&vc_end=90546158) | [SCLY_RAT](http://www.ensembl.org/Rattus_norvegicus/geneview?gene=SCLY_RAT) | Selenocysteine lyase |
|  | [ENSRNOG00000024717](http://www.ensembl.org/Rattus_norvegicus/Gene/Summary?db=core;g=ENSRNOG00000024717) | [9](http://www.ensembl.org/Rattus_norvegicus/mapview?chr=9) | [90547266](http://www.ensembl.org/Rattus_norvegicus/contigview?chr=9&vc_start=90547266&vc_end=90570761) | [90570761](http://www.ensembl.org/Rattus_norvegicus/contigview?chr=9&vc_start=90547266&vc_end=90570761) | [Espnl](http://www.ensembl.org/Rattus_norvegicus/geneview?gene=Espnl) |  |
|  | [ENSRNOG00000020105](http://www.ensembl.org/Rattus_norvegicus/Gene/Summary?db=core;g=ENSRNOG00000020105) | [9](http://www.ensembl.org/Rattus_norvegicus/mapview?chr=9) | [90580293](http://www.ensembl.org/Rattus_norvegicus/contigview?chr=9&vc_start=90580293&vc_end=90587399) | [90587399](http://www.ensembl.org/Rattus_norvegicus/contigview?chr=9&vc_start=90580293&vc_end=90587399) | [Klhl30](http://www.ensembl.org/Rattus_norvegicus/geneview?gene=Klhl30) |  |
|  | [ENSRNOG00000024688](http://www.ensembl.org/Rattus_norvegicus/Gene/Summary?db=core;g=ENSRNOG00000024688) | [9](http://www.ensembl.org/Rattus_norvegicus/mapview?chr=9) | [90593588](http://www.ensembl.org/Rattus_norvegicus/contigview?chr=9&vc_start=90593588&vc_end=90599656) | [90599656](http://www.ensembl.org/Rattus_norvegicus/contigview?chr=9&vc_start=90593588&vc_end=90599656) | [Fam132b](http://www.ensembl.org/Rattus_norvegicus/geneview?gene=Fam132b) |  |
|  | [ENSRNOG00000024468](http://www.ensembl.org/Rattus_norvegicus/Gene/Summary?db=core;g=ENSRNOG00000024468) | [9](http://www.ensembl.org/Rattus_norvegicus/mapview?chr=9) | [90711965](http://www.ensembl.org/Rattus_norvegicus/contigview?chr=9&vc_start=90711965&vc_end=90747305) | [90747305](http://www.ensembl.org/Rattus_norvegicus/contigview?chr=9&vc_start=90711965&vc_end=90747305) | [MIPT3_RAT](http://www.ensembl.org/Rattus_norvegicus/geneview?gene=MIPT3_RAT) | TRAF3-interacting protein 1 |
|  | [ENSRNOG00000020322](http://www.ensembl.org/Rattus_norvegicus/Gene/Summary?db=core;g=ENSRNOG00000020322) | [9](http://www.ensembl.org/Rattus_norvegicus/mapview?chr=9) | [90758693](http://www.ensembl.org/Rattus_norvegicus/contigview?chr=9&vc_start=90758693&vc_end=90774541) | [90774541](http://www.ensembl.org/Rattus_norvegicus/contigview?chr=9&vc_start=90758693&vc_end=90774541) | [NP_001101702.1](http://www.ensembl.org/Rattus_norvegicus/geneview?gene=NP_001101702.1) | ankyrin repeat and SOCS box-containing 1 |
|  | [ENSRNOG00000020355](http://www.ensembl.org/Rattus_norvegicus/Gene/Summary?db=core;g=ENSRNOG00000020355) | [9](http://www.ensembl.org/Rattus_norvegicus/mapview?chr=9) | [91016450](http://www.ensembl.org/Rattus_norvegicus/contigview?chr=9&vc_start=91016450&vc_end=91058863) | [91058863](http://www.ensembl.org/Rattus_norvegicus/contigview?chr=9&vc_start=91016450&vc_end=91058863) | [TWST2_RAT](http://www.ensembl.org/Rattus_norvegicus/geneview?gene=TWST2_RAT) | Twist-related protein 2 |
|  | [ENSRNOG00000037403](http://www.ensembl.org/Rattus_norvegicus/Gene/Summary?db=core;g=ENSRNOG00000037403) | [9](http://www.ensembl.org/Rattus_norvegicus/mapview?chr=9) | [91840207](http://www.ensembl.org/Rattus_norvegicus/contigview?chr=9&vc_start=91840207&vc_end=91841261) | [91841261](http://www.ensembl.org/Rattus_norvegicus/contigview?chr=9&vc_start=91840207&vc_end=91841261) | [NP_001000750.1](http://www.ensembl.org/Rattus_norvegicus/geneview?gene=NP_001000750.1) | olfactory receptor Olr1353 |
|  | [ENSRNOG00000004452](http://www.ensembl.org/Rattus_norvegicus/Gene/Summary?db=core;g=ENSRNOG00000004452) | [9](http://www.ensembl.org/Rattus_norvegicus/mapview?chr=9) | [92277514](http://www.ensembl.org/Rattus_norvegicus/contigview?chr=9&vc_start=92277514&vc_end=92282986) | [92282986](http://www.ensembl.org/Rattus_norvegicus/contigview?chr=9&vc_start=92277514&vc_end=92282986) | [NP_001102479.1](http://www.ensembl.org/Rattus_norvegicus/geneview?gene=NP_001102479.1) | aquaporin 12B |
|  | [ENSRNOG00000023856](http://www.ensembl.org/Rattus_norvegicus/Gene/Summary?db=core;g=ENSRNOG00000023856) | [9](http://www.ensembl.org/Rattus_norvegicus/mapview?chr=9) | [92412128](http://www.ensembl.org/Rattus_norvegicus/contigview?chr=9&vc_start=92412128&vc_end=92422075) | [92422075](http://www.ensembl.org/Rattus_norvegicus/contigview?chr=9&vc_start=92412128&vc_end=92422075) | [SPYA_RAT](http://www.ensembl.org/Rattus_norvegicus/geneview?gene=SPYA_RAT) | Serine--pyruvate aminotransferase, mitochondrial Precursor |
|  | [ENSRNOG00000032920](http://www.ensembl.org/Rattus_norvegicus/Gene/Summary?db=core;g=ENSRNOG00000032920) | [9](http://www.ensembl.org/Rattus_norvegicus/mapview?chr=9) | [92456878](http://www.ensembl.org/Rattus_norvegicus/contigview?chr=9&vc_start=92456878&vc_end=92503001) | [92503001](http://www.ensembl.org/Rattus_norvegicus/contigview?chr=9&vc_start=92456878&vc_end=92503001) |  |  |
|  | [ENSRNOG00000023548](http://www.ensembl.org/Rattus_norvegicus/Gene/Summary?db=core;g=ENSRNOG00000023548) | [9](http://www.ensembl.org/Rattus_norvegicus/mapview?chr=9) | [92509650](http://www.ensembl.org/Rattus_norvegicus/contigview?chr=9&vc_start=92509650&vc_end=92567345) | [92567345](http://www.ensembl.org/Rattus_norvegicus/contigview?chr=9&vc_start=92509650&vc_end=92567345) | [SNED1_RAT](http://www.ensembl.org/Rattus_norvegicus/geneview?gene=SNED1_RAT) | Sushi, nidogen and EGF-like domain-containing protein 1 Precursor |
|  | [ENSRNOG00000016974](http://www.ensembl.org/Rattus_norvegicus/Gene/Summary?db=core;g=ENSRNOG00000016974) | [9](http://www.ensembl.org/Rattus_norvegicus/mapview?chr=9) | [92624083](http://www.ensembl.org/Rattus_norvegicus/contigview?chr=9&vc_start=92624083&vc_end=92648033) | [92648033](http://www.ensembl.org/Rattus_norvegicus/contigview?chr=9&vc_start=92624083&vc_end=92648033) | [PP1R7_RAT](http://www.ensembl.org/Rattus_norvegicus/geneview?gene=PP1R7_RAT) | Protein phosphatase 1 regulatory subunit 7 |
|  | [ENSRNOG00000023427](http://www.ensembl.org/Rattus_norvegicus/Gene/Summary?db=core;g=ENSRNOG00000023427) | [9](http://www.ensembl.org/Rattus_norvegicus/mapview?chr=9) | [92655567](http://www.ensembl.org/Rattus_norvegicus/contigview?chr=9&vc_start=92655567&vc_end=92683022) | [92683022](http://www.ensembl.org/Rattus_norvegicus/contigview?chr=9&vc_start=92655567&vc_end=92683022) | [ANO7_RAT](http://www.ensembl.org/Rattus_norvegicus/geneview?gene=ANO7_RAT) | Anoctamin-7 (Transmembrane protein 16G) |
|  | [ENSRNOG00000017952](http://www.ensembl.org/Rattus_norvegicus/Gene/Summary?db=core;g=ENSRNOG00000017952) | [9](http://www.ensembl.org/Rattus_norvegicus/mapview?chr=9) | [92756091](http://www.ensembl.org/Rattus_norvegicus/contigview?chr=9&vc_start=92756091&vc_end=92789254) | [92789254](http://www.ensembl.org/Rattus_norvegicus/contigview?chr=9&vc_start=92756091&vc_end=92789254) | [SEPT2_RAT](http://www.ensembl.org/Rattus_norvegicus/geneview?gene=SEPT2_RAT) | Septin-2 (Vascular endothelial cell specific protein 11) |
|  | [ENSRNOG00000018051](http://www.ensembl.org/Rattus_norvegicus/Gene/Summary?db=core;g=ENSRNOG00000018051) | [9](http://www.ensembl.org/Rattus_norvegicus/mapview?chr=9) | [92792360](http://www.ensembl.org/Rattus_norvegicus/contigview?chr=9&vc_start=92792360&vc_end=92908126) | [92908126](http://www.ensembl.org/Rattus_norvegicus/contigview?chr=9&vc_start=92792360&vc_end=92908126) | [NP_001101703.1](http://www.ensembl.org/Rattus_norvegicus/geneview?gene=NP_001101703.1) | FERM, RhoGEF and pleckstrin domain protein 2 |
|  | [ENSRNOG00000018214](http://www.ensembl.org/Rattus_norvegicus/Gene/Summary?db=core;g=ENSRNOG00000018214) | [9](http://www.ensembl.org/Rattus_norvegicus/mapview?chr=9) | [92970069](http://www.ensembl.org/Rattus_norvegicus/contigview?chr=9&vc_start=92970069&vc_end=92980973) | [92980973](http://www.ensembl.org/Rattus_norvegicus/contigview?chr=9&vc_start=92970069&vc_end=92980973) | [BOK_RAT](http://www.ensembl.org/Rattus_norvegicus/geneview?gene=BOK_RAT) | Bcl-2-related ovarian killer protein |
|  | [ENSRNOG00000018403](http://www.ensembl.org/Rattus_norvegicus/Gene/Summary?db=core;g=ENSRNOG00000018403) | [9](http://www.ensembl.org/Rattus_norvegicus/mapview?chr=9) | [93044474](http://www.ensembl.org/Rattus_norvegicus/contigview?chr=9&vc_start=93044474&vc_end=93063882) | [93063882](http://www.ensembl.org/Rattus_norvegicus/contigview?chr=9&vc_start=93044474&vc_end=93063882) | [NP_001020882.1](http://www.ensembl.org/Rattus_norvegicus/geneview?gene=NP_001020882.1) | APG4 (ATG4) autophagy-related homolog B |
|  | [ENSRNOG00000018988](http://www.ensembl.org/Rattus_norvegicus/Gene/Summary?db=core;g=ENSRNOG00000018988) | [9](http://www.ensembl.org/Rattus_norvegicus/mapview?chr=9) | [93076358](http://www.ensembl.org/Rattus_norvegicus/contigview?chr=9&vc_start=93076358&vc_end=93094002) | [93094002](http://www.ensembl.org/Rattus_norvegicus/contigview?chr=9&vc_start=93076358&vc_end=93094002) | [NP_001102280.1](http://www.ensembl.org/Rattus_norvegicus/geneview?gene=NP_001102280.1) | inhibitor of growth family, member 5 |
|  | [ENSRNOG00000019012](http://www.ensembl.org/Rattus_norvegicus/Gene/Summary?db=core;g=ENSRNOG00000019012) | [9](http://www.ensembl.org/Rattus_norvegicus/mapview?chr=9) | [93100369](http://www.ensembl.org/Rattus_norvegicus/contigview?chr=9&vc_start=93100369&vc_end=93119170) | [93119170](http://www.ensembl.org/Rattus_norvegicus/contigview?chr=9&vc_start=93100369&vc_end=93119170) | [D2HDH_RAT](http://www.ensembl.org/Rattus_norvegicus/geneview?gene=D2HDH_RAT) | D-2-hydroxyglutarate dehydrogenase, mitochondrial Precursor |
|  | [ENSRNOG00000022982](http://www.ensembl.org/Rattus_norvegicus/Gene/Summary?db=core;g=ENSRNOG00000022982) | [9](http://www.ensembl.org/Rattus_norvegicus/mapview?chr=9) | [93137424](http://www.ensembl.org/Rattus_norvegicus/contigview?chr=9&vc_start=93137424&vc_end=93140436) | [93140436](http://www.ensembl.org/Rattus_norvegicus/contigview?chr=9&vc_start=93137424&vc_end=93140436) | [LOC685402](http://www.ensembl.org/Rattus_norvegicus/geneview?gene=LOC685402) |  |
|  | [ENSRNOG00000019031](http://www.ensembl.org/Rattus_norvegicus/Gene/Summary?db=core;g=ENSRNOG00000019031) | [9](http://www.ensembl.org/Rattus_norvegicus/mapview?chr=9) | [93150680](http://www.ensembl.org/Rattus_norvegicus/contigview?chr=9&vc_start=93150680&vc_end=93156336) | [93156336](http://www.ensembl.org/Rattus_norvegicus/contigview?chr=9&vc_start=93150680&vc_end=93156336) | [NP_001101704.1](http://www.ensembl.org/Rattus_norvegicus/geneview?gene=NP_001101704.1) | sialidase 4 |
|  | [ENSRNOG00000019087](http://www.ensembl.org/Rattus_norvegicus/Gene/Summary?db=core;g=ENSRNOG00000019087) | [9](http://www.ensembl.org/Rattus_norvegicus/mapview?chr=9) | [94575773](http://www.ensembl.org/Rattus_norvegicus/contigview?chr=9&vc_start=94575773&vc_end=94593927) | [94593927](http://www.ensembl.org/Rattus_norvegicus/contigview?chr=9&vc_start=94575773&vc_end=94593927) | [F174A_RAT](http://www.ensembl.org/Rattus_norvegicus/geneview?gene=F174A_RAT) | Membrane protein FAM174A Precursor |
|  | [ENSRNOG00000043322](http://www.ensembl.org/Rattus_norvegicus/Gene/Summary?db=core;g=ENSRNOG00000043322) | [9](http://www.ensembl.org/Rattus_norvegicus/mapview?chr=9) | [94820266](http://www.ensembl.org/Rattus_norvegicus/contigview?chr=9&vc_start=94820266&vc_end=94859691) | [94859691](http://www.ensembl.org/Rattus_norvegicus/contigview?chr=9&vc_start=94820266&vc_end=94859691) | [St8sia4](http://www.ensembl.org/Rattus_norvegicus/geneview?gene=St8sia4) | CMP-N-acetylneuraminate-poly-alpha-2,8-sialyltransferase |
|  | [ENSRNOG00000029081](http://www.ensembl.org/Rattus_norvegicus/Gene/Summary?db=core;g=ENSRNOG00000029081) | [9](http://www.ensembl.org/Rattus_norvegicus/mapview?chr=9) | [96760390](http://www.ensembl.org/Rattus_norvegicus/contigview?chr=9&vc_start=96760390&vc_end=96760894) | [96760894](http://www.ensembl.org/Rattus_norvegicus/contigview?chr=9&vc_start=96760390&vc_end=96760894) | [IPI00566979.1](http://www.ensembl.org/Rattus_norvegicus/geneview?gene=IPI00566979.1) |  |
|  | [ENSRNOG00000033280](http://www.ensembl.org/Rattus_norvegicus/Gene/Summary?db=core;g=ENSRNOG00000033280) | [9](http://www.ensembl.org/Rattus_norvegicus/mapview?chr=9) | [96893071](http://www.ensembl.org/Rattus_norvegicus/contigview?chr=9&vc_start=96893071&vc_end=97046865) | [97046865](http://www.ensembl.org/Rattus_norvegicus/contigview?chr=9&vc_start=96893071&vc_end=97046865) | [AMD_RAT](http://www.ensembl.org/Rattus_norvegicus/geneview?gene=AMD_RAT) | Peptidyl-glycine alpha-amidating monooxygenase Precursor |
|  | [ENSRNOG00000011613](http://www.ensembl.org/Rattus_norvegicus/Gene/Summary?db=core;g=ENSRNOG00000011613) | [9](http://www.ensembl.org/Rattus_norvegicus/mapview?chr=9) | [97103538](http://www.ensembl.org/Rattus_norvegicus/contigview?chr=9&vc_start=97103538&vc_end=97174156) | [97174156](http://www.ensembl.org/Rattus_norvegicus/contigview?chr=9&vc_start=97103538&vc_end=97174156) | [Q5XI45_RAT](http://www.ensembl.org/Rattus_norvegicus/geneview?gene=Q5XI45_RAT) | RGD1308143 protein Fragment |
|  | [ENSRNOG00000032398](http://www.ensembl.org/Rattus_norvegicus/Gene/Summary?db=core;g=ENSRNOG00000032398) | [9](http://www.ensembl.org/Rattus_norvegicus/mapview?chr=9) | [97216277](http://www.ensembl.org/Rattus_norvegicus/contigview?chr=9&vc_start=97216277&vc_end=97236238) | [97236238](http://www.ensembl.org/Rattus_norvegicus/contigview?chr=9&vc_start=97216277&vc_end=97236238) | [NP_001102556.1](http://www.ensembl.org/Rattus_norvegicus/geneview?gene=NP_001102556.1) | hypothetical protein LOC501195 |
|  | [ENSRNOG00000017256](http://www.ensembl.org/Rattus_norvegicus/Gene/Summary?db=core;g=ENSRNOG00000017256) | [9](http://www.ensembl.org/Rattus_norvegicus/mapview?chr=9) | [84229710](http://www.ensembl.org/Rattus_norvegicus/contigview?chr=9&vc_start=84229710&vc_end=84230624) | [84230624](http://www.ensembl.org/Rattus_norvegicus/contigview?chr=9&vc_start=84229710&vc_end=84230624) | [Csprs](http://www.ensembl.org/Rattus_norvegicus/geneview?gene=Csprs) |  |
|  | [ENSRNOG00000018559](http://www.ensembl.org/Rattus_norvegicus/Gene/Summary?db=core;g=ENSRNOG00000018559) | [9](http://www.ensembl.org/Rattus_norvegicus/mapview?chr=9) | [85189062](http://www.ensembl.org/Rattus_norvegicus/contigview?chr=9&vc_start=85189062&vc_end=85190480) | [85190480](http://www.ensembl.org/Rattus_norvegicus/contigview?chr=9&vc_start=85189062&vc_end=85190480) | [CB057_RAT](http://www.ensembl.org/Rattus_norvegicus/geneview?gene=CB057_RAT) | Uncharacterized protein C2orf57 homolog |
|  | [ENSRNOG00000030454](http://www.ensembl.org/Rattus_norvegicus/Gene/Summary?db=core;g=ENSRNOG00000030454) | [9](http://www.ensembl.org/Rattus_norvegicus/mapview?chr=9) | [91765288](http://www.ensembl.org/Rattus_norvegicus/contigview?chr=9&vc_start=91765288&vc_end=91766259) | [91766259](http://www.ensembl.org/Rattus_norvegicus/contigview?chr=9&vc_start=91765288&vc_end=91766259) | [NP_001000520.1](http://www.ensembl.org/Rattus_norvegicus/geneview?gene=NP_001000520.1) | olfactory receptor Olr1346 |
|  | [ENSRNOG00000032255](http://www.ensembl.org/Rattus_norvegicus/Gene/Summary?db=core;g=ENSRNOG00000032255) | [9](http://www.ensembl.org/Rattus_norvegicus/mapview?chr=9) | [91779443](http://www.ensembl.org/Rattus_norvegicus/contigview?chr=9&vc_start=91779443&vc_end=91780414) | [91780414](http://www.ensembl.org/Rattus_norvegicus/contigview?chr=9&vc_start=91779443&vc_end=91780414) | [NP_001001121.1](http://www.ensembl.org/Rattus_norvegicus/geneview?gene=NP_001001121.1) | olfactory receptor Olr1347 |
|  | [ENSRNOG00000016546](http://www.ensembl.org/Rattus_norvegicus/Gene/Summary?db=core;g=ENSRNOG00000016546) | [9](http://www.ensembl.org/Rattus_norvegicus/mapview?chr=9) | [91790150](http://www.ensembl.org/Rattus_norvegicus/contigview?chr=9&vc_start=91790150&vc_end=91791109) | [91791109](http://www.ensembl.org/Rattus_norvegicus/contigview?chr=9&vc_start=91790150&vc_end=91791109) | [NP_001000484.1](http://www.ensembl.org/Rattus_norvegicus/geneview?gene=NP_001000484.1) | olfactory receptor Olr1349 |
|  | [ENSRNOG00000030424](http://www.ensembl.org/Rattus_norvegicus/Gene/Summary?db=core;g=ENSRNOG00000030424) | [9](http://www.ensembl.org/Rattus_norvegicus/mapview?chr=9) | [91797535](http://www.ensembl.org/Rattus_norvegicus/contigview?chr=9&vc_start=91797535&vc_end=91798506) | [91798506](http://www.ensembl.org/Rattus_norvegicus/contigview?chr=9&vc_start=91797535&vc_end=91798506) | [NP_001000752.1](http://www.ensembl.org/Rattus_norvegicus/geneview?gene=NP_001000752.1) | olfactory receptor Olr1350 |
|  | [ENSRNOG00000032545](http://www.ensembl.org/Rattus_norvegicus/Gene/Summary?db=core;g=ENSRNOG00000032545) | [9](http://www.ensembl.org/Rattus_norvegicus/mapview?chr=9) | [91808204](http://www.ensembl.org/Rattus_norvegicus/contigview?chr=9&vc_start=91808204&vc_end=91809169) | [91809169](http://www.ensembl.org/Rattus_norvegicus/contigview?chr=9&vc_start=91808204&vc_end=91809169) | [NP_001000751.1](http://www.ensembl.org/Rattus_norvegicus/geneview?gene=NP_001000751.1) | olfactory receptor Olr1351 |
|  | [ENSRNOG00000037404](http://www.ensembl.org/Rattus_norvegicus/Gene/Summary?db=core;g=ENSRNOG00000037404) | [9](http://www.ensembl.org/Rattus_norvegicus/mapview?chr=9) | [91821321](http://www.ensembl.org/Rattus_norvegicus/contigview?chr=9&vc_start=91821321&vc_end=91822289) | [91822289](http://www.ensembl.org/Rattus_norvegicus/contigview?chr=9&vc_start=91821321&vc_end=91822289) | [NP_001000953.1](http://www.ensembl.org/Rattus_norvegicus/geneview?gene=NP_001000953.1) | olfactory receptor Olr1352 |
|  | [ENSRNOG00000024030](http://www.ensembl.org/Rattus_norvegicus/Gene/Summary?db=core;g=ENSRNOG00000024030) | [9](http://www.ensembl.org/Rattus_norvegicus/mapview?chr=9) | [92258279](http://www.ensembl.org/Rattus_norvegicus/contigview?chr=9&vc_start=92258279&vc_end=92259199) | [92259199](http://www.ensembl.org/Rattus_norvegicus/contigview?chr=9&vc_start=92258279&vc_end=92259199) | [NP_001032436.1](http://www.ensembl.org/Rattus_norvegicus/geneview?gene=NP_001032436.1) | G protein-coupled receptor 35 |
|  | [ENSRNOG00000037347](http://www.ensembl.org/Rattus_norvegicus/Gene/Summary?db=core;g=ENSRNOG00000037347) | [9](http://www.ensembl.org/Rattus_norvegicus/mapview?chr=9) | [93028352](http://www.ensembl.org/Rattus_norvegicus/contigview?chr=9&vc_start=93028352&vc_end=93028588) | [93028588](http://www.ensembl.org/Rattus_norvegicus/contigview?chr=9&vc_start=93028352&vc_end=93028588) | [IPI00780654.1](http://www.ensembl.org/Rattus_norvegicus/geneview?gene=IPI00780654.1) |  |
|  | [ENSRNOG00000032765](http://www.ensembl.org/Rattus_norvegicus/Gene/Summary?db=core;g=ENSRNOG00000032765) | [9](http://www.ensembl.org/Rattus_norvegicus/mapview?chr=9) | [97909264](http://www.ensembl.org/Rattus_norvegicus/contigview?chr=9&vc_start=97909264&vc_end=97909705) | [97909705](http://www.ensembl.org/Rattus_norvegicus/contigview?chr=9&vc_start=97909264&vc_end=97909705) | [RGD1559955](http://www.ensembl.org/Rattus_norvegicus/geneview?gene=RGD1559955) |  |
|  | [ENSRNOG00000022614](http://www.ensembl.org/Rattus_norvegicus/Gene/Summary?db=core;g=ENSRNOG00000022614) | [9](http://www.ensembl.org/Rattus_norvegicus/mapview?chr=9) | [84713025](http://www.ensembl.org/Rattus_norvegicus/contigview?chr=9&vc_start=84713025&vc_end=84713354) | [84713354](http://www.ensembl.org/Rattus_norvegicus/contigview?chr=9&vc_start=84713025&vc_end=84713354) |  |  |

**Table S9**: Phenotyping scheme

| **Age (PND)** | **Test** | **Age(PND)** | **Test** |
| --- | --- | --- | --- |
| -7 | handling | -7 | handling |
| -7 | handling | -7 | handling |
| 62-80 | 3 day-Phenotyper test (D/L) | 100-120 | WAT isolation |
| +3 | 10 day–Phenotyper test (D/D) |  | |
| +20 | handling |  | |
| +27 | handling |  | |
| +34 | Phenotyper test – cocaine challenge |  | |
| Age is given in postnatal days (PND). D/L reflect the 3-day 12 hr day/light phase, and D/D reflects the 10-day continuous dark phase. | | | |

**Table S10**: Traits measured in the F2 animals.

| **Parameters - Phenotyper** | **Parameters -Cocaine** | **Parameters -WAT** |
| --- | --- | --- |
| Time in Centre-3 days | Time in centre 0-120 min | Abdominal WAT/100 gram |
| Time in Shelter-3 days | Distance moved 1^st^ 10 min | Subcutaneous WAT/100 gram |
| Distance Moved-3 days | Distance moved 2^nd^ 10 min |  |
| Duration of immobility-3 days | Distance moved 30-120 min |  |
| Duration of mobility-3 days |  |  |
| Duration of strong mobility-3 days |  |  |
| Parameters are cumulative over the given time frames. Immobility is defined as < 15% change in movement, mobility is defined as > 15% < 60% change in movement, and strong mobility includes > 60% changes in movement. | | |

**Figure S1**

**
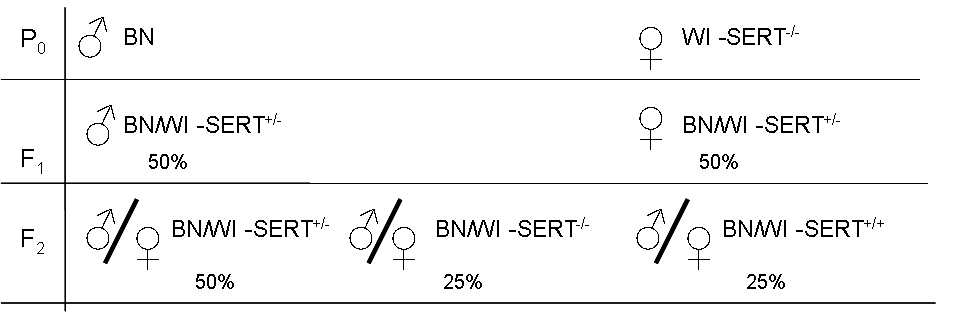
**

Set-up of the modifier screen. WI = Wistar; BN = Brown Norway; SERT^+/+^ = SERT wild type; SERT^-/-^ = SERT homozygous knockout; SERT^+/-^ = SERT heterozygous knockout.
